# Performance and limitations of four large language models in genetic counseling for thalassemia

**DOI:** 10.3389/fdgth.2026.1902480

**Published:** 2026-08-11

**Authors:** Wenfu Zhong, Jingwen Huang, Mengsi Wei, Qingpeng Liang, Yuanwu Yang, Jinrong Chen, Jinjiang Mao, Ying Qin, Yifan Sun, Yishan Liang

**Affiliations:** 1 Department of Clinical Laboratory, Guigang Clinical Medical Research Center for Medical Laboratory, Guigang City People's Hospital, Guigang, Guangxi, China; 2 Department of Respiratory and Critical Care Medicine, Guigang City People's Hospital, Guigang, Guangxi, China; 3 Department of Obstetrics, Guigang City People's Hospital, Guigang, Guangxi, China

**Keywords:** genetic counseling, large language models, performance evaluation, prenatal diagnosis, thalassemia

## Abstract

In regions with high thalassemia prevalence, such as southern China and Southeast Asia, chronic shortages of professional genetic counseling resources have driven interest in large language models (LLMs) as auxiliary tools, yet their performance and safety boundaries in this setting remain uncharacterized. This single-center retrospective study evaluated four LLMs (ChatGPT-5.2 Thinking, DeepSeek-V3.2 chat, Gemini 3 Flash, and Grok 4.1 Fast) using 1,080 standardized knowledge questions administered across five independent sessions and 150 real-world clinical cases scored by six senior experts across eight dimensions. All models exceeded 90% accuracy on single-choice and true-false questions. Between-model differences were most pronounced in multiple-choice questions, where ChatGPT-5.2 Thinking achieved the highest accuracy (87.28% ± 1.69%), significantly outperforming Grok 4.1 Fast (72.06% ± 1.69%, *P* < 0.001). DeepSeek-V3.2 chat showed lower cross-session consistency than the other three models. In clinical case analysis, all models scored below human expert levels overall, with ChatGPT-5.2 Thinking performing closest to experts. Test report interpretation was generally adequate, whereas larger gaps emerged in genetic risk estimation, phenotype prediction, and counseling recommendations (all *P* < 0.001 vs. human experts, with limited exceptions for ChatGPT-5.2 Thinking in *β*-thalassemia subtypes). All 145 severe errors were confined to *α*-thalassemia and *α*-combined-*β*-thalassemia subtypes, concentrated in phenotype prediction (76/145, 52.4%) and risk estimation (40/145, 27.6%). LLMs may support knowledge retrieval and structured report interpretation in thalassemia genetic counseling but should not be used independently for risk assessment or final counseling decisions, particularly in complex *α*-related cases.

## 1 Introduction

Thalassemia is one of the most common monogenic disorders worldwide, with particularly high prevalence in southern China, Southeast Asia, South Asia, and the Middle East (1–3). Severe forms require lifelong blood transfusions and iron chelation therapy, imposing substantial disease burden and healthcare costs (4). Reducing the birth rate of severely affected children through pre-pregnancy screening, prenatal diagnosis, and informed reproductive decision-making remains the cornerstone of prevention and control (5, 6). Genetic counseling is central to this preventive system. Counselors must accurately interpret complete blood counts, hemoglobin analyses, and genetic test results, then estimate genetic risks, predict fetal phenotypes, and guide reproductive decisions within each family's clinical context (7, 8). This process is considerably more demanding in *α*-thalassemia and α-combined-*β*-thalassemia than in pure *β*-thalassemia, as the genotype-phenotype relationship is shaped by deletional and non-deletional variants, modifier genes, and the coverage of available testing platforms (9–11). Professional genetic counseling resources remain chronically insufficient in high-prevalence regions, and this disparity severely limits patient access to timely, accurate counseling (12, 13).

Large language models (LLMs), with their natural language understanding and knowledge integration capabilities, have shown potential across several clinical domains, including documentation, medical education, and patient question-answering (14, 15). Prior studies indicate that LLMs have preliminary value in gynecologic oncology genetic counseling and hemoglobinopathy test interpretation, and GPT-4 has demonstrated utility in physician-assisted patient management reasoning (16–18). These studies, however, were limited to narrow task definitions and did not examine whether LLMs can reliably perform the complete counseling process in thalassemia, a setting that requires multi-step probabilistic reasoning across genetically heterogeneous subtypes. Prior studies have consistently identified notable limitations of LLMs in complex genetic disease contexts, including unstable phenotype recognition and unreliable probabilistic inference (19–21), and no study to date has systematically evaluated their performance or defined their safety boundaries specifically for thalassemia genetic counseling (22, 23).

The present study investigated these questions using a dual-track evaluation framework comprising 1,080 standardized knowledge questions administered across five independent sessions to capture cross-session consistency, and 150 retrospectively collected, fully de-identified real-world clinical cases with all personal identifiers removed across eight scoring dimensions. Using senior genetic counselors as a reference standard, we compared four mainstream LLMs (ChatGPT-5.2 Thinking, DeepSeek-V3.2 chat, Gemini 3 Flash, and Grok 4.1 Fast) across knowledge accuracy, output stability, performance by task and subtype, and the distribution of severe errors. The findings are intended to inform the safe and standardized integration of LLMs into thalassemia genetic counseling practice in high-prevalence regions.

## 2 Materials and methods

### 2.1 Study design and ethics approval

This single-center retrospective study was conducted between December 2025 and January 2026 at Guigang People's Hospital. The study followed the principles of the Declaration of Helsinki and was approved by the Ethics Committee of Guigang People's Hospital (approval No. 20250023).

All clinical case data were retrospectively extracted from archived routine thalassemia test reports in the hospital's electronic medical record system. Before inputting data into online large language models for testing, strict privacy de-identification was implemented: all direct personal identifiable information, including patient full names, outpatient/inpatient visit numbers, residential addresses, and contact phone numbers, was permanently deleted from every case record. Only de-identified laboratory test indicators, thalassemia genotyping results and couple pairing information required for genetic counseling analysis were retained. No multi-source cross-referential data that might enable patient re-identification was preserved. The de-identified case information transmitted to LLM online interfaces is identical to the case summary data provided in supplementary materials, and no extra identifiable personal genetic metadata was uploaded to external servers.

Given the fully de-identified retrospective design with no additional clinical intervention or patient contact, the study protocol was formally granted a waiver of individual written informed consent by the Ethics Committee of Guigang People's Hospital, in full compliance with the Declaration of Helsinki and national ethical regulations for minimal-risk biomedical research.

### 2.2 Construction of test datasets

Two test sets were constructed to evaluate domain knowledge and real-world counseling performance. The knowledge test set was derived from the institutional standardized training textbook *Prevention and Treatment of Thalassemia* (24) and comprised 1,080 questions: 600 single-choice, 240 multiple-choice, and 240 true-false. Content covered molecular mechanisms, inheritance patterns, laboratory testing, result interpretation, and prevention strategies. All questions were reviewed and approved by three senior genetic counseling experts.

The case test set retrospectively included 150 couples who underwent thalassemia genetic counseling at the Prenatal Diagnosis Center of Guigang People's Hospital between 2023 and 2025. Eligible couples had complete medical histories, complete blood counts, hemoglobin analyses, and thalassemia genetic test results for both partners. Cases were excluded if critical clinical information was missing or if inheritance patterns or genotype-phenotype relationships were atypical or unclear. Based on offspring risk type and severity, cases were divided into six groups: mild *α*-thalassemia (*n* = 30), moderate-to-severe *α*-thalassemia (*n* = 20), mild *β*-thalassemia (*n* = 20), moderate-to-severe *β*-thalassemia (*n* = 20), mild *α*-combined-*β*-thalassemia (*n* = 20), and moderate-to-severe *α*-combined-*β*-thalassemia (*n* = 40).

### 2.3 Models and inference settings

Four mainstream LLMs were evaluated: ChatGPT-5.2 Thinking (OpenAI, USA), DeepSeek-V3.2 chat (DeepSeek, China), Gemini 3 Flash (Google, USA), and Grok 4.1 Fast (xAI, USA). All models were accessed via official application programming interfaces between December 2025 and January 2026, using the latest stable versions available during this period. To ensure determinism and reproducibility, the temperature parameter was set to 0, top_p to 1, and random sampling was disabled. Each test session was reset immediately after completion to eliminate contextual interference between cases.

### 2.4 Evaluation procedures and scoring criteria

#### 2.4.1 Knowledge testing

The 1,080 questions were divided into 108 groups of 10 questions each. A standardized Chinese prompt instructed each model to respond as a senior genetic counselor and to present answers in tabular format. Each round of testing was completed within a single day and repeated after a 3-day interval, for a total of five independent rounds. Evaluation metrics included accuracy (proportion of correct answers) and cross-session consistency (proportion of questions answered identically across all five rounds).

#### 2.4.2 Case testing

For each case, complete clinical data and test reports were compiled into a PDF file and submitted to each model using a standardized Chinese prompt. The model was required to complete four tasks per case: test report interpretation, genetic risk estimation, clinical phenotype prediction, and genetic counseling recommendations. Cases were tested individually, and each session was reset immediately after completion to eliminate contextual interference.

#### 2.4.3 Scoring criteria and quality control

Six senior genetic counselors with over 20 years of experience in thalassemia genetic counseling served as scoring experts. Before scoring, all experts completed a one-day standardized training session followed by pre-scoring on 10 pilot cases, yielding good inter-rater reliability (ICC = 0.92, 95% CI 0.87–0.95, *P* < 0.001). A standardized scoring rubric was developed based on a 5-point framework used in previous rare disease genetic counseling evaluations (22), adapted to the clinical workflow of thalassemia genetic counseling. Scores were assigned for both accuracy and completeness across the four tasks, yielding eight evaluation dimensions in total. Scores were defined as follows: 5, fully consistent with clinical guidelines and medical facts; 4, minor deficiencies not affecting core information accuracy; 3, some errors but core clinical information still acceptable; 2, significant errors that could mislead clinical decisions; 1, major errors completely inconsistent with clinical standards. A score of 2 or below in any dimension was defined as a severe error, requiring unanimous agreement among all six scoring experts. Notably, strict scorer blinding to model identity was not feasible in this study. All LLM responses were exported as original PDF files or full-page screenshots to preserve the full integrity of structured output content, including formatted tables, hierarchical reasoning explanations, and supplementary clinical annotations. Each LLM platform has unique, inherent formatting features and interface identifiers that cannot be fully removed without altering, truncating, or distorting the original response content. To mitigate potential scoring bias, all cases were shuffled in random order before distribution to scorers, and all scoring was performed independently by the six experts based on the objective, dimension-specific rubric described above. The human expert reference standard was established by two senior genetic counselors (each with over 10 years of experience in thalassemia genetic counseling) who independently completed the same 150 cases; their responses were scored by two of the scoring experts using the same rubric.

### 2.5 Statistical analysis

All analyses were performed using GraphPad Prism 10.0 and R 4.4.0. Continuous variables are presented as mean ± standard deviation and categorical variables as counts and percentages. For knowledge testing, between-group accuracy comparisons were performed using one-way analysis of variance (ANOVA) with Bonferroni correction for multiple comparisons. For clinical case scores, between-model differences were assessed using a linear mixed-effects model with model type (LLMs vs. human experts) as a fixed effect, case ID and scoring expert as random effects, and thalassemia subtype group as a covariate. Comparisons between each LLM and human experts were performed as *post-hoc* contrasts within the same model framework, using human experts as the reference group. The distribution of severe errors was analyzed descriptively. A two-sided significance level of 0.05 was applied throughout, and *P* < 0.05 was considered statistically significant.

## 3 Results

### 3.1 Performance of the four LLMs in medical knowledge testing

#### 3.1.1 Accuracy

**Figure 1:**
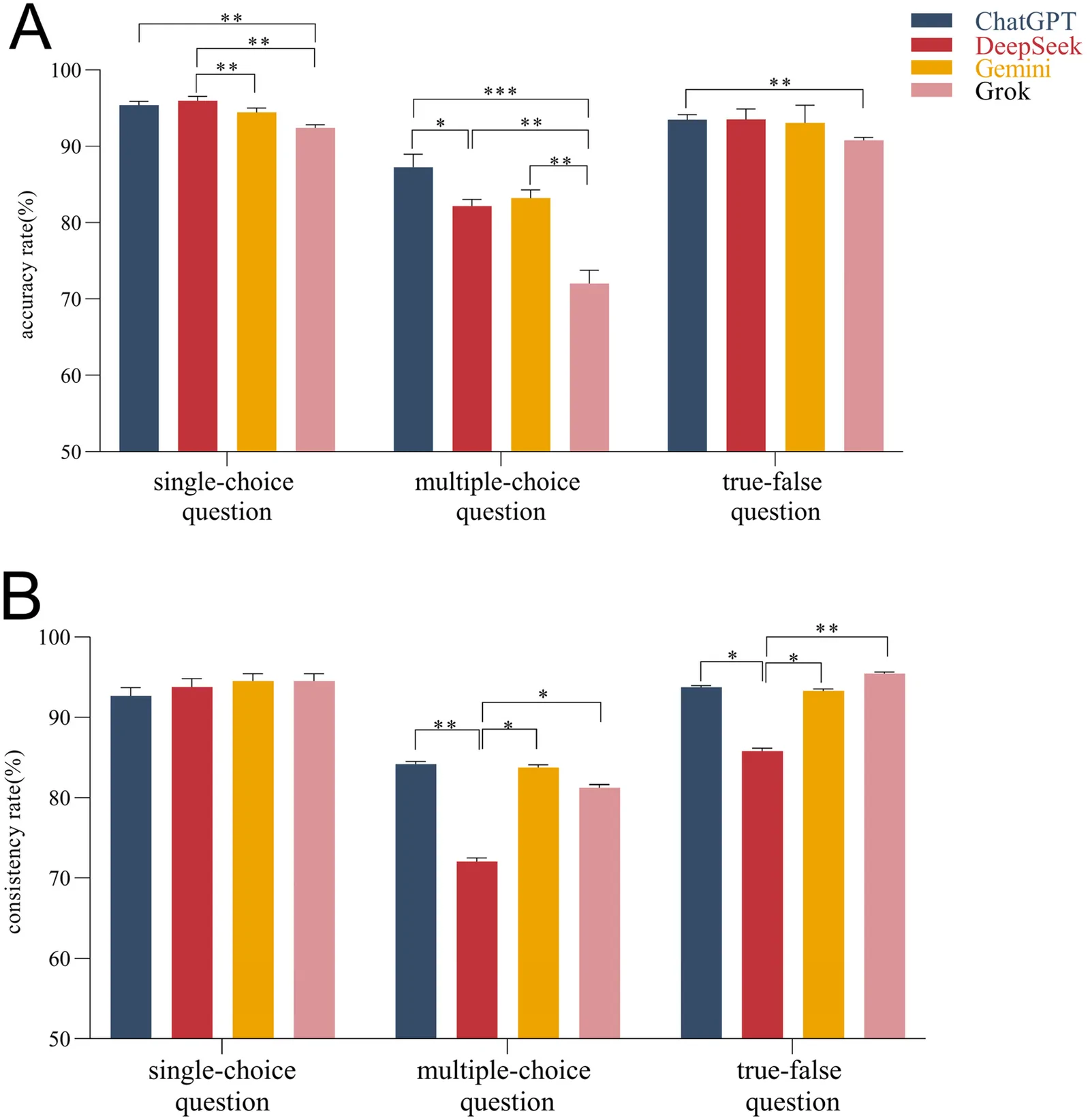
Accuracy and cross-session consistency of four large language models in medical knowledge testing. **(A)** Accuracy of ChatGPT-5.2 Thinking, DeepSeek-V3.2 chat, Gemini 3 Flash, and Grok 4.1 Fast on single-choice, multiple-choice, and true-false questions. **(B)** Cross-session consistency rates of the four models across the same three question types, based on five independent testing rounds. Error bars represent standard error. Statistical significance between groups is indicated as follows: *P < 0.05, **P < 0.01, ***P < 0.001.

Overall accuracy differed significantly among the four models across all three question types. For single-choice questions (F = 48.053, *P* < 0.001), DeepSeek-V3.2 chat (95.98% ± 0.60%) and ChatGPT-5.2 Thinking (95.40% ± 0.51%) both achieved significantly higher accuracy than Grok 4.1 Fast (92.42% ± 0.45%, *P* < 0.007); DeepSeek-V3.2 chat also outperformed Gemini 3 Flash (94.44% ± 0.58%, *P* = 0.006). For multiple-choice questions, between-model differences were more pronounced (F = 136.071, *P* < 0.001). ChatGPT-5.2 Thinking achieved the highest accuracy (87.28% ± 1.69%), significantly outperforming DeepSeek-V3.2 chat (82.18% ± 0.87%, *P* = 0.029) and Grok 4.1 Fast (72.06% ± 1.69%, *P* < 0.001); both DeepSeek-V3.2 chat and Gemini 3 Flash (83.22% ± 1.10%) also exceeded Grok 4.1 Fast (*P* ≤ 0.002). For true-false questions, differences were modest (F = 3.796, *P* = 0.04), with only ChatGPT-5.2 Thinking (93.46% ± 0.67%) significantly outperforming Grok 4.1 Fast (90.80% ± 0.40%, *P* = 0.007); all other pairwise comparisons were non-significant (Figure 1A).

#### 3.1.2 Cross-session consistency

For single-choice questions, consistency rates were uniformly high across all four models and did not differ significantly (Cochran's Q = 2.341, *P* = 0.505), ranging from 92.67% to 94.50%. For multiple-choice questions, between-group differences were significant (Cochran's Q = 16.841, *P* = 0.001). DeepSeek-V3.2 chat showed the lowest consistency rate (72.08% ± 0.45%), significantly below ChatGPT-5.2 Thinking (84.17% ± 0.37%, *P* = 0.006), Gemini 3 Flash (83.75% ± 0.37%, *P* = 0.012), and Grok 4.1 Fast (81.25% ± 0.39%, *P* = 0.016); no other pairwise differences reached significance. For true-false questions, DeepSeek-V3.2 chat (85.83% ± 0.35%) again showed significantly lower consistency than the other three models (93.33%—95.42%, all *P* < 0.05), with no significant differences among the remaining three (Cochran's Q = 23.259, *P* < 0.001) (Figure 1B).

### 3.2 Performance of the four LLMs in clinical case analysis

#### 3.2.1 Overall performance across eight evaluation dimensions

Significant differences among the four models were observed across all eight evaluation dimensions (F range 34.875–199.722, all *P* < 0.001). Bonferroni *post-hoc* comparisons showed that ChatGPT-5.2 Thinking scored significantly higher than the other three models in seven dimensions: accuracy and comprehensiveness of genetic risk estimation, accuracy and comprehensiveness of clinical phenotype prediction, accuracy and comprehensiveness of genetic counseling recommendations, and accuracy of test report interpretation (all *P* < 0.001). The sole exception was comprehensiveness of test report interpretation, where Grok 4.1 Fast achieved the highest score (4.93 ± 0.26), significantly exceeding all other models (all *P* < 0.001), while ChatGPT-5.2 Thinking and DeepSeek-V3.2 chat did not differ significantly (*P* = 0.126). DeepSeek-V3.2 chat and Gemini 3 Flash showed comparable performance in four dimensions: accuracy and comprehensiveness of genetic risk estimation, comprehensiveness of clinical phenotype prediction, and comprehensiveness of genetic counseling recommendations (all *P* > 0.05). Across all models, scores for accuracy of test report interpretation were relatively high (4.68–4.92), though pairwise differences remained significant in most comparisons (all *P* < 0.001), except between DeepSeek-V3.2 chat and Gemini 3 Flash (*P* = 0.126). Detailed scores are presented in Table 1.

**Table 1:** Performance of four LLMs across eight evaluation dimensions.

Dimension	ChatGPT	DeepSeek	Gemini	Grok	F value	P value
Report interpretation– accuracy	4.92 ± 0.33	4.71 ± 0.58	4.68 ± 0.60	4.84 ± 0.39	53.395	<0.001
Report interpretation– comprehensiveness	4.86 ± 0.40	4.81 ± 0.47	4.74 ± 0.51	4.93 ± 0.26	34.875	<0.001
Genetic risk—accuracy	4.83 ± 0.50	4.35 ± 1.15	4.34 ± 1.09	4.59 ± 0.85	68.776	<0.001
Genetic risk– comprehensiveness	4.78 ± 0.54	4.36 ± 1.15	4.33 ± 1.09	4.59 ± 0.83	58.029	<0.001
Phenotype prediction– accuracy	4.62 ± 0.63	3.79 ± 1.30	3.95 ± 1.25	4.17 ± 1.06	143.053	<0.001
Phenotype prediction– comprehensiveness	4.67 ± 0.63	3.92 ± 1.33	3.94 ± 1.24	4.30 ± 1.02	129.867	<0.001
Counseling recommendations– accuracy	4.60 ± 0.62	3.72 ± 0.99	4.01 ± 0.95	3.91 ± 0.80	199.722	<0.001
Counseling recommendations– comprehensiveness	4.56 ± 0.67	4.08 ± 0.87	4.03 ± 0.88	4.29 ± 0.76	100.488	<0.001

Data are presented as mean ± standard deviation. Overall between-group differences were assessed by one-way ANOVA; all *P* < 0.001.

#### 3.2.2 Overall performance across thalassemia subtypes

Significant between-model differences were observed across all six thalassemia subtype groups (F range: 5.32–97.17, all *P* ≤ 0.001). ChatGPT-5.2 Thinking achieved the highest overall score in every subtype (range: 4.37 ± 0.32 to 4.98 ± 0.08), and in most subgroups, its scores were significantly higher than the other three models. The relative ranking of the other three models varied by subtype. In mild *β*-thalassemia, ChatGPT-5.2 Thinking (4.98 ± 0.08) was significantly higher than DeepSeek-V3.2 chat (4.73 ± 0.18, *P* = 0.001), whereas no significant differences were found between ChatGPT-5.2 Thinking and Gemini 3 Flash (4.85 ± 0.23) or between ChatGPT-5.2 Thinking and Grok 4.1 Fast (4.84 ± 0.18). Pairwise comparisons among Gemini 3 Flash, Grok 4.1 Fast, and DeepSeek-V3.2 chat also revealed no significant differences (all *P* > 0.05). In moderate-to-severe *β*-thalassemia, ChatGPT-5.2 Thinking (4.89 ± 0.12) was significantly higher than Gemini 3 Flash (4.70 ± 0.18, *P* = 0.011) and Grok 4.1 Fast (4.65 ± 0.29, *P* < 0.001). However, no significant differences were observed between ChatGPT-5.2 Thinking and DeepSeek-V3.2 chat (4.77 ± 0.21), nor between DeepSeek-V3.2 chat and Gemini 3 Flash or Grok 4.1 Fast (all *P* > 0.05). In mild *α*-thalassemia, ChatGPT-5.2 Thinking (4.80 ± 0.62) performed significantly better than Grok 4.1 Fast (4.48 ± 0.42, *P* < 0.001). Grok 4.1 Fast, in turn, scored significantly higher than both Gemini 3 Flash (4.20 ± 0.73, *P* < 0.001) and DeepSeek-V3.2 chat (4.07 ± 0.54, *P* < 0.001). No significant difference was found between Gemini 3 Flash and DeepSeek-V3.2 chat (*P* = 0.307). In mild *α*-combined-*β*-thalassemia and moderate-to-severe *α*-thalassemia, pairwise comparisons among Gemini 3 Flash, Grok 4.1 Fast, and DeepSeek-V3.2 chat showed no significant differences (all *P* > 0.05), and all three models scored significantly lower than ChatGPT-5.2 Thinking (all *P* < 0.001). In moderate-to-severe *α*-combined-*β*-thalassemia, ChatGPT-5.2 Thinking (4.37 ± 0.32) and Grok 4.1 Fast (4.27 ± 0.47) did not differ significantly (*P* = 0.062). Both models scored significantly higher than Gemini 3 Flash (3.77 ± 0.65) and DeepSeek-V3.2 chat (3.83 ± 0.86) (all *P* < 0.001), whereas no significant difference was observed between Gemini 3 Flash and DeepSeek-V3.2 chat. Subtype scores are summarized in Table 2.

**Table 2:** Overall performance scores of four LLMs across six thalassemia subtypes.

Subtype	ChatGPT	Gemini	Grok	DeepSeek	F value	*P* value
Mild α-thalassemia	4.80 ± 0.62	4.20 ± 0.73	4.48 ± 0.42	4.07 ± 0.54	81.55	<0.001
Mild α-combined -*β*-thalassemia	4.88 ± 0.14	4.18 ± 0.83	4.15 ± 0.69	3.99 ± 0.62	97.17	<0.001
Mild *β*-thalassemia	4.98 ± 0.08	4.85 ± 0.23	4.84 ± 0.18	4.73 ± 0.18	5.32	0.001
Moderate-to-severe *α*-thalassemia	4.78 ± 0.36	4.35 ± 0.66	4.46 ± 0.75	4.38 ± 0.82	24.09	<0.001
Moderate-to-severe α-combined*-β*-thalassemia	4.37 ± 0.32	3.77 ± 0.65	4.27 ± 0.47	3.83 ± 0.86	88.62	<0.001
Moderate-to-severe *β*-thalassemia	4.89 ± 0.12	4.70 ± 0.18	4.65 ± 0.29	4.77 ± 0.21	7.45	<0.001

Data are presented as mean ± standard deviation. Overall between-group differences were assessed by one-way ANOVA; all *P* < 0.001.

### 3.3 Comparison between the four LLMs and human experts

**Figure 2:**
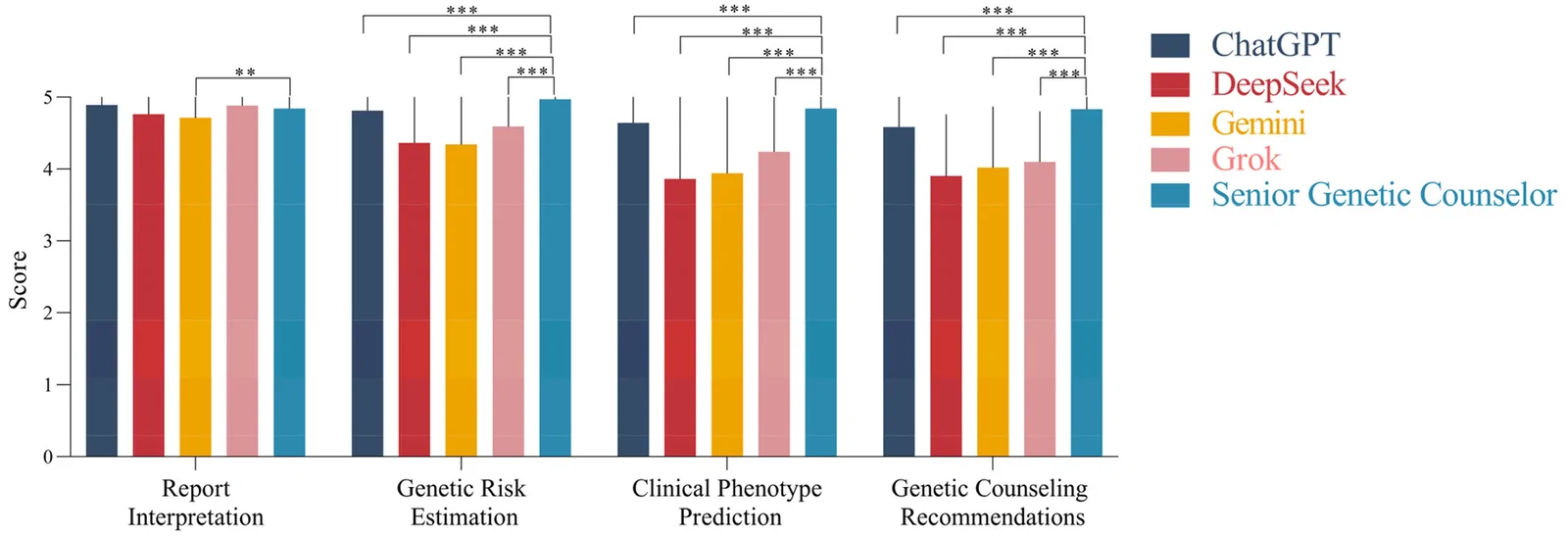
Performance scores of four large language models compared with senior genetic counselors across four counseling tasks. All scores are based on a 5-point scale rated by six senior genetic counseling experts. Error bars represent standard error. Statistical significance relative to human experts is indicated as follows: *P < 0.05, **P < 0.01, ***P < 0.001.

Significant between-group differences were observed across all four counseling tasks (*χ*^2^ = 40.51, 99.56, 152.28, and 217.11, respectively; all *P* < 0.001). For test report interpretation, ChatGPT-5.2 Thinking (4.89 ± 0.28), Grok 4.1 Fast (4.88 ± 0.21), and DeepSeek-V3.2 chat (4.76 ± 0.42) achieved scores comparable to human experts (4.84 ± 0.26), with no significant differences (all *P* > 0.05), whereas Gemini 3 Flash (4.71 ± 0.43) scored significantly lower (*P* = 0.004). For genetic risk estimation, clinical phenotype prediction, and genetic counseling recommendations, all four models scored significantly below human experts (all *P* < 0.001). ChatGPT-5.2 Thinking consistently achieved the highest scores among the four models across all tasks (genetic risk estimation: 4.81 ± 0.49; clinical phenotype prediction: 4.64 ± 0.58; genetic counseling recommendations: 4.58 ± 0.57) (Figure 2).

### 3.4 Subtype-stratified analysis for each counseling task

**Figure 3:**
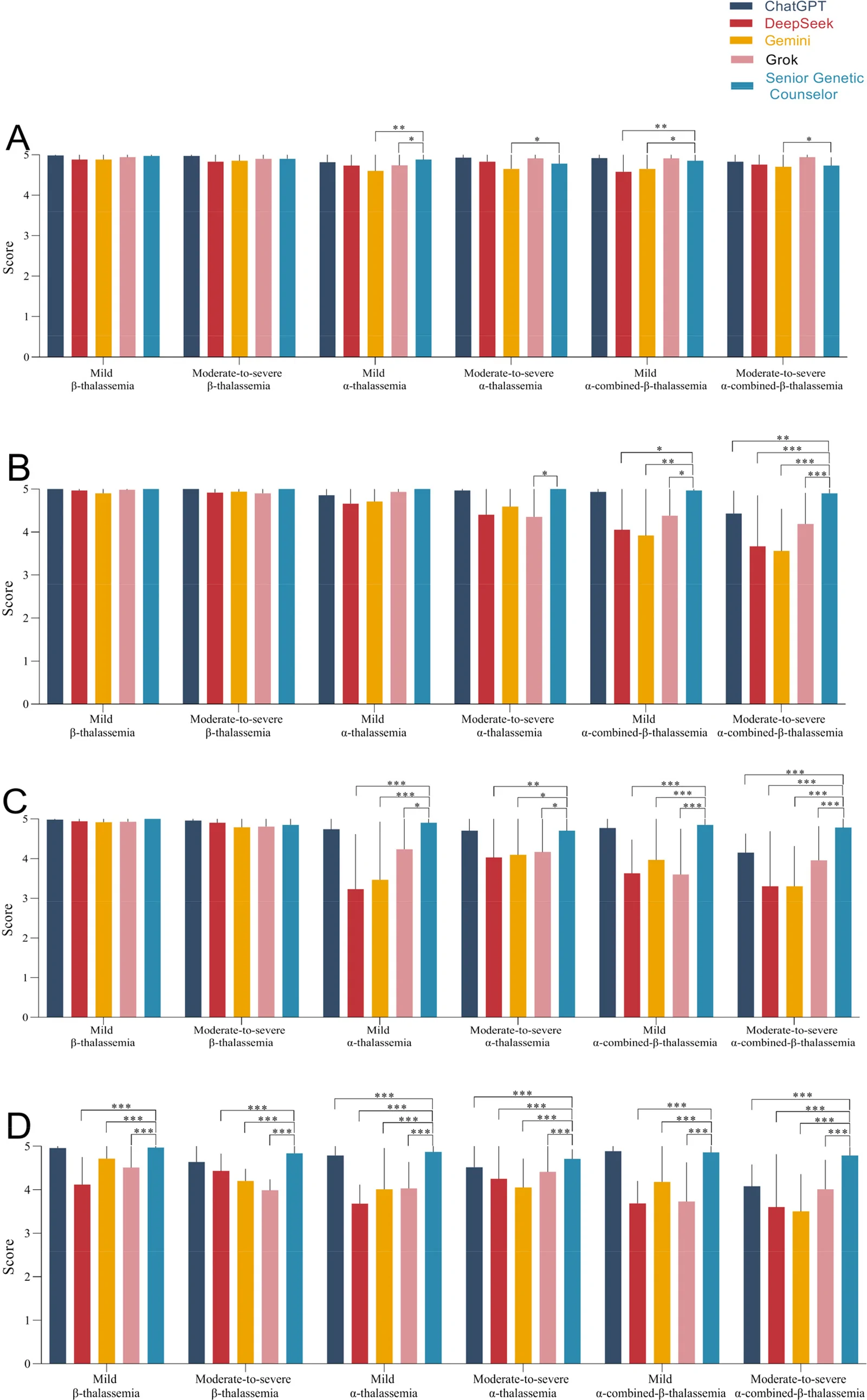
Performance scores of four large language models compared with senior genetic counselors across four counseling tasks and six thalassemia subtypes. (A–D) Subtype-stratified scores for each task: test report interpretation **(A)**, genetic risk estimation **(B)**, clinical phenotype prediction **(C)**, and genetic counseling recommendations **(D)** Subtypes are categorized as mild *α*-thalassemia, moderate-to-severe *α*-thalassemia, mild *β*-thalassemia, moderate-to-severe *β*-thalassemia, mild *α*-combined-*β*-thalassemia, and moderate-to-severe *α*-combined-*β*-thalassemia. All scores are based on a 5-point scale rated by six senior genetic counseling experts. Error bars represent standard error. Statistical significance relative to human experts is indicated as follows: *P < 0.05, **P < 0.01, ***P < 0.001.

For test report interpretation, ChatGPT-5.2 Thinking matched human experts across all six subtypes (*P* = 0.302–0.842). Grok 4.1 Fast and DeepSeek-V3.2 chat fell significantly below human experts only in mild *α*-thalassemia (*P* = 0.015) and mild *α*-combined-*β*-thalassemia (*P* = 0.003), respectively. Gemini 3 Flash was significantly inferior to human experts in four subtypes: mild *α*-thalassemia, mild *α*-combined-*β*-thalassemia, moderate-to-severe *α*-thalassemia, and moderate-to-severe *α*-combined-*β*-thalassemia (*P* = 0.002–0.028). No model differed significantly from human experts in either *β*-thalassemia subtype (all *P* > 0.05) (Figure 3A).

For genetic risk estimation, performance gaps relative to human experts were most pronounced in *α*-combined-*β*-thalassemia subtypes. In mild *α*-combined-*β*-thalassemia, ChatGPT-5.2 Thinking (4.93 ± 0.16) did not differ significantly from human experts (*P* = 0.744), whereas the other three models scored significantly lower (all *P* < 0.05). In moderate-to-severe *α*-combined-*β*-thalassemia, all four models fell significantly below human experts (ChatGPT-5.2 Thinking 4.43 ± 0.53, DeepSeek-V3.2 chat 3.67 ± 1.18, Gemini 3 Flash 3.56 ± 0.98, Grok 4.1 Fast 4.19 ± 0.72; all *P* < 0.05). In mild *α*-thalassemia, mild *β*-thalassemia, and moderate-to-severe *β*-thalassemia, no model differed significantly from human experts (all *P* > 0.05) (Figure 3B).

For clinical phenotype prediction, no model differed significantly from human experts in either *β*-thalassemia subtype (all *P* > 0.05), whereas all models scored significantly below human experts in moderate-to-severe *α*-combined-*β*-thalassemia (all *P* < 0.001). ChatGPT-5.2 Thinking matched human experts in all remaining subtypes (all *P* > 0.05). Gemini 3 Flash, Grok 4.1 Fast, and DeepSeek-V3.2 chat were significantly lower than human experts in mild *α*-thalassemia, moderate-to-severe *α*-thalassemia and mild *α*-combined-*β*-thalassemia (*P* = 0.026 to *P* < 0.001) (Figure 3C).

For genetic counseling recommendations, the gap between models and human experts was largest among all four tasks. ChatGPT-5.2 Thinking did not differ significantly from human experts only in mild *β*-thalassemia (*P* = 0.260), moderate-to-severe *β*-thalassemia (*P* = 0.083), and mild α-combined-*β*-thalassemia (*P* = 0.167); in all other subtypes, all four models scored significantly below human experts (all *P* < 0.001). The lowest scores across all models were observed in moderate-to-severe α-combined-*β*-thalassemia (ChatGPT-5.2 Thinking 4.08 ± 0.50, Gemini 3 Flash 3.50 ± 0.86, DeepSeek-V3.2 chat 3.60 ± 1.22, Grok 4.1 Fast 4.01 ± 0.68), representing the largest deviation from human experts (4.79 ± 0.19). DeepSeek-V3.2 chat scored below 4.0 in mild *α*-thalassemia (3.68 ± 0.44), mild *α*-combined-*β*-thalassemia (3.69 ± 0.51), and moderate-to-severe α-combined-*β*-thalassemia (3.60 ± 1.22), the widest gap among the four models (Figure 3D).

### 3.5 Distribution of severe errors

A total of 145 severe errors were identified across all models and subtypes. In contrast, no severe errors were detected in the answer outputs from the two senior genetic counselors serving as the human reference standard, indicating high consistency of diagnostic and counseling standards for the included cases among expert practitioners.

**Figure 4:**
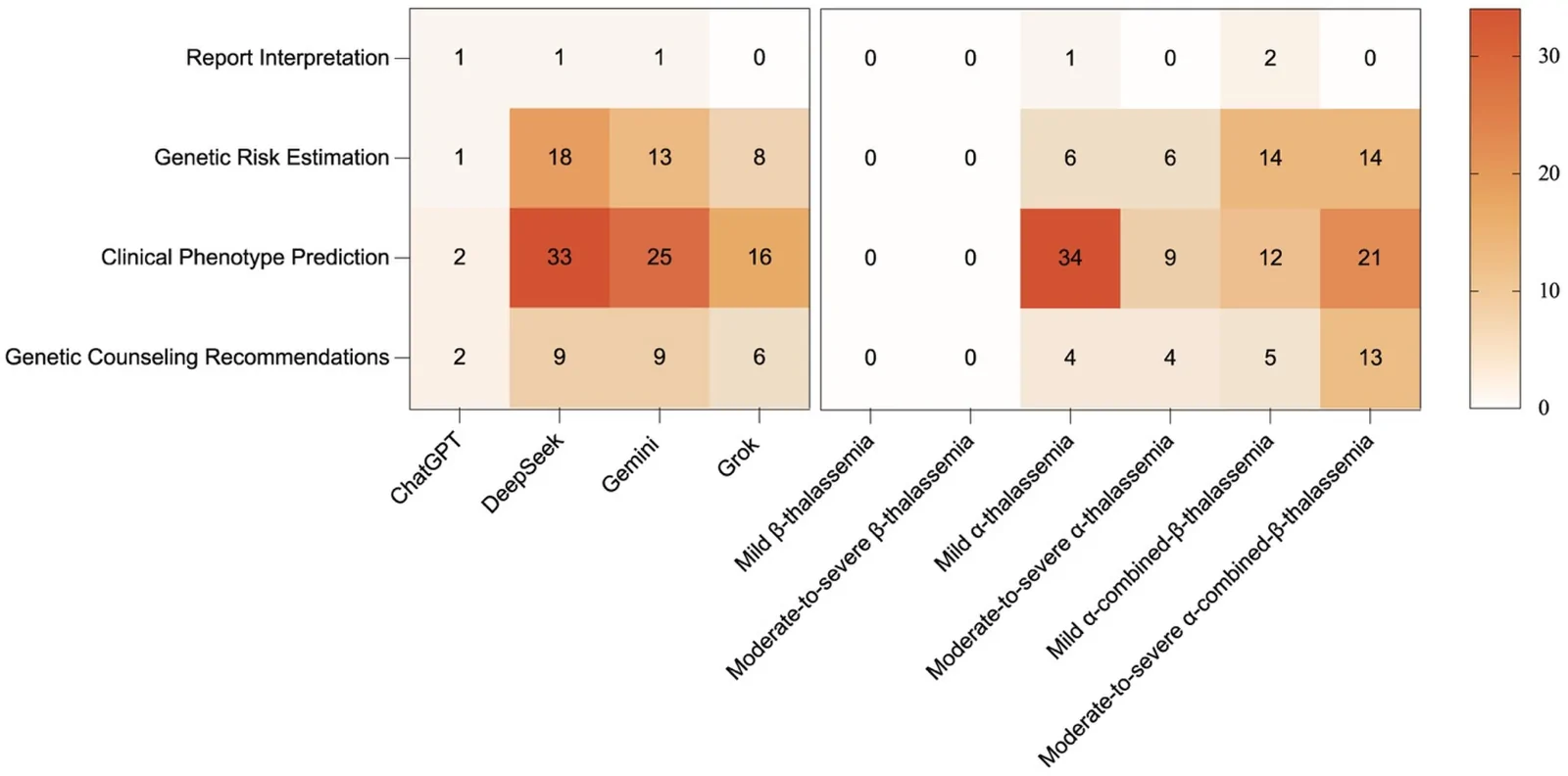
Distribution of severe errors across evaluation dimensions,models,and thalassemia subtypes. Each cell displays the number of severe errors recorded in each evaluation dimension (rows). The left panel shows the distribution by model (ChatGPT-5.2 Thinking, DeepSeek-V3.2 chat, Gemini 3 Flash, and Grok 4.1 Fast); the right panel shows the distribution by thalassemia subtype. Color intensity reflects error count, with deeper orange red indicating a higher number of severe errors. No severe errors were observed in either *β*-thalassemia subtype across any model or evaluation dimension.

By task, clinical phenotype prediction accounted for the largest share (76/145, 52.4%), followed by genetic risk estimation (40/145, 27.6%), genetic counseling recommendations (26/145, 17.9%), and test report interpretation (3/145, 2.1%). By model, ChatGPT-5.2 Thinking made the fewest severe errors (*n* = 6), followed by Grok 4.1 Fast (*n* = 30), Gemini 3 Flash (*n* = 48), and DeepSeek-V3.2 chat (*n* = 61). By subtype, severe errors were concentrated in moderate-to-severe *α*-combined-*β*-thalassemia (*n* = 48), mild *α*-thalassemia (*n* = 45), and mild *α*-combined-*β*-thalassemia (*n* = 33). No severe errors were observed in any *β*-thalassemia subtype (Figure 4).

## 4 Discussion

Genetic counseling resources for thalassemia remain chronically imbalanced relative to demand in high-prevalence regions. The present study systematically examined the performance and safety boundaries of four general-purpose LLMs in this setting, using a dual-track framework that combined standardized knowledge testing with real-world clinical case analysis and human expert benchmarking. Across both tracks, a consistent task-stratification pattern emerged: LLMs performed comparably to human experts in structured report interpretation but fell substantially short in probabilistic reasoning tasks, with all severe errors confined to *α*-related subtypes.

This stratification closely tracked the cognitive demands of each counseling step. In tasks with lower reasoning requirements, such as knowledge retrieval and structured test report interpretation, all four models performed well, with scores approaching those of senior genetic counselors. This task-dependent performance gradient is consistent with findings from a recent comparative study of three LLMs, which similarly demonstrated that AI systems achieve robust performance in low-complexity, low-reasoning clinical tasks despite focusing on a distinct disease context (25). As task complexity increased through genetic risk inference, clinical phenotype prediction, and synthesis of counseling recommendations, performance declined markedly and inter-model variability widened. This pattern is consistent with prior observations that high accuracy on medical knowledge assessments does not reliably predict clinical decision-making performance (15, 21, 26), and it underscores the distinction between information retrieval and multi-step probabilistic reasoning in the context of genetic counseling.

Among the four models, ChatGPT-5.2 Thinking demonstrated consistent advantages across most dimensions and subtypes, while DeepSeek-V3.2 chat showed relative weaknesses in cross-session consistency and severe error control. This degree of inter-model heterogeneity has practical implications: in high-stakes clinical settings, different LLMs cannot be treated as functionally equivalent, and model selection requires empirical evaluation rather than assumption of comparable capability (27, 28).

Subtype-level analysis helps clarify the mechanistic basis of these performance differences. All models performed stably in *β*-thalassemia, with no severe errors recorded, whereas performance declined substantially in *α*-thalassemia and α-combined-*β*-thalassemia, where all 145 severe errors were concentrated. This divergence is unlikely to reflect a general deficit in thalassemia knowledge. *β*-thalassemia follows a relatively classical inheritance pattern with standardized clinical management pathways, conditions under which LLMs can apply consistent reasoning frameworks derived from training data. *α*-related thalassemia presents a more demanding inferential challenge: the *α*-globin gene cluster is subject to complex structural variation, phenotypic outcomes differ substantially between deletional and non-deletional variants, copy number changes introduce additional heterogeneity, and polygenic modifier effects are not captured by routine testing. Furthermore, *α*-globin gene alterations can modify the clinical expression of co-existing *β*-thalassemia mutations, and assessing the severity of hemoglobin H disease requires integrating multiple clinical parameters in a non-linear fashion (9–11, 29). When the inferential task involves incomplete information and context-dependent rules of this complexity, model error rates rise substantially. More broadly, these findings suggest that the performance ceiling of general-purpose LLMs in clinical genetics is determined not by domain knowledge coverage but by the structural complexity of the inferential task itself.

The concentration of severe errors in clinical phenotype prediction and genetic risk estimation carries direct clinical significance. These two tasks determine whether invasive prenatal diagnosis is indicated and directly inform reproductive decisions in high-risk families; errors in either domain can produce irreversible consequences (19, 30). The present findings therefore do not support pathways in which general-purpose LLMs generate final diagnostic or counseling outputs without expert oversight. A more viable approach is a layered, modular workflow in which task allocation is matched to model capability. Preparatory and low-complexity tasks, including pre-counseling patient education, triage of routine questions, and initial explanation of structured test reports, are appropriate targets for LLM assistance and could meaningfully reduce resource pressure in high-prevalence regions. Tasks requiring precise genetic probability calculation, interpretation of complex genotype combinations, multi-parameter fetal prognosis assessment, and formulation of final reproductive recommendations should be reserved for rule-based engines, disease-specific knowledge bases, and human genetic counselors working in combination. Defining this boundary between text-transfer tasks suitable for LLM support and complex clinical decisions requiring human oversight reflects a broadly applicable principle for responsible medical AI implementation (31–33).

Several limitations warrant consideration. The single-center design, region-specific mutation spectrum, and locally derived knowledge test set limit generalizability, while ceiling effects in report interpretation scores may have reduced discriminative sensitivity. Strict scorer blinding to model identity was not feasible, as model-specific formatting identifiers in exported PDFs and screenshots prevented complete masking of model source without compromising content integrity; although we mitigated potential bias through randomized presentation, six-expert independent scoring, and a standardized rubric, fully blinded evaluation should be pursued in future studies using plain-text output templates. The human expert comparator group was small, and we assessed only single-turn, unaugmented outputs without retrieval-augmented generation, multimodal input, or agentic architectures (34). We evaluated only commercial cloud-based LLMs, excluding locally deployable offline models, as the substantial computational infrastructure required for local deployment was not available at our institution during the study period; moreover, our primary objective was to establish a performance baseline for the most advanced models that clinicians might realistically consider adopting in high-prevalence regions. Nonetheless, we acknowledge the privacy implications of this exclusion, and future work should prioritize offline model adaptation as local infrastructure and open-source capabilities continue to evolve. Lastly, patient outcomes were not tracked, so our findings represent a performance baseline in a controlled setting; future studies should integrate disease-specific retrieval-augmented generation, rule-based calculation modules, and structured expert review into clinician-supervised workflows to compensate for the probabilistic reasoning deficits identified here.

## 5 Conclusions

LLMs demonstrated reliable performance in thalassemia knowledge retrieval and structured test report interpretation, approaching human expert levels in these domains, but fell substantially short in genetic risk estimation, clinical phenotype prediction, and counseling recommendations, with all severe errors confined to *α*-related subtypes. LLMs may serve as auxiliary tools for low-complexity counseling tasks but should not be used independently in high-stakes decisions. Professional review remains essential, and future work should focus on clinician-supervised workflows that integrate rule-based calculation and disease-specific retrieval-augmented generation.

## Data Availability

The datasets presented in this study can be found in online repositories. The names of the repository/repositories and accession number(s) can be found in the article/Supplementary Material.
