# Supplementary material for: Performance and limitations of four large language models in genetic counseling for thalassemia
Source: Front Digit Health. 2026 Aug 11;8:1902480. doi: 10.3389/fdgth.2026.1902480 (PMC13464380; doi:10.3389/fdgth.2026.1902480)
Supplement: Supplementary file 1 [file Datasheet1.docx]

1. 地中海贫血的类型包括（ ）

A. α-地中海贫血	B. β-地中海贫血	C. δ-地中海贫血	D. γ-地中海贫血

2. 重型地中海贫血可能导致的并发症有（ ）

A. 心力衰竭	B. 生长发育迟缓	C. 铁过载	D. 感染

3. 地中海贫血的诊断依据包括（ ）

A. 家族史	B. 临床表现	C. 实验室检查	D. 影像学检查

4. 对于地中海贫血患者，输血治疗的目的是（ ）

A. 改善贫血症状	B. 提高生活质量	C. 促进生长发育	D. 防止并发症

5. 缺铁性贫血患者可能出现的特殊表现有：（）

A. 异食癖	B. 匙状甲	C. 吞咽困难	D. 毛发干枯

6. 地中海贫血患者在饮食上应注意（ ）

A. 避免过量摄入含铁食物	B. 多吃富含维生素C的食物	C. 保证营养均衡	D. 多吃高蛋白食物

7. 地中海贫血的治疗方法有（ ）

A. 输血治疗	B. 祛铁治疗	C. 造血干细胞移植	D. 基因治疗

8. 轻型地中海贫血患者在日常生活中需要注意（ ）

A. 避免劳累	B. 预防感染	C. 定期复查	D. 无需特殊注意

9. 进行地中海贫血基因检测的意义在于（ ）

A. 明确诊断	B. 指导生育	C. 评估病情	D. 制定治疗方案

10. 地中海贫血患者可能出现的血液学异常有（ ）

A. 红细胞形态改变	B. 血红蛋白降低	C. 红细胞数量减少	D. 网织红细胞增多

11. 以下哪些属于贫血的临床表现？（）

A. 皮肤苍白	B. 头晕、乏力	C. 心悸、气促	D. 耳鸣、眼花

12. 下列需要做地贫产前诊断的情况有哪些？（）

A. 同为轻型β地贫夫妇结婚怀孕	B. 夫妇双方只有一方为轻型地贫患者	C. 正常人与β轻型地贫基因携带者结婚	D. 同型α地贫夫妇结婚怀孕

13. 产前诊断包括什么方式?（）

A. 绒毛穿刺术:穿刺时间为孕10-14周	B. 羊水穿刺术:穿刺时间在孕16周后	C. 脐带血穿刺术:多用于产前超声筛查发现的重型α地贫胎儿（hb Bart′s水肿胎）的确认，穿刺时间多在孕20周后

14. 血常规用作地贫初筛的缺点有哪些（）?

A. 无法区分缺铁性贫血和地中海贫血	B. 静止型α地贫及部分β+地贫其血液学表型可能为正常，只看血常规可能造成漏检，血常规地贫漏检率高达50%	C. 任何一次MCV/MCH值下降都要引起重视，都要建议做地贫基因	D. 单看血常规，无法提示受检者为何种型别的地贫

15. 地中海贫血患者可能出现的并发症包括：（）

A. 铁过载	B. 心力衰竭	C. 肝功能异常	D. 感染

16. 地中海贫血的诊断方法包括：（）

A. 血常规检查	B. 血红蛋白电泳	C. 分子生物学检测	D. 骨髓活检

17. 地中海贫血的治疗措施包括：（）

A. 输血治疗	B. 铁剂治疗	C. 脾切除术	D. 基因治疗

18. 地中海贫血的预防措施包括：（）

A. 遗传咨询	B. 产前诊断	C. 新生儿筛查	D. 避免近亲结婚

19. 地中海贫血的临床症状可能包括（）

A. 贫血	B. 黄疸	C. 肝脾肿大	D. 发育迟缓

20. 地中海贫血的高发人群包括（）

A. 地中海沿岸居民	B. 东南亚地区居民	C. 非洲部分地区居民	D. 中国南方居民

21. 地中海贫血的诊断需综合考虑（）

A. 家族史	B. 临床表现	C. 实验室检查	D. 影像学检查

22. 地中海贫血患者可能并发的疾病（）

A. 含铁血黄素沉着症	B. 胆石症	C. 心力衰竭	D. 感染

23. 地中海贫血患者日常生活中需注意（）

A. 避免感染	B. 合理饮食	C. 适度运动	D. 定期复查

24. 地中海贫血患者在输血治疗时可能面临的问题有（ ）

A. 输血反应	B. 铁过载	C. 传染病传播风险	D. 血液供应不足

25. 地中海贫血患者可能出现的血液学改变有（ ）

A. 红细胞大小不均	B. 靶形红细胞增多	C. 血红蛋白降低	D. 白细胞计数升高

26. 导致地中海贫血的基因突变类型有（ ）

A. 缺失突变	B. 点突变	C. 插入突变	D. 重排突变

27. 重型地中海贫血患者的治疗方法包括（ ）

A. 定期输血	B. 去铁治疗	C. 造血干细胞移植	D. 药物治疗

28. 轻型地中海贫血患者可能出现的情况有（ ）

A. 无明显症状	B. 轻度贫血	C. 不需要特殊治疗	D. 发展为重型

29. 以下哪些因素可能影响地中海贫血患者的病情（ ）

A. 感染	B. 营养状况	C. 妊娠	D. 情绪

30. 地中海贫血与缺铁性贫血的鉴别要点有（ ）

A. 血清铁	B. 铁蛋白	C. 血红蛋白电泳	D. 红细胞形态

31. 地中海贫血患者在日常生活中需要注意（ ）

A. 避免感染	B. 适当休息	C. 合理饮食	D. 定期复查

32. 目前针对地中海贫血的基因治疗策略包括（ ）

A. 基因编辑	B. 基因替代	C. 基因调控	D. 基因增强

33. 地中海贫血的临床表现包括：（）

A. 贫血	B. 黄疸	C. 肝脾肿大	D. 骨骼改变

34. 关于地中海贫血的实验室检查，以下正确的是：（）

A. 血常规可见红细胞平均体积降低	B. 血红蛋白电泳可出现异常区带	C. 基因检测可明确诊断	D. 血清铁和铁蛋白通常降低

35. 重型地中海贫血患者可能出现的并发症有：（）

A. 铁过载	B. 感染	C. 生长发育迟缓	D. 心力衰竭

36. 地中海贫血的预防措施包括：（）

A. 婚前筛查	B. 孕前基因诊断	C. 孕期筛查	D. 避免近亲结婚

37. 以下属于α-地中海贫血类型的有：（）

A. 静止型	B. 轻型	C. 中间型	D. 重型

38. 地中海贫血患者的饮食建议包括：（）

A. 均衡饮食	B. 避免摄入过多含铁食物	C. 多吃富含维生素C的食物	D. 多吃高蛋白食物

39. 地中海贫血基因携带者可能的情况有：（）

A. 无明显症状	B. 血常规可能正常	C. 血红蛋白电泳可无异常	D. 有一定的遗传风险

40. 地中海贫血的治疗方法包括：（）

A. 输血治疗	B. 去铁治疗	C. 脾切除	D. 造血干细胞移植

41. 以下哪些因素可能影响地中海贫血患者的病情：（）

A. 感染	B. 劳累	C. 情绪波动	D. 药物副作用

42. 地中海贫血的遗传特点包括：（）

A. 常染色体隐性遗传	B. 父母双方均为携带者时，子女有患病风险	C. 遗传与性别无关	D. 携带者可能无临床症状

43. 关于地中海贫血与缺铁性贫血的区别，正确的是：（）

A. 发病机制不同	B. 血清铁和铁蛋白水平不同	C. 血常规表现不同	D. 治疗方法不同

44. 地中海贫血患者进行脾切除的目的有：（）

A. 减少红细胞破坏	B. 减轻贫血症状	C. 减少输血次数	D. 提高免疫力

45. 地中海贫血可能导致的骨骼改变有：（）

A. 头颅变大	B. 额部隆起	C. 鼻梁塌陷	D. 上颌前突

46. 地中海贫血患者在日常生活中需要注意：（）

A. 避免剧烈运动	B. 预防感染	C. 定期复查	D. 保持良好的作息

47. 以下哪些情况可能提示地中海贫血（ ）

A. 小细胞低色素性贫血，铁剂治疗无效	B. 有地中海贫血家族史	C. 黄疸伴有脾大	D. 反复贫血发作

48. 以下关于地中海贫血的说法正确的是（ ）

A. 可分为α-地中海贫血和β-地中海贫血	B. 不同类型的地中海贫血临床表现有所差异	C. 基因缺陷是其发病的根本原因	D. 是一种单基因遗传病

49. 地中海贫血患者输血治疗的目的包括（ ）

A. 改善贫血症状	B. 抑制骨髓过度造血	C. 减少肠道铁吸收	D. 延长患者生存期

50. 对于地中海贫血患者的婚育指导，正确的是（ ）

A. 婚前进行地中海贫血基因检测	B. 双方均为地中海贫血基因携带者应进行遗传咨询	C. 避免近亲结婚	D. 重型地中海贫血患者不建议生育

51. 患者处于下列哪些情况时可诊断为铁过载（）

A. 接受输血治疗10-20次	B. TS>50%	C. SF>1000μg∕l	D. LIC>12mgFe/gDW

52. 瑞益格®的患者人群有哪些（）

A. 地中海贫血铁过载	B. MDS（骨髓增生异常综合征）铁过载	C. AA（再生障碍性贫血）铁过载	D. 贫血

53. 瑞益格®对比去铁胺和去铁酮的优势有哪些（）

A. 瑞益格®口服，一天1次，患者依从性好	B. 去铁胺需要持续皮下注射，每周＞5天，使用方法繁琐	C. 瑞益格®影响患者生活质量的不良反应少，大多数患者耐受性良好	D. 祛铁酮关节痛、中性粒细胞减少等不良反应风险较高

54. 瑞益格®对比地拉罗司分散片的优势有哪些（）

A. 生物利用度更高，可减少给药剂量，减轻患者负担	B. 更小的给药剂量，更低杂质摄入，安全性更高	C. 去除分散片中的S1S，进一步减少胃刺激	D. 瑞益格®地拉罗司片载药量高，服用时食物影响小，餐前餐后均可服用，更方便

55. 可以用于胎儿地中海贫血产前诊断的材料有（	）

A、脐血 B、绒毛  C、羊水 D、孕妇外周血

56. 下列关于地中海贫血的描述，正确的有：（）

A. 有家族遗传倾向	B. 可分为α和β地中海贫血	C. 轻型患者可能无症状	D. 重型患者需要长期输血治疗

57. 以下哪些人群需要进行地中海贫血筛查？（ ）

A. 新婚夫妇	B. 有地贫家族史者	C. 贫血患者	D. 儿童保健体检时

58. 地贫患者的实验室检查指标可能有（ ）

A. 血红蛋白降低	B. 平均红细胞体积降低	C. 红细胞渗透脆性降低	D. 血清铁蛋白升高

59. 对于地中海贫血患者的治疗，以下正确的是（ ）

A. 重型患者需要长期输血支持	B. 铁螯合剂可用于减少铁过载	C. 脾切除可改善部分患者的贫血症状	D. 基因治疗目前已广泛应用

60. 地中海贫血的临床表现与（ ）有关

A. 基因缺陷类型	B. 贫血严重程度	C. 患者年龄	D. 患者性别

61. 以下关于地中海贫血的说法正确的是（ ）

A. 是全球分布最广的单基因遗传病之一	B. 在我国南方地区高发	C. 可导致不同程度的贫血	D. 无法进行产前诊断

62. 关于产前诊断伦理监督和权益保护，包括(	)

A:实施产前诊断的医疗机构必须建立产前诊断伦理委员会	B:产前诊断伦理委员会由医学伦理学、遗传学、心理学、法学、管理学等相关领域的专家和群众代表等组成	C:伦理委员会对本单位的产前诊断中的相关伦理问题进行监督	D:伦理委员会对实验中遇到的伦理问题进行审查，登记和商议，以维护当事人的正当权益

63. 产前诊断伦理指导要求（	）

A：当事人的遗传信息、产前诊断结果，是否选择终止妊娠等均属于个人隐私	B：医务人员应当尊重当事人的个人隐私，有责任为其保守秘密	C：应当避免由于检查结果给当事人及亲属带来不良后果	D：如检查结果涉及可影响当事人亲属发病风险的遗传信息，由医务人员决定是否告诉当事人有关亲属

64. 产前诊断伦理指导原则要求___（）

A:医务人员应综合考虑孕妇及家庭的负面情况，尽可能采用损伤小、效果好，花费少的诊断方法	B:严格掌握产前诊断的适应症和进行产前诊断的条件	C:禁止非医学目的的产前性别诊断	D:不需性别选择的产前诊断，可以报告胎儿性别

65. 《广西壮族自治区地中海贫血防治计划》工作目标和工作任务分别为（	）

A:到2015年全区重型地贫患儿出生率比2009年下降50%以上，新生儿出生缺陷发生率明显下降	B:对相关医务人员进行地贫防治知识宣传，教育和培训，使相关医务人员地贫防治知识知晓率达95%以上	C: 到2015年，在校青少年地贫防治知识知晓率达到90%以上，校外青年及群众达到80%以上	D: 到2015年，婚检，孕前保健发现的地贫高危重点人群防治知识知晓率达95%以上

66. 产前诊断伦理指导原则要求（	）

A:保证当事人的自主知情同意权 B：医务人员应在当事人签署书面知情同意书后方可实施产前诊断或产前筛查 C: 医务人员应将产前诊断的结果告知当事人 D: 是否选择终止妊娠由医务人员作出决定

67. 医师在产前诊断时发现以下情形之一时，应向孕妇及家属说明情况，并提出终止妊娠的医学意见（ ）

A、胎儿患有严重遗传疾病的	B、因患严重疾病，继续妊娠可能危及孕妇生命安全的	C、胎儿患有严重缺陷的	D、因患严重疾病，继续妊娠严重危害孕妇健康的

68. 孕妇有下列情形之一的，经治医师应当建议其进行产前诊断（ ）

A、年龄超过35周岁的	B、胎儿发育异常或者胎儿有可疑畸形的	C、孕早期时接触过可能导致胎儿先天缺陷的物质的	D、有遗传病家族史或者曾经分娩过先天性严重缺陷婴儿的

69. 自治区卫生计生行政部门依法开展（ ）

A、产前诊断机构和人员许可	B、组织产前筛查和产前诊断专业技术人员系统培训	C、对开展产前筛查、产前诊断技术服务机构的执业情况进行监督、检查	D、支持与产前筛查、产前诊断技术相关的科学研究，组织推广相关的适宜技术

70. 产前诊断报告，应（	）

A、由2名经资格认定的具有高级职称的执业医师签发	B、由遗传咨询室医师提供医学咨询	C、由医院产前诊断办公室工作人员签字	D、报告应当一式二份，当事人一份，医疗保健机构存档一份

71. 下列哪些情况可能导致缺铁性贫血（）

A. 长期素食	B. 胃肠道疾病影响铁吸收	C. 妇女月经量过多	D. 儿童生长发育过快

72. 以下关于母婴保健医学技术鉴定的说法，正确的是（ ）

A、对婚前医学检查或产前诊断结果有异议的，可申请进行母婴保健医学技术鉴定	B、母婴保健医学技术鉴定委员会分为省、市两级	C、母婴保健医学技术鉴定组织为母婴保健医学技术鉴定委员会	D、医学技术鉴定时，与当事人有利害关系、可能影响公正鉴定的人员应回避

73. 申请开展产前诊断技术的医疗保健机构应当向所在地省级卫生行政部门提交下列文件（	）

A、医疗机构执业许可证副本	B、开展产前诊断技术的母婴保健技术服务执业许可申请文件	C、开展产前诊断技术的规章制度	D、拟开展产前诊断技术的人员配备、设备和技术条件情况

74. 对父母进行染色体检查的适应症是（	）

A、生过智力低下、多发畸形患儿	B、有自然流产和不育史	C、高龄孕妇	D、生过先天性代谢病患儿

75. 产前诊断伦理指导原则包括（	）

A.尊重自主，知情同意	B.趋利避害，有利母儿	C.保守秘密，尊重隐私	D遵守法规，社会公益

76. 《母婴保健法》规定孕产期保健包括以下哪些内容（	）

A．母婴保健指导B、孕妇、产妇保健	C．胎儿保健	D．婚前保健

77. 各级政府应当（ ）

A．采取措施,加强母婴保健事业发展 B．促进母婴保健事业发展	C．积极防治地方性高发疾病	D．将母婴保健事业纳入经济和社会发展规划

78. 未取得相关资质,而从事母婴专业技术服务的人员,可给予何种处罚（ ）

A．责令停止	B．罚款处罚	C．警告	D．由卫生行政部门取消其执业资格

79. 婚前卫生指导包括下列哪些内容（	）

A．生育知识	B．性卫生知识	C．遗传病知识 D．传染病知识

80. 发现孕妇有下列情形之一的,应进行产前诊断（ ）

A．孕早期接触过可能导致胎儿先天缺陷的物质 B．孕妇年龄超过30周岁 C．胎儿发育异常或胎儿可能有畸形	D．曾分娩过先天性严重缺陷的婴儿

81. Down综合征遗传学类型有（	）

A．游离型	B．缺失型	C．嵌合型	D．易位型

82. 染色体的次溢痕包括（	）

A．着丝粒部位	B．随体柄部位	C．长臂的缩窄部位 D．短臂的缩窄部位

83. 染色体重复发生的原因可为（	）

A．同源染色体发生不等交换	B．染色单体之间发生不等交换	C．染色体片段插入	D．核内复制

84. 下列哪些不属于染色体结构畸变（ ）

A.13-三体综合症 B.18-三体综合症 C.21-三体综合征 D.5p-综合症

85. 染色体数目异常形成的可能原因是（ ）

A．染色体断裂	B．染色体倒位	C．染色体丢失	D．染色体不分离

86. 下列有必要做染色体检查的是（	）

A．习惯性流产者	B．感冒患者	C．智力低下者	D．不孕者

87. 下列哪些染色体为近端着丝粒染色体（	）

A．第21～22号染色体	B．第13～15号染色体	C．第6～12号染色体	D．第16～18号染色体

88. 染色体检查的指征有（ ）

A．发育障碍	B．智力低下	C．反复流产	D．免疫力低下

89. 三倍体的形成机理可能是（ ）

A．双雌受精	B．双雄受精	C．染色体不分离	D．核内有丝分裂

90. 染色体病的临床上特点是（ ）

A．先天性多发畸形	B．性发育落后	C．特殊肤纹	D．智力障碍

91. 染色体畸变发生的原因包括（ ）

A．物理因素	B．化学因素	C．生物因素	D．遗传因素

92. 最为常见的三体型病变染色体编号为（ ）

A．21	B．13	C．15	D．18

93. 结构异染色质包括以下几个部位（	）

A．着丝粒区	B．端粒区	C．次溢痕区	D．Y染色体长臂远端2/3区段

94. 染色体不分离可以发生在（）

A．姐妹染色单体之间	B．同源染色体之间	C．有丝分裂过程中	D．减数分裂过程中

95. Turner综合征(性腺发育不全)的异常核型包括（	）

A、45, X/46,XX	B、45, X	C、46, XXq	D、46, XXp

96. 人类染色体的类型包括（ ）

A、中央着丝粒染色体	B、双着丝粒染色体	C、近端着丝粒染色体	D、亚中央着丝粒染色体

97. 18-三体综合症的体征有(	)

A:前脑无裂畸形	B:摇椅样畸形	C:小颌	D:先天性心脏病

98. 人类染色体中，可观察到随体结构的染色体有(	)

A:12	B:13	C:19	D:21

99. 以下哪条染色体条带的书写方式是正确的(	)

A:1p22.1	B:1p21	C:1q22.1	D:1p1.3

100. 染色体多态即正常变异通常没有明显的表观效应和病理学意义，下面包含有多态描述的核型是(	)

A:46，XX，16qh+	B:46，XX,13cenh+pat	C:46，XY，22pvar	D:46，XX，inv(9)(p12q13)

101. X染色体失活的Lyon假说包括(	)

A:失活发生在胚胎发育早期	B:X染色体的失活是随机的	C:失活是完全的	D:失活是永久性的和克隆式繁殖的

102. 人类染色体包括(	)

A:中着丝粒染色体	B:亚中着丝粒染色体	C:近端着丝粒染色体	D:端着丝粒染色体

103. 导致染色体发生畸变的原因主要有(	)

A:物理因素和化学因素	B:年龄因素	C:生物因素	D:遗传因素

104. Turner综合症的主要临床特征为(	)

A:体矮（身高120-140cm）	B:性腺发育不全，表型为女性	C:50%有蹼颈	D:有特殊面容

105. 13--三体综合征的体征有（	）

A：前脑无裂畸形	B：大睾丸	C：小颌	D：先天性心脏病

106. 关于染色体结构，以下描述正确的是（	）

A：染色质通过多级螺旋包装形成染色体 B：染色质的基本单位是核小体 C：核小体由核心颗粒和连接区两部分组成 D：H2A、H2B、H3、H4各2个分子形成的八聚体及其周围的DNA组成核心颗粒

107. 下列哪些疾病应该进行染色体检查（	）

A：Down综合征	B：Turner综合征 C：Klinefelter综合征	D：5p-综合征

108. 下列哪些显带技术可显示染色体的整体结构（	）

A：C显带	B：G显带	C：Q显带	D：R显带

109. Down综合征患者可根据核型组成不同，分为以下几种遗传学类型（	）

A：标准型	B：罗伯逊易位型	C :部分三体型	D：嵌合体型

110. 人类染色体中有随体的是（	）

A:D组	B:E组	C:F组	D:G组(Y除外)

111. 以下核型属于性染色体病的是（	）

A:47,XXY	B:45,X	C:46,XY/46,XX	D:47,XXX

112. 下列哪种疾病需要进行染色体检查（	）

A:地中海贫血	B:Down综合征 C:Turner综合征	D:Klinefelter综合征

113. 下列哪种属于常染色体疾病（	）

A:13-三体综合征	B: 18-三体综合征 C: 21-三体综合征	D: Turner综合征

114. 下列哪些显带技术可显示染色体的特殊结构（	）

A:C显带	B:G显带	C:T显带	D:N显带

115. Turner综合征的异常核型包括（	）

A:45,X	B: 45,X/46,XX	C:46,X,del(X)(P11)	D: 46,X,del(X)(q11)

116. 以下哪条染色体条带的书写方式不正确的（	）

A: 8P1.1	B: 8P21.1	C: 8q2.1	D: 8q11.1

117. 以下核型属于Down综合征的有（	）

A:47,XX,+21 B: 47,XY,+21/46,XY C: 46,XY,der（6;21）（p23;q11）,+21 D:45,XY, der(13;21)(q10;q10)

118. 非整倍体异常的产生原因主要有（	）

A:在受精卵的卵裂早期的有丝分裂过程中发生的染色体不分离	B: 减数分裂时发生染色体不分离	C: 细胞有丝分裂过程中发生染色体丢失 D: 核内复制

119. 染色体C显带可使染色体以下那几个部位染色（	）

A. 着丝粒	B.次缢痕	C.短臂	D.Y染色体长臂远端

120. 下列哪些疾病应进行染色体检查（	）

A. Alport综合征	B.Down综合征 C.Edward综合征 D.Patau综合征

121. 整倍体异常产生机制主要有（ ）

A. 双雄受精和双雌受精	B.核内复制	C.染色体断裂	D.核内有丝分裂

122. 先天愚型的核型有（	）

A、47, XX(XY), ＋21	B、45, XY, －14, －21, ＋t(14q21q)	C、46, XX/47, XX, ＋21	D、46, －14, ＋t(14q21q)

123. 染色体断裂后不重新连接或连接错误可能导致的染色体结构畸变类型有（	）

A．缺失	B．重复	C．倒位	D．易位

124. 下列关于染色体核型分析的说法，正确的是（	）

A．可用于诊断染色体数目异常疾病	B．可用于诊断染色体结构异常疾病	C．通常选取分裂期细胞进行分析	D．需借助显带技术提高识别精度

125. 下列属于性染色体数目异常疾病的是（	）

A．Klinefelter综合征（47,XXY）	B．XYY综合征（47,XYY）	C．XXX综合征（47,XXX）	D．猫叫综合征（5p-）

126. 染色体易位根据是否发生在同源染色体之间，可分为（	）

A．同源易位	B．非同源易位	C．罗伯逊易位	D．相互易位

127. 下列关于染色体倒位的描述，正确的是（	）

A．染色体片段发生180°旋转	B．可能导致配子形成过程中染色体配对异常	C．倒位携带者通常表型正常	D．一定会引起严重的临床症状

128. 导致染色体发生丢失的原因可能是（	）

A．有丝分裂后期染色体未被拉向两极	B．减数分裂Ⅰ后期同源染色体未分离	C．减数分裂Ⅱ后期姐妹染色单体未分离	D．染色体断裂后片段丢失

129. 下列哪些细胞适合用于染色体检查（	）

A．外周血淋巴细胞	B．羊水细胞	C．绒毛细胞	D．皮肤成纤维细胞

130. 染色体病患者的皮肤纹理特征可能包括（	）

A．通贯手	B．三叉点t移向掌心	C．指纹中弓形纹增多	D．趾间纹异常

131. 下列关于罗伯逊易位的说法，正确的是（	）

A．发生在近端着丝粒染色体之间	B．两条染色体长臂连接形成一条衍生染色体	C．短臂通常丢失，一般不影响表型	D．易位携带者生育异常后代的风险较高

132. 染色体次缢痕与下列哪些结构功能相关（	）

A．核糖体RNA合成	B．随体形成	C．染色体复制起始	D．着丝粒功能维持

133. 下列属于染色体数目异常中的非整倍体类型的是（	）

A．单体型（2n-1）	B．三体型（2n+1）	C．四体型（2n+2）	D．多倍体（3n）

134. 下列因素中，可能通过影响DNA复制导致染色体畸变的是（	）

A．紫外线照射	B．化学诱变剂（如烷化剂）	C．病毒感染（如疱疹病毒）	D．温度剧烈变化

135. 下列关于染色体端粒的描述，正确的是（	）

A．维持染色体结构稳定	B．随细胞分裂次数增加而缩短	C．由重复的DNA序列组成	D．属于常染色质区域

136. 染色体检查中，G显带技术的原理是（	）

A．利用特殊染料对染色体不同区域的亲和力差异	B．使染色体出现明暗相间的条带	C．可区分不同大小和形态的染色体	D．主要显示染色体的异染色质区域

137. 下列疾病中，可能因染色体片段缺失导致的是（	）

A．猫叫综合征（5p-）	B．Prader-Willi综合征（15q11-13缺失）	C．Angelman综合征（15q11-13缺失）	D．Edward综合征（18-三体）

138. 下列关于染色体嵌合体的说法，正确的是（	）

A．由不同核型的细胞系组成	B．多因受精卵卵裂早期染色体不分离导致	C．临床症状严重程度与异常细胞比例相关	D．所有嵌合体都有明显的临床症状

139. 下列属于结构异染色质特征的是（	）

A．在细胞周期中始终高度螺旋化	B．基因密度低	C．复制时间晚	D．可发生随机失活

140. 染色体不分离发生在减数分裂Ⅰ和减数分裂Ⅱ，产生的异常配子类型差异在于（	）

A．减数分裂Ⅰ不分离产生的配子含同源染色体	B．减数分裂Ⅱ不分离产生的配子含姐妹染色单体	C．两者产生的配子均为非整倍体	D．减数分裂Ⅰ不分离产生的异常配子比例更高

141. 下列关于染色体重复的描述，正确的是（	）

A．染色体片段数量增加	B．可能因同源染色体不等交换导致	C．重复片段过大可能导致临床症状	D．所有重复都不会影响个体表型

142. 下列显带技术中，可用于识别Y染色体特异性区域的是（	）

A．C显带	B．Q显带	C．R显带	D．T显带

143. 染色体病的诊断依据包括（	）

A．典型的临床症状和体征	B．染色体核型分析结果	C．家族遗传史	D．生化指标异常

144. 下列关于核内复制的说法，正确的是（	）

A．细胞分裂时染色体复制但细胞质不分裂	B．可导致多倍体形成	C．常见于肿瘤细胞	D．对个体发育无影响

145. 下列属于近端着丝粒染色体特有的结构是（	）

A．随体	B．次缢痕（随体柄）	C．长臂	D．短臂

146. 下列因素中，可能诱发染色体畸变的生物因素包括（	）

A．细菌感染	B．病毒感染（如风疹病毒）	C．寄生虫感染	D．真菌毒素

147. 下列关于染色体倒位的临床影响，正确的是（	）

A．倒位携带者可能产生不平衡配子	B．倒位片段越小，临床影响越大	C．臂间倒位比臂内倒位影响更大	D．部分倒位携带者可正常生育

148. 染色体检查的临床意义包括（	）

A．明确染色体病诊断	B．指导遗传咨询	C．评估生育风险	D．监测肿瘤治疗效果

149. 下列关于多倍体的描述，正确的是（	）

A．体细胞染色体数目为3n或更高	B．人类多倍体胚胎通常无法存活	C．可因双雄受精或双雌受精导致	D．与染色体不分离无关

150. 下列属于染色体结构异常疾病的是（	）

A．慢性粒细胞白血病（Ph染色体，t(9;22)）	B．视网膜母细胞瘤（13q14缺失）	C．Down综合征（21-三体）	D．Turner综合征（45,X）

151. 下列关于染色体条带命名规则的说法，正确的是（	）

A．染色体臂用p（短臂）和q（长臂）表示	B．区带编号从着丝粒向两端依次递增	C．亚带用小数点后数字表示（如p22.1）	D．条带编号越大，距离着丝粒越近

152. 染色体多态性的常见表现形式包括（	）

A．随体大小差异	B．次缢痕长度变化	C．Y染色体长臂长度差异	D．染色体片段缺失

153. 下列关于X染色体失活的说法，正确的是（	）

A．失活的X染色体形成巴氏小体	B．失活发生在胚胎发育早期	C．失活是随机且不可逆的	D．男性X染色体也会发生失活

154. 下列因素中，可能导致染色体断裂的物理因素包括（	）

A．X射线	B．γ射线	C．紫外线	D．超声波

155. 下列关于染色体病遗传特点的说法，正确的是（	）

A．常染色体病通常与父母核型无关（新发突变为主）	B．性染色体病部分与父母年龄相关	C．染色体结构异常可通过携带者传递	D．所有染色体病都有明显的家族聚集性

156. 下列显带技术中，不用于显示染色体端粒区域的是（	）

A．T显带	B．C显带	C．R显带	D．N显带

157. 下列关于染色体丢失的描述，正确的是（	）

A．导致细胞成为单体型	B．可能发生在有丝分裂或减数分裂过程中	C．丢失的染色体片段可重新连接	D．与染色体不分离机制相同

158. 下列疾病中，适合通过染色体检查确诊的是（	）

A．Klinefelter综合征	B．苯丙酮尿症（单基因病）	C．地中海贫血（单基因病）	D．21-三体综合征

159. 下列关于染色体着丝粒的描述，正确的是（	）

A．连接姐妹染色单体	B．是纺锤丝附着的部位	C．属于结构异染色质	D．与染色体分离密切相关

160. 下列关于染色体易位的临床影响，正确的是（	）

A．相互易位携带者通常表型正常	B．易位可能导致基因断裂或融合（如肿瘤）	C．罗伯逊易位携带者生育三体患儿风险高	D．所有易位都会导致严重疾病

161. 检测基因表达异常时，可考虑的检测材料有（	）

A．核基因组DNA   B．线粒体DNA   C．RNA D．蛋白质和酶

162. 多基因遗传特点是（	）

A. 两个极端个体的子代为中间型  B. 中间型个体的子代为中间型，存在小的变异 C. 中间型个体的子代多为中间型，并有较广的变异 D. 在随机婚配的群体中，多数个体为中间型，少数为极端型

163. 基因突变的特点有（	）

A、不可逆性 B、多向性 C、可重复性 D、有害性

164. 属于遗传病的治疗方法是（	）

A．外科治疗    B．内科治疗   C．基因治疗 D．饮食治疗

165. 遗传病的特征多表现为（	）

A．家族性  B．先天性   C．同卵双生率高于异卵双生率 D．不累及非血缘关系者

166. 属于静态突变的疾病有（ ）

A．β地中海贫血  B．镰状细胞贫血  C．半乳糖血症 D．Huntington舞蹈症

167. DNA复制过程的特点有（ ）

A．互补性   B．半保留性   C．反向平行性 D．子链合成的方向为5′→3′

168. 一种多基因遗传病的复发风险与以下那些因素有关（ ）

A．该病的一般群体的发病率的大小 B．亲缘关系的远近 C．近亲结婚 D．亲属中患病人数

169. 下列哪些是生命有机体的遗传物质（ ）

A．DNA B．RNA C．碱基 D．蛋白质

170. 有些多基因遗传病的群体发病率有性别差异，患者的后代发病风险有以下特点（	）

A．发病率低的性别，则患病阈值低，其子女的复发风险相对较低 B．发病率低的性别，则患病阈值高，其子女的复发风险相对较高 C．发病率高的性别，则患病阈值高，其子女的复发风险相对较高 D．发病率高的性别，则患病阈值低，其子女的复发风险相对较低

171. 真核细胞染色体的化学组成是（ ）

A．RNA   B．DNA C．组蛋白   D．非组蛋白

172. 核酸杂交结果判断的依据是（ ）

A．信号位置  B．信号强度  C．信号数量  D．信号种类

173. 常见的启动子序列包括（	）

A．TATA框 B．CAAT框  C．GC框   D．AG框

174. 判断是否是遗传病的指标为（	）

A．患者亲属发病率随亲属级别下降而下降 B．患者亲属发病率随亲属级别下降而升高 C．患者血缘亲属发病率高于非血缘亲属 D．患者家族成员发病率高于一般群体

175. 一个男性为血友病患者，其父母和祖父母都正常，其亲属中可能患血友病A的人是（ ）

A．舅父   B．姨表兄弟   C．外甥   D．同胞兄弟

176. 产前通过（	）检查可筛查Down综合征胎儿

A．甲胎蛋白    B．雌三醇     C．过氧化氢酶 D．绒毛膜促性腺激素

177. 可作为分子遗传标记的有（ ）

A．SNP  B．微卫星DNA   C．RFLP   D．HLA

178. 下列关于多基因遗传的正确说法是（ ）

A．微效基因和环境因素共同作用 B．多为两对以上等位基因作用 C．微效基因是共显性的    D．环境因素起到不可替代的作用

179. 真核细胞终止密码子包括（ ）

A．UAA； B．AUG； C．UAG； D．UGA；

180. 以下属于优生措施的是（ ）

A禁止近亲结婚 B提倡适龄生育 C进行产前诊断  D鼓励婚前检查

181. 常染色体病在临床上最常见的表型特征是（	）

A、不孕 B、自发流产 C、智力低下 D、多发畸形

182. 下列哪些细胞为单倍体（	）

A、精子 B、初级精母细胞 C、卵细胞 D、第一极体

183. X连锁隐性遗传病表现为（ ）

A、系谱中只有男性患者   B、女儿有病，父亲一定有病 C、父母无病，子女也无病   D、有交叉遗传

184. ISCN描述染色体结构畸变的简式包括内容有（    ）

A:染色体总数和性染色体组成 B:畸变类型的符号 C:在括号内写明受累的染色体序号 D: 在另一括号内以符号注明受累的染色体断裂点

185. 关于荧光原位杂交技术（FISH）的表述正确的是（	）

A.荧光标记的探针包括DNA、RNA或cDNA B.既可检测中期染色体，也可检测间期细胞 C.FISH技术是有快速、安全、灵敏度高和特异性强等优点 D.标本可以长期保存不失活

186. 染色体结构畸变的主要方式有（  ）

A、缺失 B、易位 C、倒位  D、环状染色体、等臂染色体

187. 染色体数目异常产生的原因是（	）

A、染色体丢失 B、染色体易位 C、染色体不分离 D、核内复制

188. 14/21染色体平衡易位携带者与正常个体婚配后，其所生子女的核型可能有（	）

A、14/21易位型先天愚型 B、14/21易位携带者  C、正常  D、 单纯性21-三体

189. 染色体数目异常的类型包括（   ）

A、三倍体 B、四倍体 C、亚二倍体 D、超二倍体

190. Lyon假说的内容包括（ ）

A、正常女性的两条X染色体中，只有一条具有转录活性，另一条在遗传上失活 B、哪一条X染色体失活是随机的 C、在女性胚胎发育初期，两条X染色体都有活性，其后一条失活 D、在一个细胞中失活的那条染色体，在其后增殖的细胞都是这条X染色体失活

191. 可以染色体检查的材料有（	）

A、全血 B、毛发 C、活检组织 D、羊水

192. 地贫患者中常用（ ）来评价血糖长期控制水平.

A.糖化白蛋白（GA） B.果糖胺（Fructosamine） C.HbA1c D.HbA2c

193. 地贫模式主要检测指标（ ）

A.HbA2 B.HbF C.HbA D.HbA1c

194. 患有贫血或是血红蛋白异常的人，可以用（	）方式来评价血糖控制水平

A.糖化血红蛋白 B.糖化血清白蛋白 C.糖化血清蛋白

195. 成人血红蛋白主要成分（ ）

A.HbA （α2β2）约占血红蛋白总量96.5—97.5%  B.HbA2 （α2δ2 ） 约占血红蛋白总量2.5—3.5% C.HbF （α2γ2）约占血红蛋白总量1%以下 D.Hb Gower 1 （α2α2 ）

196. 地中海贫血的遗传方式有（ ）

A. 常染色体显性遗传 B. 常染色体隐性遗传  C. X-连锁遗传 D. Y-连锁遗传

197. 有助于提高地中海贫血患者生活质量的措施包括（ ）

A. 定期复查 B. 合理饮食  C. 适当运动 D. 心理支持

198. . 重型β地中海贫血的可能基因型是（	）。

A. βA/βA B.δβ+/β0 C.β0/β0 D.δβA/δβ0

199. 以下关于地中海贫血描述错误的是？（	）

A. 单基因遗传性溶血性贫血 B. 珠蛋白生成障碍性贫血 C. 中国北方地区多发 D. 地中海贫血人群的血红蛋白有一对α链和一对β链组成

200. 巨幼细胞贫血的实验室检查特点包括：（	）

A. 大细胞性贫血 B. 骨髓中巨幼红细胞增多 C. 血清叶酸和/或维生素B12水平降低 D. 中性粒细胞分叶过多

201. 溶血性贫血的病因包括：（	）

A. 红细胞膜异常 B. 红细胞酶缺陷 C. 血红蛋白异常 D. 免疫因素

202. 以下哪些是贫血患者输血的适应证？（	）

A. 急性失血导致重度贫血 B. 严重贫血伴有缺氧症状 C. 慢性贫血患者难以耐受贫血 D. 贫血患者需要手术时

203. 再生障碍性贫血患者的血液学特点有：（	）

A. 全血细胞减少 B. 网织红细胞绝对值减少 C. 淋巴细胞比例增高 D. 骨髓增生低下

204. 贫血的诊断步骤包括：（	）

A. 详细询问病史 B. 全面体格检查 C. 必要的实验室检查 D. 明确贫血的类型和病因

205. 下列哪些情况可能导致缺铁性贫血？（	）

A. 长期素食 B. 胃肠道疾病影响铁吸收 C. 妇女月经量过多 D. 儿童生长发育过快

206. 基因芯片技术的优点在于（	）

A．大规模   B．微量化   C．高通量   D．自动化

207. 人类染色体中，可观察到随体结构的染色体有（	）

A.13    B.15     C.17     D.21

208. 以下哪条染色体条带的书写方式不正确的有（  ）

A.10q23.3     B.10q1.3     C.10p21.1    D.10q2.2

209. 染色体多态即正常变异，通常没有明显的表型效应和病理学意义，下面包含有多态描述的核型是（    ）

A.46，XY，21ps+     B.46，XX,qh+    C.46，XX，del(5)(q13)   D.46,X,Yqh-

210. 人类染色体包括（     ）

A:中央着丝粒染色体    B：亚中着丝粒染色体  C:近端着丝粒染色体    D:端着丝粒染色体

211. 以下属于贫血的临床表现的是（	）

A. 皮肤苍白 B. 头晕、乏力 C. 心悸、气促 D. 耳鸣、眼花

212. 缺铁性贫血患者可能出现的特殊表现有（	）

A. 异食癖 B. 匙状甲 C. 吞咽困难 D. 毛发干枯

213. 基因芯片技术的优势体现在哪些方面（	）

A．能实现大规模检测   B．样本用量微量化   C．检测过程高通量   D．操作流程自动化

214. 在人类染色体中，具备随体结构的染色体是（	）

A.13    B.15     C.17     D.21

215. 下列染色体条带书写存在错误的是（  ）

A.10q23.3     B.10q1.3     C.10p21.1    D.10q2.2

216. 下列核型中，属于染色体正常多态性描述的是（    ）

A.46，XY，21ps+     B.46，XX,qh+    C.46，XX，del(5)(q13)   D.46,X,Yqh-

217. 按照着丝粒位置分类，人类染色体包含的类型有（     ）

A:中央着丝粒染色体    B：亚中着丝粒染色体  C:近端着丝粒染色体    D:端着丝粒染色体

218. 染色体结构发生畸变时，主要的变异方式包括（	）

A、缺失 B、易位 C、倒位 D、环状染色体、等臂染色体

219. 下列属于染色体数目异常类型的是（   ）

A、三倍体 B、四倍体 C、亚二倍体 D、超二倍体

220. 能够作为分子遗传标记的物质有（ ）

A．SNP  B．微卫星DNA   C．RFLP   D．HLA

221. 真核生物中，常见的启动子序列包含（	）

A．TATA框 B．CAAT框  C．GC框   D．AG框

222. 在真核细胞中，属于终止密码子的是（ ）

A．UAA； B．AUG； C．UAG； D．UGA；

223. 下列细胞中，属于单倍体细胞的是（	）

A、精子 B、初级精母细胞 C、卵细胞 D、第一极体

224. 关于X连锁隐性遗传病的遗传特点，表述正确的是（ ）

A、系谱中只有男性患者   B、女儿有病，父亲一定有病 C、父母无病，子女也无病   D、有交叉遗传

225. 贫血患者在哪些情况下需要考虑输血治疗（	）

A. 急性失血引发重度贫血 B. 严重贫血且伴随缺氧症状 C. 慢性贫血患者无法耐受贫血状态 D. 贫血患者需进行手术治疗时

226. 医师在产前诊断时发现以下情形时（	）,应向孕妇及家属说明情况,并提出终止妊娠的医学意见。

A、胎儿患有严重遗传疾病或有严重缺陷的

B、孕妇患有传染病的

C、因患严重疾病,继续妊娠严重危害孕妇健康的

D、因患严重疾病,继续妊娠可能危及孕妇生命安全的

227. 1岁男孩来门诊，医生怀疑他患有21-三体综合症，动员家长对患儿进行染色体检查。医师应告诉家长，患儿如下（	）特点均支持这一诊断。

A.经常伸舌 B. 双侧通贯掌 C. 表现呆滞 D. 眼距宽

228. 以下不是5p-综合征的临床表现的是（	）。

A. 习惯性流产

B. 满月脸、猫叫样哭声

C、表型男性、乳房发育、小阴茎、隐睾

D、身材高大、性格暴躁、常有攻击性行为

229. 以下哪些不是易位染色体携带者在临床上可能表现(	)。

A. 习惯性流产

B. 满月脸、猫叫样哭声

C表型男性、乳房发育、小阴茎、隐睾

D身材高大、性格暴躁、常有攻击性行为

230. 以下哪些不是Klinefelter综合征的临床表现（	）。

A. 习惯性流产

B. 满月脸、猫叫样哭声

C、表型男性、乳房发育、小阴茎、隐睾

D、身材高大、性格暴躁、常有攻击性行为

231. 哪些不是常染色体平衡易位携带者最常见的临床表现是（	）

A. 自发流产

B. 不育

C. 智力低下

D. 多发畸形

232. 下列哪些核型不能诊断为真两性畸形（	）

A. 45, XY/47，XXX

B. 46, XY/46, XX

C. 47, XXY

D. 45, X

233. 产前诊断染色体标本可以从（	）获得。

A. 脐带血 B. 绒毛膜 C. 羊水 D. 外周血

234. 下列关于染色体核型书写规则的说法，正确的是（	）

A．先写染色体总数，再写性染色体组成  B．异常染色体用“+”“-”表示增减（如47,XX,+21）  C．易位用“t”表示（如t(9;22)）  D．嵌合体用“/”分隔不同核型（如45,X/46,XX）

235. 下列哪些符合常染色体显性遗传的特征_______。

A. 男女发病机会均等

B. 系谱中呈连续传递现象

C. 患者都是纯合体（AA），杂合体（Aa）是携带者

D. 双亲无病时，子女一般不会发病

236. 地贫基因携带者在婚育方面需要注意（ ）

A. 进行遗传咨询 B. 产前诊断 C. 了解配偶的地贫基因携带情况 D. 避免生育

237. 以下关于地中海贫血描述正确的是？（	）

A. 单基因遗传性溶血性贫血 B. 珠蛋白生成障碍性贫血 C. 中国北方地区多发 D. 地中海贫血人群的血红蛋白有一对α链和一对β链组成

238. 地中海贫血主要是由于哪种珠蛋白基因缺陷？（	）

A. α珠蛋白基因 B. β珠蛋白基因 C. γ珠蛋白基因 D. δ珠蛋白基因

239. 染色体不分离发生在减数分裂Ⅰ和减数分裂Ⅱ，产生的异常配子类型差异在于（	）

A．减数分裂Ⅰ不分离产生的配子含同源染色体  B．减数分裂Ⅱ不分离产生的配子含姐妹染色单体  C．两者产生的配子均为非整倍体  D．减数分裂Ⅰ不分离产生的异常配子比例更高

240. 下列那些遗传病不能通过染色体检查而确诊（	）。

A.苯丙酮尿症 B.白化病

C.血友病 D. Klinefelter综合征
