# Supplementary material for: Performance and limitations of four large language models in genetic counseling for thalassemia
Source: Front Digit Health. 2026 Aug 11;8:1902480. doi: 10.3389/fdgth.2026.1902480 (PMC13464380; doi:10.3389/fdgth.2026.1902480)
Supplement: Supplementary file 2 [file Datasheet2.docx]

1. 地中海贫血是一种：

A. 传染病

B. 自身免疫性疾病

C. 遗传性溶血性贫血

D. 营养缺乏性贫血

2. 地中海贫血最常见的类型是：

A. α-地中海贫血

B. β-地中海贫血

C. δ-地中海贫血

D. γ-地中海贫血

3. 重型β-地中海贫血患儿出生时：

A. 即有明显贫血症状

B. 一般无明显症状

C. 出现黄疸和肝脾肿大

D. 有骨骼畸形

4. 地中海贫血基因携带者：

A. 一定会发病

B. 一般无临床症状或症状轻微

C. 会有严重贫血

D. 不能结婚生育

5. 诊断地中海贫血最可靠的方法是：

A. 血常规检查

B. 血红蛋白电泳

C. 基因诊断

D. 血清铁测定

6. 轻型α-地中海贫血患者的血红蛋白电泳结果通常表现为：

A. HbA2升高

B. HbF升高

C. HbH带出现

D. 一般无异常发现

7. 以下哪种食物对地中海贫血患者补血效果不佳：

A. 红枣

B. 菠菜

C. 动物肝脏

D. 牛奶

8. 地中海贫血患者输血治疗时，为预防铁过载，常需同时使用：

A. 维生素C

B. 叶酸

C. 去铁胺

D. 维生素B12

9. 夫妇双方均为轻型β-地中海贫血基因携带者，他们生育重型β-地中海贫血患儿的概率是：

A. 0

B. 25%

C. 50%

D. 100%

10. 以下关于地中海贫血的描述，错误的是：

A. 多见于南方地区

B. 可以通过骨髓移植治愈

C. 患者不能进行体育锻炼

D. 婚前筛查可有效预防

11. 中间型α-地中海贫血最常见的类型是：

A. HbBart's胎儿水肿综合征

B. HbH病

C. 轻型α-地中海贫血

D. 静止型α-地中海贫血

12. 重型地中海贫血患者进行造血干细胞移植时，最佳的供者是：

A. 无关供者

B. 脐带血供者

C. 同胞全相合供者

D. 父母供者

13. 地中海贫血患者出现骨骼畸形的主要原因是：

A. 铁过载

B. 长期贫血导致骨髓代偿性增生

C. 维生素D缺乏

D. 遗传因素直接导致

14. 以下哪种实验室检查结果有助于鉴别地中海贫血和缺铁性贫血：

A. 血清铁蛋白

B. 网织红细胞计数

C. 白细胞计数

D. 血小板计数

15. 对于地中海贫血基因携带者的健康指导，错误的是：

A. 定期进行血常规检查

B. 多补充铁剂

C. 做好遗传咨询

D. 适当锻炼

16. 地中海贫血是一种（ ）遗传病。

A. 单基因

B. 多基因

C. 染色体异常

D. 线粒体

17. 地贫患者可能出现的症状不包括（ ）。

A. 贫血

B. 黄疸

C. 免疫力增强

D. 肝脾肿大

18. 地中海贫血主要影响人体的（ ）。

A. 呼吸系统

B. 消化系统

C. 造血系统

D. 泌尿系统

19. 轻型α-地中海贫血患者通常（ ）。

A. 不需要特殊治疗

B. 需要定期输血

C. 需要骨髓移植

D. 需要脾切除

20. 以下哪项检查有助于诊断地中海贫血？（ ）

A. 血常规

B. 尿常规

C. 心电图

D. 脑电图

21. β-地中海贫血重型患者的治疗手段通常不包括（ ）。

A. 输血治疗

B. 铁螯合剂治疗

C. 物理康复治疗

D. 造血干细胞移植

22. 地中海贫血患者的红细胞形态通常（ ）。

A. 正常

B. 呈小细胞低色素性改变

C. 呈大细胞性改变

D. 呈球形

23. 地贫基因携带者（ ）。

A. 一定会发病

B. 不会将基因遗传给后代

C. 可能没有明显症状

D. 不需要进行婚育指导

24. 预防地中海贫血最有效的方法是（ ）。

A. 药物预防

B. 饮食预防

C. 基因检测和遗传咨询

D. 运动预防

25. 地中海贫血症的主要症状是什么？

A. 贫血

B. 黄疸

C. 出血倾向

D. 以上都是

26. 地中海贫血症的遗传模式是？

A. 常染色体显性遗传

B. 常染色体隐性遗传

C. X染色体显性遗传

D. X染色体隐性遗传

27. 地中海贫血症的诊断方法包括哪些？

A. 血常规检查

B. 血红蛋白电泳

C. 基因检测

D. 以上都是

28. 地中海贫血症患者根治需要进行哪些治疗？

A. 输血治疗

B. 铁剂治疗

C. 脾切除手术

D. 骨髓移植

29. 地中海贫血症的预防措施包括哪些？

A. 婚前检查

B. 产前诊断

C. 新生儿筛查

D. 以上都是

30. 地中海贫血症患者需要避免哪些食物？

A. 高铁食物

B. 高脂肪食物

C. 高盐食物

D. 高糖食物

31. 地中海贫血症患者的铁过载情况如何检测？

A. 血液铁含量检测

B. 肝脏铁含量检测

C. 心脏铁含量检测

D. 以上都是

32. 地中海贫血症患者进行骨髓移植的成功率是多少？

A. 50%

B. 70%

C. 90%

D. 无法确定

33. 地中海贫血症患者需要定期进行哪些检查？

A. 血常规

B. 肝功能

C. 肾功能

D. 以上都是

34. 地中海贫血症患者可以进行哪些体育活动？

A. 游泳

B. 跑步

C. 瑜伽

D. 以上都是

35. 夫妇双方均为α地贫携带者，那儿女患中重型α地贫的概率是多少？

A. 3/4

B. 1/2

C. 1/4

D. 1/8

36. 夫妻双方只有一方携带地贫基因，其子女（）

A. 不会携带地贫基因

B. 其子女50%的概率是正常，50%的概率是地贫基因携带，不会生育患中间型或重型地贫的孩子

C. 为复合型地贫

D. 有1/4概率分别从父母那里遗传地贫基因

37. 下列哪种情况属于地中海贫血高风险夫妇？（）

A. 曾生育重型α地贫或HbH病患儿的夫妇

B. 夫妇双方均为α地贫基因携带者

C. 夫妇一方为α或β地贫基因携带者，配偶为β地贫基因复合α地贫基因携带者

D. 以上说法均正确

38. 高风险夫妇进行地中海贫血产前诊断的最佳时间是多少？（）

A. 孕24周以前进行

B. 孕20周以前进行

C. 孕18周以前进行

D. 孕21周以前进行

39. 孕期地中海贫血检查的最佳时间点是（）

A. 孕早期（建卡时）

B. 孕中期（产检时）

C. 孕晚期（产检时）

D. 孕前（备孕时）

40. 预防地贫的关键就要找出携带同型地贫基因的夫妻，因此，在临床推广中，当孕妇地贫基因阳性时，一定要建议其（）来做地贫基因检测，以此判断胎儿可能的地贫基因型别。

A. 父母

B. 孩子

C. 配偶

D. 哥哥

41. 地中海贫血有哪几种治疗方法

A. 定期输血

B. 骨髓移植

C. 上述两种方法

42. 地中海贫血主要是由于（ ）合成障碍所引起的。

A. 红细胞膜

B. 血红蛋白

C. 白细胞

D. 血小板

43. 在广西地区，以下哪种是α-地中海贫血最常见的缺失类型（ ）。

A. --SEA

B. -α3.7

C. -α4.2

D. αααanti-3.7

44. β-地中海贫血的基因缺陷主要是（ ）。

A. 点突变

B. 大片段缺失

C. 染色体易位

D. 基因重复

45. 轻型α-地中海贫血患者的血常规表现通常为（ ）。

A. 小细胞低色素性贫血

B. 正细胞正色素性贫血

C. 大细胞性贫血

D. 单纯血小板减少

46. 地贫患者可能出现的特殊面容是（ ）。

A. 满月脸

B. 地贫面容（颅骨增大、额部隆起等）

C. 二尖瓣面容

D. 面具脸

47. 对于重型β-地中海贫血患者，最常用、最基础的治疗方案是（ ）。

A. 定期输血和去铁治疗

B. 补充叶酸

C. 脾切除

D. 造血干细胞移植

48. 地贫基因检测的样本类型不包括（ ）。

A. 外周血

B. 口腔黏膜细胞

C. 头发

D. 羊水（产前诊断）

49. 中间型β-地中海贫血患者的血红蛋白水平一般在（ ）。

A. 正常范围

B. 30-60g/L

C. 60-90g/L

D. 90-120g/L

50. 地中海贫血患者容易出现的并发症不包括（ ）。

A. 胆结石

B. 下肢静脉曲张

C. 脾肿大

D. 生长发育迟缓

51. α型地贫主要为（ ）

A. α亚基缺损导致

B. ß亚基缺损导致

C. γ亚基缺损导致

D. δ亚基缺损导致

52. 地中海贫血产前诊断的指征包括：（）

A. 重型地贫患儿生育史夫妇

B. 同型地贫携带者夫妇

C. αβ复合型地贫携带者夫妇

D. 以上都是

53. 下列关于血红蛋白的组成说法错误的是：（）

A. 由4条珠蛋白链组成的四聚体结构

B. γ-珠蛋白主要在胎儿期表达

C. β-珠蛋白主要在成人期表达

D. α-珠蛋白主要在成人期表达

54. 下面哪种情况不属于中间型β-地中海贫血的发生机制（）

A. 显性β突变的杂合子

B. β突变的杂合子合并α基因三联体或四联体

C. β突变的杂合子合并α突变的杂合子

D. β-地中海贫血突变合并HPFH

55. 下面哪个点突变存在于α1基因上 （）

A. Hb CS

B. Hb QS

C. Hb Q-Thailand

D. Hb Binyang

56. 对于重型β-地贫患者的预期寿命，下面描述不正确的是：（）

A. 不接受输血等治疗的患儿期望寿命5岁

B. 接受不规则输血治疗的患者期望寿命13-19岁

C. 接受规则高量输血但不接受除铁治疗的患者期望寿命20岁以上

D. 接受规则高量输血和系统正规除铁治疗的患者可长期无病存活

57. 下面哪个突变属于显性β-地中海贫血突变（）

A. IVSII-654(C>T)

B. CD53（-T)

C. CD19 (AAC>AGC)

D. CD95（+A）

58. 超声诊断Hb Bart's水肿胎的首要特征是：（）

A. 胎儿大脑中动脉血流峰值速度

B. 心/胸比率增大

C. 胸腔积液

D. 肝脾肿大、腹围大于孕周

59. 下列个体，理论上临床表型最重的是：（）

A. - -/-α

B. - -/α^QS^α

C. - -/α^CS^α

D. - -/α^WS^α

60. 海南黎族中最常见的β-地中海贫血突变是（）

A. CD17（AAG>TAG）

B. CD41-42(-CTTT)

C. CD26(GAG>AAG)

D. IVSII-654(C>T)

61. 下列几种重要的人类单基因遗传病中，主要采用杂合子筛查策略的是：（）

A. 地中海贫血

B. 囊性纤维化

C. 苯丙酮尿症

D. G-6-PD缺乏症

62. 下列哪种基因型可能表现为中间型地贫，也可能是重型地贫。

A. β⁰/ β⁺

B. β⁰/β⁰

C. β⁰/βᴺ

D. β⁺/β⁺

63. 关于PCR产物电泳分析的陈述，下列哪个正确

A. 于负极点样，向正极泳动

B. 电泳后离点样点越远的为长片段，离点样点越近的为短片段

C. 产物片段越长，琼脂糖凝胶的浓度越大

D. 于正极点样，向负极泳动

64. 抽提的基因组DNA，用分光光度计检测所获得的OD260/280值合适范围为：

A. 1.0－1.4

B. 1.4－1.6

C. 1.7－2.0

D. 1.9－2.1

65. 基因组DNA抽提方法不包括

A. 磁珠法

B. 酚氯法

C. 柱吸附法

D. TRIzol提取法

66. 在α-地贫和β-地贫人群中，绝大部分只具有地贫特征，称之为

A. 静止型地贫

B. 地贫基因携带者

C. 地贫患者

D. 中间型地贫

67. 下列哪种基因型一般都表现为重型地贫

A. β⁺/βᴺ

B. β⁰/β⁰

C. β⁰/βᴺ

D. β⁺/β⁺

68. 下列基因型中，不属于HbH病的是

A. - -/-α

B. - -/αα

C. - -/α^CS^α

D. - -/α^QS^α

69. 正常新生儿脐血中含量为70%的血红蛋白是

A. Hb A

B. Hb A2

C. Hb F

D. Hb Portland

70. 下面哪种异常血红蛋白不属于地中海贫血样变异的异常血红蛋白

A. Hb E

B. Hb CS

C. Hb QS

D. Hb M

71. 绒毛膜取样的最佳孕周时间是：

A. 16--20周

B. 9--11周

C. 24--28周

D. 11--16周

72. 下列属于无创产前诊断的方法是：

A. 绒毛膜取样

B. 脐带穿刺

C. 羊膜腔穿刺

D. 外周全血采集

73. 当夫妇均为(- -SEA/)杂合子时，生育Hb Bart's水肿胎的概率为：

A. 1/2

B. 1/4

C. 1/8

D. 1/16

74. 据统计，每年全世界有超过_______万个遗传性血红蛋白病的患儿出生，包括镰状细胞性贫血或其他血红蛋白病以及地中海贫血。

A. 30

B. 50

C. 80

D. 100

75. 下列哪项不是已获准注册的地贫检测试剂盒使用的检测方法：（）

A. Gap-PCR

B. MLPA

C. RDB

D. PMCA

76. 下列哪项是地贫筛查的血液学分析方法：（）

A. 血常规检查

B. 成人血红蛋白电泳

C. 新生儿电泳

D. 以上都是

77. 广西地中海贫血初筛红细胞指数MCV和MCH分别为：（）

A. <83fl，<27pg

B. <82fl，<27pg

C. <82fl，<30pg

D. <83fl，<25pg

78. 胎儿地中海贫血产前诊断技术最佳取样孕周时间是 。

A. 绒毛 9-11周、羊水 16-20周、脐血 24-28周

B. 绒毛 9-14周、羊水 16-22周、脐血 24-28周

C. 绒毛 9-13周、羊水 16-20周、脐血 22-28周

D. 绒毛 9-11周、羊水 16-22周、脐血 24-28周

79. β-珠蛋白基因簇的组成顺序是

A. 5'-ε- ^G^γ-^A^γ-Ψβ-δ-β-3'

B. 5'-ε- ^G^γ-^A^γ-Ψβ-β-δ -3'

C. 5'-ε- ^A^γ-^G^γ-Ψβ-δ-β-3'

D. 5'-ε- ^G^γ-^A^γ-δ- Ψβ-β-3'

80. 广西地中海贫血筛查血常规阳性标准是

A. MCV>83fl MCH<26pg

B. MCV<82fl MCH<27pg

C. MCV>82fl MCH>26pg

D. MCV<83fl MCH<26pg

81. 血红蛋白分析HbA2的正常值范围、HbF的正常值范围

A. 2.3-3.3%、<1.0%

B. 2.5-3.5%、<2.0%

C. 2.0-3.0%、<1.0%

D. 2.2-3.3%、<3.0%

82. 我国地中海贫血基因突变类型及频率与下列哪个人群最为接近（）

A. 东南亚人群

B. 地中海人群

C. 非洲人群

D. 中亚人群

83. 地贫是属于哪一种遗传病？

A. 常染色体显性遗传病

B. 常染色体隐性遗传病

C. X染色体显性遗传病

D. X染色体隐性遗传病

84. 地中海贫血主要是由于（ ）异常引起的。

A. 白细胞

B. 血红蛋白

C. 血小板

D. 红细胞膜

85. 以下哪种是α-地中海贫血的常见类型（ ）

A. 静止型

B. 轻型

C. 中间型

D. 以上都是

86. 地中海贫血在我国哪个地区发病率较高（ ）

A. 东北地区

B. 西北地区

C. 华南地区

D. 华北地区

87. 诊断地中海贫血最常用的方法是（ ）

A. 血常规

B. 血红蛋白电泳

C. 基因检测

D. 骨髓穿刺

88. 重型β-地中海贫血患者通常需要（ ）治疗。

A. 输血

B. 补铁

C. 补钙

D. 手术

89. 地中海贫血的遗传方式是（ ）

A. 常染色体显性遗传

B. 常染色体隐性遗传

C. 性染色体显性遗传

D. 性染色体隐性遗传

90. 静止型α-地中海贫血患者的基因缺失情况是（ ）

A. 缺失 1 个α基因

B. 缺失 2 个α基因

C. 缺失 3 个α基因

D. 缺失 4 个α基因

91. 轻型地中海贫血患者一般（ ）

A. 有严重贫血症状

B. 无明显症状或轻度贫血

C. 需要长期输血

D. 发育迟缓

92. 预防地中海贫血的关键措施是（ ）

A. 婚前检查

B. 孕期检查

C. 新生儿筛查

D. 以上都是

93. 以下哪项不是地中海贫血患者可能出现的症状（ ）

A. 面色苍白

B. 头晕乏力

C. 皮肤瘙痒

D. 肝脾肿大

94. 地贫筛查应该在（）进行

A. 妊娠中期

B. 妊娠前或在妊娠早期

C. 妊娠晚期

D. 妊娠前或妊娠中期

95. 同型β地贫的轻型患者之间婚配再发风险，后代为轻型β地贫患儿的概率为（）。

A. 3/4

B. 1/2

C. 1/4

D. 1/8

96. 地贫的诊断、分型需要通过（ ）诊断？

A. 基因检测

B. 血液分析

C. 血红蛋白成分分析

D.血常规分析

97. 世界地贫日"是哪一天（）

A．5月8日

B．6月8日

C．7月8日

D. 7月9日

98. 血红蛋白的组成（ ）

A. 珠蛋白

B. 亚铁血红素

C. 白蛋白

D. 珠蛋白和亚铁血红素

99. β珠蛋白基因簇位于（ ）号染色体上

A. 7号染色体

B. 8号染色体

C. 9号染色体

D. 11号染色体

100. α珠蛋白基因簇位于（ ）号染色体上

A. 13号染色体

B. 14号染色体

C. 15号染色体

D. 16号染色体

101. 临床常用的α地贫检测包括哪种（ ）

A. 缺失型

B. 非缺失型

C. 突变型

D. 缺失型和非缺失型

102. α地贫的临床表现哪个属于静止型（ ）

A. αα/αα

B. -α/αα或αTα/αα

C. --/αα、-α/αTα

D. --/-α或--/ααT

103. 我国广西广东哪种地贫比较常见

A. α地贫

B. β地贫

C. α地贫与β地贫混合型

D. γ地贫

104. 一方筛查是阳性，一方筛查正常，还需不需要做基因检测。（ ）

A. 需要

B. 不需要

C. 不确定

105. 地中海贫血的发病机制是什么？

A. 基因突变

B. 染色体异常

C. 病毒感染

D. 营养不良

106. 地中海贫血的主要临床表现是什么？

A. 贫血

B. 黄疸

C. 出血倾向

D. 感染易感性

107. 地中海贫血的诊断主要依靠什么？

A. 临床表现

B. 实验室检查

C. 家族史

D. 影像学检查

108. 地中海贫血的治疗主要包括哪些？

A. 输血治疗

B. 药物治疗

C. 骨髓移植

D. 以上都是

109. 地中海贫血（Thalassemia）是一种：

A. 遗传性血液疾病

B. 传染性疾病

C. 免疫性疾病

D. 代谢性疾病

110. 地中海贫血主要影响以下哪个器官？

A. 心脏

B. 肝脏

C. 脾脏

D. 骨髓

111. 地中海贫血是由于以下哪种蛋白质合成异常导致的？

A. 血红蛋白

B. 白蛋白

C. 球蛋白

D. 纤维蛋白

112. 地中海贫血的主要症状不包括以下哪项？

A. 贫血

B. 黄疸

C. 脾脏肿大

D. 肌肉萎缩

113. 地中海贫血患者通常需要以下哪种治疗？

A. 药物治疗

B. 手术治疗

C. 输血治疗

D. 物理治疗

114. 地中海贫血患者输血治疗的主要目的是：

A. 补充营养

B. 提高免疫力

C. 提高血红蛋白水平

D. 预防感染

115. 地中海贫血患者输血治疗可能引起的并发症不包括：

A. 铁过载

B. 感染

C. 过敏反应

D. 营养不良

116. 地中海贫血患者进行基因治疗的目的是：

A. 改善症状

B. 根治疾病

C. 预防感染

D. 延长寿命

117. 地中海贫血的本质是

A. 传染病

B. 自身免疫性疾病

C. 遗传性溶血性贫血

D. 营养缺乏性贫血

118. 最常见的地中海贫血类型是

A. α型

B. β型

C. γ型

D. δ型

119. 重型β地中海贫血患儿出生时

A. 即有明显贫血症状

B. 一般无明显症状

C. 出现黄疸和肝脾肿大

D. 有骨骼畸形

120. 地中海贫血基因携带者的特点

A. 一定会发病

B. 一般无临床症状或症状轻微

C. 会有严重贫血

D. 不能结婚生育

121. 诊断地中海贫血最可靠的方法是

A. 血常规检查

B. 血红蛋白电泳

C. 基因诊断

D. 血清铁测定

122. 轻型α地中海贫血患者的血红蛋白电泳结果

A. HbA2升高

B. HbF升高

C. HbH带出现

D. 一般无异常发现

123. 对地中海贫血患者补血效果不佳的食物是

A. 红枣

B. 菠菜

C. 动物肝脏

D. 牛奶

124. 输血治疗时预防铁过载的药物是

A. 维生素C

B. 叶酸

C. 去铁胺

D. 维生素B12

125. 夫妇均为轻型β地中海贫血携带者，生育重型患儿的概率是

A. 0%

B. 25%

C. 50%

D. 100%

126. 关于地中海贫血的错误描述是

A. 多见于南方地区

B. 可通过骨髓移植治愈

C. 患者不能进行体育锻炼

D. 婚前筛查可有效预防

127. 重型α-地中海贫血胎儿的基因型通常是：

A. αα/αα

B. --/αα

C. --/--

D. -α/αα

128. 下列哪种类型的地中海贫血最常见：

A. α - 地中海贫血

B. β - 地中海贫血

C. δ - 地中海贫血

D. γ - 地中海贫血

129. 轻型β - 地中海贫血患者的血红蛋白含量一般为：

A. 正常

B. 轻度降低（90 - 120g/L）

C. 中度降低（60 - 90g/L）

D. 重度降低（＜60g/L）

130. 地中海贫血的主要发病机制是：

A. 红细胞膜缺陷

B. 珠蛋白合成障碍

C. 缺乏铁元素

D. 维生素B12 缺乏

131. 地中海贫血诊断和分型最常用的实验室检查方法是：

A. 血常规

B. 血红蛋白电泳

C. 基因检测

D. 血清铁测定

132. 重型β - 地中海贫血患者通常需要：

A. 定期输血治疗

B. 补充维生素C

C. 补铁治疗

D. 运动锻炼

133. 孕妇在孕期筛查地中海贫血，一般在：

A. 孕早期（12 周前）

B. 孕中期（13 - 28 周）

C. 孕晚期（28 周后）

D. 分娩后

134. 地中海贫血患者易出现的骨骼改变主要是因为：

A. 铁过载

B. 骨髓造血代偿性增生

C. 缺乏维生素D

D. 长期卧床

135. 以下哪种情况会加重地中海贫血患者的贫血症状：

A. 感染

B. 充足睡眠

C. 均衡饮食

D. 适度运动

136. 地中海贫血患者的贫血类型通常是：

A. 大细胞性贫血

B. 小细胞低色素性贫血

C. 正细胞正色素性贫血

D. 单纯小细胞性贫血

137. 对于有地中海贫血家族史的夫妇，孕前应进行：

A. 血常规检查

B. 血型检查

C. 地中海贫血基因筛查

D. 肝功能检查

138. 以下关于地中海贫血与缺铁性贫血的鉴别，错误的是：

A. 地中海贫血血清铁和铁蛋白正常或升高，缺铁性贫血降低

B. 地中海贫血MCV 降低更明显

C. 缺铁性贫血有明确的缺铁原因

D. 两者血红蛋白电泳结果一样

139. 重型β - 地中海贫血患儿常出现的特殊面容不包括：

A. 头颅变大

B. 额部隆起

C. 眼距增宽

D. 皮肤黝黑

140. 地中海贫血患者进行脾切除的主要指征是：

A. 贫血加重

B. 巨脾伴脾功能亢进

C. 感染

D. 铁过载

141. 地中海贫血基因携带者的血红蛋白水平一般：

A. 明显降低

B. 轻度降低

C. 正常或接近正常

D. 极度降低

142. 以下哪种地中海贫血类型相对症状最轻：

A. 静止型α - 地中海贫血

B. 轻型β - 地中海贫血

C. 中间型α - 地中海贫血

D. 重型β - 地中海贫血

143. 地中海贫血患者输血治疗的主要目的是：

A. 提高免疫力

B. 补充血浆蛋白

C. 维持血红蛋白水平

D. 促进造血干细胞增殖

144. 地中海贫血患者体内的珠蛋白肽链比例失调会导致：

A. 白细胞异常

B. 红细胞膜受损

C. 血小板减少

D. 凝血功能障碍

145. 孕妇筛查地中海贫血时，若发现异常，其配偶应：

A. 无需检查

B. 进行血常规检查

C. 进行地中海贫血相关检查

D. 补充叶酸

146. 重型地中海贫血患者常见的并发症不包括：

A. 胆结石

B. 生长发育迟缓

C. 感染

D. 高血压

147. 以下哪项不是地中海贫血的预防措施：

A. 婚前筛查

B. 孕期筛查

C. 避免近亲结婚

D. 多吃含铁食物

148. 地中海贫血患者使用去铁胺进行去铁治疗的途径是：

A. 口服

B. 皮下注射

C. 静脉注射

D. 肌肉注射

149. 地中海贫血患者的红细胞形态通常表现为：

A. 球形

B. 椭圆形

C. 靶形

D. 镰刀形

150. 以下关于地中海贫血的说法，错误的是：

A. 是一种先天性疾病

B. 南方地区发病率高于北方

C. 所有类型都有明显症状

D. 可以通过基因诊断

151. 中间型地中海贫血患者的血红蛋白水平一般在：

A. 30 - 60g/L

B. 60 - 90g/L

C. 90 - 120g/L

D. 120 - 150g/L

152. 地中海贫血患者出现黄疸的原因是：

A. 肝脏损伤

B. 红细胞破坏增多

C. 胆红素生成减少

D. 胆管阻塞

153. 以下哪种类型的地中海贫血病情相对较轻（ ）。

A. 重型α -地中海贫血

B. 中间型α -地中海贫血

C. 轻型β -地中海贫血

D. 重型β -地中海贫血

154. 地中海贫血在全球范围内，高发地区不包括（ ）。

A. 地中海沿岸

B. 东南亚

C. 北欧

D. 中国南方

155. 轻型β -地中海贫血患者的血红蛋白水平一般（ ）。

A. 低于60g/L

B. 60 - 90g/L

C. 90 - 120g/L

D. 正常或轻度降低

156. 以下哪项检查对地中海贫血的诊断最有价值（ ）。

A. 血常规

B. 骨髓穿刺

C. 血红蛋白电泳

D. 凝血功能检查

157. 重型α -地中海贫血胎儿在孕期常出现（ ）。

A. 胎儿水肿

B. 早产

C. 生长发育迟缓

D. 皮肤黄疸

158. 地中海贫血患者可能会出现（ ）增多。

A. 铁蛋白

B. 叶酸

C. 维生素B12

D. 白细胞介素

159. 对于地中海贫血患者的治疗，以下哪种说法错误（ ）。

A. 轻型患者一般无需特殊治疗

B. 重型患者可能需要输血治疗

C. 造血干细胞移植可根治重型地中海贫血

D. 铁剂治疗对所有患者都有效

160. 地中海贫血的主要症状不包括：

A. 贫血

B. 黄疸

C. 脾肿大

D. 高血压

161. 地中海贫血的诊断主要依靠：

A. 临床表现

B. 家族史

C. 实验室检查

D. 以上都是

162. 地中海贫血的基因突变类型不包括：

A. 点突变

B. 插入突变

C. 缺失突变

D. 染色体易位

163. 地中海贫血的基因治疗研究中，常用的基因载体是：

A. 病毒载体

B. 质粒载体

C. 细菌载体

D. 酵母载体

164. 地中海贫血患者需要定期输血的主要目的是：

A. 补充营养

B. 提高免疫力

C. 纠正贫血

D. 预防感染

165. 地中海贫血患者输血治疗的主要并发症是：

A. 过敏反应

B. 输血相关性肝炎

C. 铁过载

D. 输血相关性肺炎

166. 地中海贫血患者铁过载的治疗措施不包括：

A. 铁螯合剂治疗

B. 定期输血

C. 限制铁的摄入

D. 定期放血

167. 地中海贫血的产前诊断主要依靠：

A. 超声检查

B. 羊水穿刺

C. 基因检测

D. 胎儿镜检查

168. 地中海贫血患者的心理支持不包括：

A. 家庭支持

B. 社会支持

C. 经济支持

D. 专业心理咨询

169. 地中海贫血的预防措施不包括：

A. 婚前检查

B. 遗传咨询

C. 产前诊断

D. 定期输血

170. 地中海贫血的基因治疗研究中，常用的基因编辑技术是：

A. CRISPR/Cas9

B. TALENs

C. ZFNs

D. 以上都是

171. 地中海贫血患者需要定期监测的指标不包括：

A. 血红蛋白水平

B. 血清铁蛋白水平

C. 肝功能

D. 血压

172. 地中海贫血患者的生活指导不包括：

A. 合理饮食

B. 避免剧烈运动

C. 定期输血

D. 避免感染

173. 地中海贫血的基因治疗研究中，常用的基因表达调控元件是：

A. 启动子

B. 增强子

C. 沉默子

D. 以上都是

174. 地中海贫血患者需要定期进行的检查不包括：

A. 血常规

B. 肝功能

C. 肾功能

D. 心电图

175. 地中海贫血的基因治疗研究中，常用的基因载体类型不包括：

A. 腺病毒载体

B. 慢病毒载体

C. 逆转录病毒载体

D. 细菌载体

176. 以下哪种不属于α地中海贫血的类型（ ）

A. 静止型

B. 轻型

C. 中间型

D. 重型β

177. 夫妻双方都是轻型地中海贫血患者，胎儿有（ ）概率是重型患者。

A. 1/2

B. 1/4

C. 3/4

D. 1/8

178. 对于地中海贫血患者，以下饮食建议正确的是（ ）

A. 多吃含铁丰富食物

B. 避免食用含铁丰富食物

C. 随意饮食

D. 多吃海鲜

179. 以下哪种是常见的地中海贫血类型？（ ）

A. 缺铁性贫血

B. 巨幼细胞贫血

C. α地中海贫血

D. 再生障碍性贫血

180. 地中海贫血基因诊断主要检测什么？（ ）

A. 血红蛋白含量

B. 白细胞数量

C. 基因序列

D. 血小板计数

181. 重型α地中海贫血胎儿最常在孕期多少周出现水肿？（ ）

A. 10 - 14 周

B. 14 - 20 周

C. 20 - 24 周

D. 24 - 28 周

182. 轻型β地中海贫血患者一般有什么表现？（ ）

A. 发育迟缓

B. 重度贫血

C. 面色苍白

D. 可无明显症状

183. 用于地中海贫血基因诊断的常用技术不包括？（ ）

A. 聚合酶链反应

B. 基因测序

C. 免疫比浊法

D. 荧光原位杂交

184. 中间型地中海贫血患者的血红蛋白水平一般在？（ ）

A. 30 - 60g/L

B. 60 - 90g/L

C. 90 - 120g/L

D. 120 - 150g/L

185. 地中海贫血患者铁过载时，常用什么药物去铁？（ ）

A. 维生素 C

B. 叶酸

C. 去铁胺

D. 铁剂

186. 地中海贫血高发的地区是

A. 华南和西南

B. 华中

C. 华北

D. 西北

187. 以下关于地中海贫血防控的关键环节，错误的是：

A. 婚前筛查

B. 孕前筛查

C. 产前诊断

D. 产后治疗

188. 广西壮族自治区产前诊断技术管理办法实施细则第十一条规定，产前筛查和产前诊断《母婴保健技术服务执业许可证》有效期为（ ）年。

A: 二年 B:三年 C: 四年 D: 五年

189. 《中华人民共和国母婴保健法》第二十九条规定，（ ）级以上地方人民政府卫生行政部门管理本行政区域内的母婴保健工作。

A:乡 B：县 C :地市 D: 省

190. 广西壮族自治区产前诊断技术管理办法实施细则第六条规定（ ）及以上的医疗保健机构可以申请开展产前筛查技术服务。

A:一级甲等 B:二级甲等 C:三级甲等 D:三级丙等

191. 地中海贫血患者的贫血类型多为：

A. 巨细胞性贫血

B. 正细胞正色素性贫血

C. 小细胞低色素性贫血

D. 单纯小细胞性贫血

192. 《产前诊断技术管理办法》自（ ）开始实施？

A2003年5月1日 B2006年5月1日

C2013年5月1日 D2004年5月1日

193. 开展产前诊断技术的《母婴保健技术服务执业许可证》每（ ）年校验一次?

A 四 B 二 C 三 D 六

194. 产前筛查报告需（ ）以上相关技术人员核对后方可签发。其中，审核人应具备（ ）以上检验或相关专业的技术职称/职务。

A 两个，中级 B两个，副高级

C 一个，高级 D两个，初级

195. 实验室每年应参加（ ）卫生部指定机构的室间质评计划，并取得合格证书。连续（ ）不参加或者未取得室间质评合格证书的产前筛查视为质量控制不合格。

A 1-2次，2年 B 2-3次，2年 C 1-2次，3年 D 2-3次，3年

196. 医师在产前诊断时发现以下情形之一时,应向孕妇及家属说明情况,并提出终止妊娠的医学意见,除外（ ）

A、胎儿患有严重遗传疾病或有严重缺陷的

B、孕妇患有传染病的

C、因患严重疾病,继续妊娠严重危害孕妇健康的

D、因患严重疾病,继续妊娠可能危及孕妇生命安全的

197. 家系调查的最主要目的是( )。

A．了解发病人数 B．了解疾病的遗传方式

C．了解医治效果 D．收集病例

198. 异常血红蛋白病发病的机制主要是（ ）。

A. 点突变 B. 基因缺失

C. mRNA前体加工障碍 D. 融合基因

199. 成人血红蛋白以下列（ ）为主。

A. HbA2 B. HbA C. HbH D. Hb PortLand

200. 产前筛查是指通过简便、经济和较少创伤的检测方法，从孕妇群体中发现某些有（ ）的高风险孕妇，以便进一步明确诊断。

A 先天性缺陷和遗传性疾病胎儿 B 先天性缺陷和传染性疾病胎儿 C 先天性缺陷和痴呆儿 D先天性缺陷和无脑儿

201. 下列哪种属于染色体结构畸变（ ）

A:13-三体综合征 B: 18-三体综合征

C:21-三体综合征 D: 5p-综合征

202. 下列哪种核型表示等臂染色体（ ）

A:45,XX,t(14;21)(p11,q11) B:46,X,i（Xq）

C:46,XX,del(1)(q21) D:46,XX, r(2)(p21;q31)

203. 下列哪种核型可诊断为真两性畸形（ ）

A:46,XY/47,XXY B:45,X C: 47,XXY D:46,XY/46,XX

204. 按照ISCN的标准系统，1号染色体，长臂，2区，2带第2亚带应表示为（ ）

A: 1p2.22 B: 1p22.2 C: 1q2.22 D:1q22.2

205. 下列哪组染色体为近端着丝粒染色体（ ）

A: C组 B: D组 C: E组 D:F组

206. 一个体细胞中的全部染色体，按其大小、形态、特征顺序排列所构成的图称（ ）

A:组型 B: 核型 C:图形 D:表型

207. 染色质和染色体是（ ）

A: 同一物质在细胞的不同时期的两种不同的存在形式

B: 不同物质在细胞的不同时期的两种不同的存在形式

C: 同一物质在细胞的同一时期的不同表现

D:不同物质在细胞的同一时期的不同表现

208. 染色体显带技术中整条染色体显带技术包括（ ）

A:G显带、Q显带 、R显带 B:C 显带、T显带、R显带

C: C显带、T显带 、N显带 D:G显带、Q显带、N显带

209. 一个个体中含有不同染色体数目的三个细胞系，这种情况称为（ ）

A:三体型 B:三倍体 C:嵌合体 D:非整倍体

210. 罗伯逊易位发生的原因是（ ）

A:两条近端着丝粒染色体在着丝粒处断裂后相互融合形成一条染色体。

B: 两条近端着丝粒染色体在任何一处断裂后相互易位形成一条染色体。

C:任何两条染色体在着丝粒处断裂后相互融合形成一条染色体。

D:一条染色体节断插入另一条染色体。

211. 染色体结构畸形的基础是（ ）

A:染色体断裂 B:染色体复制

C:染色体不分离 D:姐妹染色单体交换

212. 染色体丢失发生在细胞分裂的（ ）期。

A:前期 B: 间期 C:中期和后期 D: 末期

213. 四倍体的形成可能是（ ）

A:双雄受精 B:双雌受精 C:核内复制 D: 染色体丢失

214. 14/21易位携带者婚后流产一个男胎，这个男胎的核型最可能是（ ）

A: 46,XY

B: 46,XY,der(14;21)(q10;q10),+21

C: 46,XY,der(14;21)(q10;q10),+14

D: 45,XY,der(14;21)(q10;q10)

215. klinefelter 综合征的核型为（ ）

A:46,XX/46,XY B:47,XXY C:47,XYY D:47,XXX

216. Edwards 综合征的病因是由于多了以下哪一条染色体（ ）

A: 13号 B: 18号 C：21号 D:22号

217. 染色体病的普遍特点是（ ）

A:生长发育迟缓，先天下心脏病，特异性异常体征

B: 生长发育迟缓，智力低下，特异性异常体征

C: 先天下心脏病，智力低下,特异性异常体征

D: 先天下心脏病，肾脏畸形，特异性异常体征

218. 发生于近端着丝粒染色体间的易位是（ ）

A:相互易位 B:染色体内易位 C :罗伯逊易位 D: 移位

219. 下列哪种简式表示环状染色体（ ）

A: 46,XX,del（q21） B: 46,XX,r(2)(p21;q31)

C: 46,XX,t(3;10)(q21;q22) D : 46,XX,inv(2)（p21q31）

220. 先天性睾丸发育不良的最常见核型是（ ）

A :45，X B: 47,XXX C: 47,XXY D : 47,XYY

221. 按照ISCN的标准系统，2号染色体，短臂，3区，2带第2亚带应表示为（ ）

A : 2p32.2 B: 2q32.2 C : 2p3.22 D : 2q3.22

222. 根据Denver体制，X染色体列入（ ）

A : A组 B : B组 C : C组 D : D组

223. 在核型中每对染色体，其中一条来自父方，一条来自母方，在形态结构上基本相同，称为（ ）

A:染色单体 B：染色体 C： 姐妹染色单体 D：同源染色体

224. 染色体显带技术中整条染色体的显带技术是（ ）等。

A:Q显带、G显带 、R显带 B:T 显带、Q显带、R显带

C: Q显带、G显带 、N显带 D:C显带、T显带、R显带

225. 染色体的端粒区为（ ）

A：结构异染色质 B:兼性异染色质 C:常染色质 D：染色质

226. 如果体细胞中染色体的数目在二倍体的基础上增加一条可形成（ ）

A:三倍体 B： 三体型 C:单倍体 D:单体型

227. 46,XY,/47,XY,+21嵌合体产生的原因是（ ）

A:减数第一次分裂染色体不分离 B：减数第二次分裂染色体不分离

C: 有丝分裂染色体不分离 D:双雄受精

228. 引起染色体断裂的因素可能导致（）

A:三体性 B:单体性 C:部分单体性 D:三倍体

229. 人类染色体不包括（ ）

A: 中央着丝粒染色体 B:亚中着丝粒染色体

C:近端着丝粒染色体 D: 端着丝粒染色体

230. 临床上最常见的染色体数目异常是（ ）

A: 单体 B:三体 C: 三倍体 D: 四倍体

231. 下列哪种属于染色体结构畸变（ ）

A.13-三体综合症 B.18-三体综合症 C.21-三体综合征 D.5p-综合症

232. Turner综合症的核型为（ ）

A.45,X B.47,XXX C.47,XXY D.47,XYY

233. 一个染色体平衡易位携带者最常见的临床表征是（ ）

A.不育 B.智力低下 C.自发流产 D.多发畸形

234. 染色体病的普遍特点是（ ）

A. 生长发育迟缓，智力低下，特异性异常体征

B. 生长发育迟缓，先天性心脏病，特异性异常体征

C. 先天性心脏病，智力低下，特异性异常体征

D. 先天性心脏病，肾脏畸形，特异性异常体征

235. 染色体数目整倍性改变以（ ）倍体为主

A 单 B二 C三 D 四

236. 染色体不分离多发生在（ ）时同源染色体的不分离

A. 减数第一次分裂 B. 减数第二次分裂 C. 有丝分裂间期 D. 有丝分裂前期

237. DNA自我复制发生的时期是：（ ）

A、G₁期 B、S期 C、G₂期 D、M期

238. 适用于连锁交换定律的基因位于：（ ）

A、同源染色体 B、非同源染色体 C、常染色体 D、性染色体

239. 染色体制备过程中须加入下列哪种物质以获得大量分裂象细胞（ ）。

A.BrdU B.秋水仙素 C.吖啶橙 D.吉姆萨

240. 在显带染色体中不能作为界标的是：（ ）

A、着丝点 B、长臂末端 C、明显的带 D、亚带

241. 两对非同源染色体之间发生某个区段转移，从而引起变异的现象属于以下哪种染色体结构变异？（ ）

A.重复 B.易位 C.倒位 D.缺失

242. 下列哪种简式表示末端缺失（ ）。

A. 45，XX，t（14；21）（P11；q11）

B. 46，XX，del(1)(q21)

C. 46，XX，del(1)(q21；q31)

D. 46，XX，der(13；21)(q10；q10)

243. 受精卵第一次分裂(卵裂)时，如果发生体细胞不分离可以导致：（ ）

A．三倍体 B．单倍体 C．三体和单体的嵌合体 D．部分三体和部分单体的嵌合体

244. 18三体综合症的临床表现有（ ）。

A. 智力低下伴眼距宽、鼻梁塌陷、通贯掌、趾间距宽

B. 智力低下伴头皮缺损、多指、严重唇裂及腭裂

C. 智力低下伴肌张力亢进、特殊握拳姿势、摇椅足

D. 智力低下伴长脸、大耳朵、大下巴、大睾丸

245. 对先天性睾丸发育不全症患者，除了染色体核型分析外，还可以采用的方法是：（ ）

A、性染色体检查 B、生物化学检查 C、酶活性检查 D、基因检查

246. 46, XY, i(17)(q10)表示 （ ）。

A. 一女性体内发生了染色体的插入

B. 一男性体内发生了染色体的易位

C. 一男性带有等臂染色体

D. 一女性个体带有易位型的畸变染色体

247. 羊膜穿刺的最佳时期是：（ ）。

A、7-8周 B、8-16周 C、16-20周 D、20周

248. 1号染色体q21带和5号染色体q31带发生断裂，断裂点以远节段相互交换片段后重接，其详式为：（ ）。

A. 46，XX，t（1；5）（1pter→1p21：：5q31→5pter；5pter→5q31：：1p21→1pter）

B. 46，XX，t（1；5）（1pter→1q21：：5q31→5qter；5q31→5qter：：1q21→1qter）

C. 46，XX，t（1；5）（1pter→1q21：：5p31→5qter；5q31→5qter：：1p21→1qter）

D. 46，XX，t（1；5）（1pter→1q21：：5q31→5qter；5pter→5q31：：1q21→1qter）

249. 5pˉ综合征的临床表现有（ ）。

A. 习惯性流产

B. 满月脸、猫叫样哭声

C、表型男性、乳房发育、小阴茎、隐睾

D、身材高大、性格暴躁、常有攻击性行为

250. 脆性x染色体综合症患者的核型为（ ）

A.46,frax(q27)y	B. 47,frax(q27)y

C.46,fray(q27)x	D.46,fray(q27)y

251. 某一个体其体细胞中染色体的数目比二倍体多了3条，称为（ ）。

A.亚二倍体 B. 超二倍体 C.多倍体 D.嵌合体

252. 根据着丝粒位置和染色体大小，将人类22对常染色体由大到小依次命名为1至22号，并将人类染色体分为7组，其中属于D组的染色体是（ ）。

A．19\~22号染色体 B. 6\~12号染色体

C. 13\~15号染色体 D. 16\~18号染色体

253. 近端着丝点染色体之间的相互易位合称为：（ ）

A、单方易位 B、双方易位 C、罗伯逊易位 D、复杂易位

254. 在细胞有丝分裂时，染色单体发生分离是在：( )

A、前期 B、中期 C、后期 D、末期

255. 基因自由组合定律的细胞学基础：( )

A、同源染色体分离 B、非同源染色体自由组合

C、染色单体分离 D、非姊妹染色单体交换

256. 常染色质是指间期细胞核中( )。

A．螺旋化程度高，有转录活性的染色质

B．螺旋化程度低，有转录活性的染色质

C．螺旋化程度低，无转录活性的染色质

D．螺旋化程度高，无转录活性的染色质

257. 有些多基因遗传病的群体发病率有性别差异，发病率低的性别( )。

A.患性域值高，则其子女的复发风险相对较高

B.患性域值低，则其子女的复发风险相对较低

C.患性域值高，则其子女的复发风险相对较低

D.患性域值低，则其子女的复发风险相对较高

258. 下列哪项是人类最早确定的，也是最常见的一种染色体病( )。

A.21三体型 B.18三体型

C.5P-综合征 D.先天性卵巢发育不全征

259. 人类中的X染色体在核型中应属于哪一组：( )

A、E组 B、D组 C、C组 D、B组

260. 一个个体中含有不同染色体数目的三个细胞系，这种情况称为( )。

A.三体型 B.非整倍体 C.嵌合体 D.三倍体

261. 染色体上某一区段连同它带有的基因顺序发生180°倒转，从而引起变异的现象属于以下哪种染色体结构变异？( )

A.重复 B.易位 C.倒位 D.缺失

262. 易位染色体携带者在临床上可能表现出( )。

A. 习惯性流产

B. 满月脸、猫叫样哭声

C表型男性、乳房发育、小阴茎、隐睾

D身材高大、性格暴躁、常有攻击性行为

263. 45，XY，-21核型属于哪种变异类型（ ）。

A. 单体型 B. 三体型 C. 多体型 D. 单倍体

264. 猫叫样综合征患者的核型为（ ）。

A.46，XY，r(5)(p14) B. 46，XY，t(5;8)(p14;p15)

C. 46，XY，del(5)(p14) D. 46，XY，ins(5)(p14)

265. Klinefelter综合征的临床表现有（ ）。

A. 习惯性流产

B. 满月脸、猫叫样哭声

C、表型男性、乳房发育、小阴茎、隐睾

D、身材高大、性格暴躁、常有攻击性行为

266. 人类染色体的分组主要依据是（ ）

A. 染色体的长度

B. 染色体的宽度

C. 性染色体的类型

D. 染色体的相对长度和着丝粒的位置

267. 染色质的一级结构单位是（ ）

A. 染色丝

B. 断裂基因

C. 染色单体

D. 核小体

268. 受精卵第二次分裂时，卵裂球若发生有丝分裂染色体不分离，可以导致（）

A. 三倍体或单倍体

B. 三体或单体的纯合体

C. 部分三体或部分单体

D. 二倍体、三体和单体的嵌合体

269. 减数分裂第二次分裂发生个别染色体不分离，形成的配子中，将是（ ）

A. 1/4有n+1条染色体

B. 1/2有n+1条染色体

C. 1/2有n-1条染色体

D. 全部为n+1条染色体

270. 一男性患有脆性X综合征(CGG)n，其病因是n由60变为300。请问是发生以下哪种现象（ ）

A. 同义突变

B. 错义突变

C. 无义突变

D. 动态突变

271. 密码子是指（ ）

A. DNA分子中的三个随机的碱基对顺序

B. rRNA分子中的三随机的碱基顺序

C. tRNA分子中密码环上的三个碱基顺序

D. mRNA分子中三个相邻的碱基顺序

272. 发生联会的染色体是（ ）

A. 姐妹染色体

B. 非姐妹染色体

C. 同源染色体

D. 非同源染色体

273. 目前的遗传学观点认为，游离型21三体发生的最主要的病因是（ ）

A. 父亲年高，精子减数分裂时21号染色体不分离

B. 母亲年高，卵子减数分裂时，21号染色体不分离

C. 母亲怀孕期间有病毒感染

D. 母亲怀孕期间接触射线

274. 一个常染色体平衡易位携带者最常见的临床表现是（ ）

A. 自发流产

B. 不育

C. 智力低下

D. 多发畸形

275. 一个患者核型为46,XX/45,X，其发生原因可能是（ ）

A. 体细胞染色体不分离

B. 减数分裂染色体不分离

C. 染色体丢失

D. 双受精

276. 下列哪个核型可诊断为真两性畸形（ ）

A. 45, XY/47，XXY

B. 46, XY/46, XX

C. 47, XXY

D. 45, X

277. 有些遗传病家系看不到垂直遗传的现象，这是因为（ ）

A. 该遗传病是体细胞遗传病

B. 该遗传病是线粒体病

C. 该遗传病是性连锁遗传病

D. 该遗传病的患者活不到生育年龄或不育

278. 染色体制备过程中须加入下列哪种物质以获得大量分裂象细胞（ ）

A. BrdU

B. 秋水仙素

C. 吖啶橙

D. 吉姆萨

279. 下面关于Q带描述正确的是（ ）

A. 是专门显示着丝粒的显带技术

B. 是专门显示染色体端粒的显带技术

C. 是专门显示核仁组织区的显带技术

D. 用荧光染料对染色体标本进行染色，然后在荧光显微镜下进行观察

280. 罗伯逊易位发生的原因是（ ）

A. 两条近端着丝粒染色体在着丝粒处断裂后相互融合形成一条染色体

B. 两条近端着丝粒染色体在任何一处断裂后相互易位形成一条染色体

C. 任何两条染色体在着丝粒处断裂后相互融合形成一条染色体

D. 一条染色体节段插入另一条染色体

281. 46，XY，t（2；5）（q21；q31）表示（ ）

A. 一女性体内发生了染色体的插入

B. 一男性体内发生了染色体的易位

C. 一男性带有等臂染色体

D. 一女性个体带有易位型的畸变染色体

282. 21-三体综合症的临床表现有（ ）

A. 智力低下伴眼距宽、鼻梁塌陷、通贯掌、趾间距宽

B. 智力低下伴头皮缺损、多指、严重唇裂及腭裂

C. 智力低下伴肌张力亢进、特殊握拳姿势、摇椅足

D. 智力低下伴长脸、大耳朵、大下巴、大睾丸

283. 婚前医学检查的内容是指（ ）

A. 进行有关性卫生的保健和教育

B. 进行遗传病知识的教育

C. 对新婚避孕知识及计划生育指导

D. 对严重遗传疾病、指定传染病和有关精神病的检查

284. 产前诊断技术项目不包括（ ）

A. 遗传咨询

B. 医学影像

C. 生化免疫

D. 不孕不育

285. 下列核型是猫叫综合征患者核型的是（ ）

A. 47，XX（XY），+13

B. 47，XX（XY），+18

C. 47, XX(XY), ＋21

D. 46，XX（XY），del(5)(p15)

286. （ ）于1953年提出DNA双螺旋结构，标志分子遗传学的开始。

A. Jacob和Momod

B. Watson和Crick

C. Khorana和Holley

D. Avery和McLeod

287. 真核细胞中染色体主要是由（ ）

A. DNA和RNA组成

B. DNA和组蛋白质组成

C. RNA和蛋白质组成

D. 核酸和非组蛋白质组成

288. 某一个体其体细胞中染色体的数目比二倍体多了3条，称为（ ）

A. 亚二倍体 B. 超二倍体 C. 多倍体 D. 嵌合体

289. 某一个体其体细胞中染色体的数目比二倍体多了一条，称为（ ）

A. 亚二倍体 B. 超二倍体 C. 多倍体 D. 嵌合体

290. 若某人核型为46，XX，del（1）（pter→q21：）则表明在其体内的染色体发生了（ ）

A. 缺失 B. 倒位 C. 易位 D. 插入

291. 13三体综合症的临床表现有（ ）

A. 智力低下伴头皮缺损、多指、严重唇裂及腭裂

B. 智力低下伴肌张力亢进、特殊握拳姿势、摇椅足

C. 智力低下伴长脸、大耳朵、大下巴、大睾丸

D. 智力正常、身材矮小、肘外翻、乳腺发育差、乳间距宽

292. 18三体综合症的临床表现有（ ）

A. 智力低下伴眼距宽、鼻梁塌陷、通贯掌、趾间距宽

B. 智力低下伴头皮缺损、多指、严重唇裂及腭裂

C. 智力低下伴肌张力亢进、特殊握拳姿势、摇椅足

D. 智力低下伴长脸、大耳朵、大下巴、大睾丸

293. Turner综合症(先天性卵巢发育不全)的临床表现有（ ）

A. 智力正常、身材矮小、肘外翻、乳腺发育差、乳间距宽

B. 智力低下伴头皮缺损、多指、严重唇裂及腭裂

C. 智力低下伴肌张力亢进、特殊握拳姿势、摇椅足

D. 智力低下伴长脸、大耳朵、大下巴、大睾丸

294. 产前诊断染色体标本可以从（ ）获得。

A. 脐带血 B. 绒毛膜 C. 羊水 D. 以上都正确

295. 细胞增殖周期是（ ）

A. 从一次细胞分裂结束开始，到下一次细胞分裂结束时为止所经历的全过程

B. 从一次细胞分裂开始，到下一次细胞分裂结束为止所经历的全过程

C. 从一次细胞分裂结束开始，到下一次细胞分裂开始时为止所经历的全过程

D. 从一次细胞分裂开始，到下一次细胞分裂开始为止所经历的全过程

296. 人类卵子中的染色体应为（ ）

A. 23,X B. 23,Y C. 23,X或Y D. 23,X和Y

297. 100个初级精母细胞完成减数分裂后，可产生的Y型精子应是（ ）

A. 100个 B. 200个 C. 300个 D. 400个

298. 100个初级精母细胞完成分裂后可产生的精子应为（ ）

A. 100个 B. 200个 C. 400个 D. 800个

299. 100个初级卵母细胞完成减数分裂后能产生的卵子是（ ）

A. 100个 B. 200个 C. 300个 D. 400个

300. 人类女性所产生的第二极体中含的染色体是（ ）

A. 23，X B. 23，Y C. 23X或Y D. 23X和Y

以下是按您要求重新排序后的题目，序号从301开始：

301. 在常染色体显性遗传病中，基因型Aa应表现为（ ）

A. 正常人 B. 患者 C. 重患者 D. 携带者

302. 下列不属于多基因遗传的是（ ）

A. 身高 B. 体重 C. 肤色 D. 红绿色盲

303. 染色体非整倍性改变的机理可能是（ ）

A. 染色体断裂 B. 染色体核内复制 C. 染色体倒位 D. 染色体不分离或染色体丢失

304. 若某人核型为46，XX，dup（3）（q12q21）则表明在其体内的染色体发生了（ ）

A. 缺失 B. 倒位 C. 重复 D. 插入

305. 若某人核型为46，XX，inv（9）（p12q31）则表明在其体内的染色体发生了（ ）

A. 缺失 B. 倒位 C. 易位 D. 插入

306. 1岁男孩来门诊，医生怀疑他患有21-三体综合症，动员家长对患儿进行染色体检查。医师应告诉家长，患儿如下特点均支持这一诊断，除了（ ）

A. 经常伸舌 B. 皮肤粗糙 C. 表现呆滞 D. 眼距宽

307. 猫叫样综合征患者的核型为（ ）

A. 46，XY，r(5)(p14) B. 46，XY，t(5;8)(p14;p15)

C. 46，XY，del(5)(p14) D. 46，XY，ins(5)(p14)

308. 14/21罗伯逊易位的女性携带者与正常人婚配，其生下Down综合征患儿的风险是（ ）

A. 1 B. 1/2 C. 1/3 D. 1/4

309. 染色体数目异常形成的可能性原因是( )

A. 染色体不分离或丢失、核内复制

B. 染色体倒位和不分离、核内复制

C. 染色体断裂和丢失

D. 核内复制

310. 45/47/46三种细胞系组成的嵌合体个体产生的原因为( )

A. 减数分裂后期Ⅰ不分离

B. 减数分裂后期Ⅱ不分离

C. 染色体丢失

D. 有丝分裂不分离

311. 对脆性X染色体综合征可用( )方法诊断

A. G显带 B. C显带 C. PCR D. 高分辨显带

312. 下列哪些染色体全部是近端着丝粒染色体( )

A. 2,8,16,Y B. 2,9,12,13

C. 9,16,18,20 D. 13,15,21,Y

313. patau综合症的病因是由于多了以下哪一条染色体( )

A. 13号 B. 18号 C. 21号 D. 22号

314. klinfelter综合症的核型为( )

A. 46XX/46XY B. 47,XXY C. 47,XYY D. 47,XXX

315. 下列哪种疾病应进行染色体检查( )

A. Turner综合症 B. 地中海贫血 C. 苯丙酮尿症 D. 白化病

316. 染色体病的普遍特点是( )

A. 先天性心脏病，智力低下，特异性异常体征

B. 先天性心脏病，肾脏畸形，特异性异常体征

C. 先天性心脏病，生长发育迟缓，特异性异常体征

D. 智力低下，生长发育迟缓，特异性异常体征

317. 染色体结构畸变的基础是（ ）

A. 姊妹染色单体交换 B. 染色体断裂及断裂后的变位重接

C. 染色体不分离 D. 染色体丢失

318. 有一男性，46条染色体，第五号染色体断臂从1区2带到末端缺失，正确核型表示为（ ）

A. 46,XY,dup(5)(qter→p12)

B. 46,XY,del(5)(qter→p12)

C. 46,XY,inv(5)(qter→p12)

D. 46,XY,t(5)(qter→p12)

319. 先天性卵巢发育不全的最常见核型是（ ）

A. 45,X B. 47,XXY C. 47,XYY D. 47,XXX

320. 按照ISCN的标准系统，1号染色体，短臂3区，1带第3亚带应表示为（ ）

A. 1p31.3 B. 1q31.3 C. 1p3.13 D. 1q3.13

321. 根据人类细胞遗传学国际命名体制的规定，正常女性核型的描述方式为（ ）

A. 46XX B. 46,XX C. 46;XX D. 46.XX

322. 染色体臂上作为界标的带（ ）

A. 一定是染色体副缢痕区

B. 一定是染色体上稳定、显著的形态特征

C. 一定是浅带

D. 一定是深带

323. 异染色质是间期细胞核中（ ）

A. 螺旋化程度高，有转录活性的染色质

B. 螺旋化程度低，有转录活性的染色质

C. 螺旋化程度高，无转录活性的染色质

D. 螺旋化程度低，无转录活性的染色质

324. 嵌合体形成的原因可能是（ ）

A. 卵裂过程中发生了联会的同源染色体不分离

B. 生殖细胞形成过程中发生了染色体丢失

C. 生殖细胞形成过程中发生了染色体不分离

D. 卵裂过程中发生了染色体不分离

325. 人类正常生殖细胞精子或卵子各含有一个染色体组，称为（ ）

A. 单倍体 B. 二倍体 C. 亚二倍体 D. 超二倍体

326. 染色体的端粒区为（ ）

A. 结构异染色质 B. 兼性异染色质 C. 常染色质 D. 染色质

327. 发生于近端着丝粒染色体间的易位是（ ）

A. 相互易位 B. 罗伯逊易位 C. 移位 D. 染色体内易位

328. 显带技术中常用的染料是（ ）

A. 吉姆萨染液 B. 苏木清 C. 胰蛋白酶 D. NaOH

329. 69,XYY的产生机制是（ ）

A. 双雄受精 B. 双雌受精 C. 核内复制 D. 染色体断裂

330. Edwards综合征的病因是由于多了以下哪条染色体（ ）

A. 13号 B. 18号 C. 21号 D. 22号

331. 克氏综合征的核型为（ ）

A. 46,XX/46,XY B. 47,XXY C. 47,XXX D. 47,XYY

332. 一个常染色体平衡易位携带者最常见的临床表现是（ ）

A. 不孕不育 B. 智力低下 C. 多发畸形 D. 自发流产

333. 染色体病的普遍性特点是（ ）

A. 先天性心脏病，智力低下，特异性异常体征

B. 先天性心脏病，肾脏畸形，特异性异常体征

C. 生长发育迟缓，智力低下，特异性异常体征

D. 生长发育迟缓，先天性心脏病，特异性异常体征

334. 下列哪种染色体结构畸变是不平衡的（ ）

A. 末端缺失 B. 臂间倒位 C. 相互易位 D. 罗伯逊易位

335. 下列哪种简式表示臂间倒位（ ）

A. 45,XX,t(14;21)(p11;q11)

B. 46,X i（Xq）

C. 46,XY,inv(2)(p21;q31)

D. 46,XY, inv(3)(p13;p24)

336. 一个人的核型为47,XXY,此人患有（ ）

A. Down,s综合征 B. 生殖腺发育不全症

C. 脆性X综合征 D. 先天性睾丸发育不全

337. 按照ISCN的标准系统，3号染色体，短臂，2区，1带第3亚带应表示为（ ）

A. 3q2.13 B. 3q21.3 C. 3p21.3 D. 3q21.3

338. 根据Denver体制，Y染色体列入（ ）

A. D组 B. E组 C. F组 D. G组

339. 由于一条染色体长短臂末端同时丢失，两断端黏合形成的染色体叫（ ）

A. 环状染色体 B. 等臂染色体 C. 衍生染色体 D. 中期染色体

340. 染色体制备过程中，可加入（ ）以获得大量分裂相细胞。

A. 吉姆萨 B. 吖啶橙 C. 秋水仙素 D. NaOH

341. 仅在某些细胞类型或特殊的发育阶段呈现凝缩状态的染色体称为（ ）

A. 结构异染色质 B. 兼性异染色质 C. Y染色质 D. X染色质

342. 如果染色体的数目在二倍体的基础上减少一条则形成（ ）

A. 单倍体 B. 单体型 C. 三倍体 D. 三体型

343. 人类生殖细胞染色体数为（ ）

A. 22 B. 23 C. 24 D. 46

344. 距离着丝粒最近的条带是（ ）

A. 10q11.2 B. 10q21.3 C. 10q24.3 D. 10q26.1

345. 近端着丝粒染色体之间通过着丝粒融合而形成的易位是（ ）

A. 相互易位 B. 罗伯逊易位 C. 复杂易位 D. 插入易位

346. 一条染色体长臂或断臂内发生二次断裂后，中间片段旋转180°后重接形成衍生染色体，称为（ ）

A. 相互易位 B. 罗伯逊易位 C. 臂间倒位 D. 臂内倒位

347. 1959年Lejune发现人类第一例染色体病为（ ）

A. 13-三体综合征 B. 18-三体综合征

C. 21-三体综合征 D. 克氏体综合征

348. 先天性卵巢发育不全症病人常见的核型为（ ）

A. 45,X B. 46,XX/46,XY C. 47,XXX D. 47,XXY

349. 染色体病的普遍特点是（ ）

A. 先天性心脏病，智力低下，特异性异常特征

B. 先天性心脏病,肾脏畸形，特异性异常特征

C. 生长发育迟缓，先天性心脏病，特异性异常特征

D. 生长发育迟缓，智力低下，特异性异常特征

350. 正常人类染色体核型中，C组的染色体为多少：（ ）

A、6对 B、7对 C、8对 D、7对和X染色体

351. 人类中最常见的染色体畸变类型是（ ）

A 单体型 B 二体型 C 三体型 D 四体型

352. 下列关于细胞周期的叙述正确的是（ ）

A．细胞周期是指细胞从开始分裂到分裂结束

B．从上一次分裂开始到下一次分裂结束

C．从上一次分裂结束开始到下一次分裂结束为止

D．从上一次分裂开始到下一次分裂开始之前

353. 遵照自由组合定律传递的基因位于：（ ）

A、同源染色体 B、非同源染色体 C、常染色体 D、性染色体

354. 在多基因病中由多基因基础决定的发生某种遗传病风险的高低称为（ ）。

A. 遗传度 B. 易感性 C. 易患性 D. 阈值

355. 13三体综合症的临床表现有（ ）。

A. 智力低下伴头皮缺损、多指、严重唇裂及腭裂

B. 智力低下伴肌张力亢进、特殊握拳姿势、摇椅足

C. 智力低下伴长脸、大耳朵、大下巴、大睾丸

D. 智力正常、身材矮小、肘外翻、乳腺发育差、乳间距宽

356. 按照ISCN的标准系统，1号染色体，短臂，3区，1带第3亚带应表示为（ ）。

A.1p31.3 B. 1q31.3 C. 1p3.13 D. 1q3.13

357. 染色体上增加了某个相同区段，从而引起变异的现象属于以下哪种染色体结构变异？（ ）

A.重复 B.易位 C.倒位 D.缺失

358. 46, XX, dic(12；15)(q11；q24)表示（ ）

A. 一女性体内发生了染色体的插入

B. 一男性体内发生了染色体的易位

C. 一男性带有等臂染色体

D. 一女性个体有双着丝粒的染色体

359. 从致病基因传递的角度考虑，X连锁隐性遗传病典型的传递方式为（ ）。

A. 女性→男性→女性 B. 男性→女性→男性

C. 女性→女性→女性 D. 男性→女性→女性

360. 母亲为红绿色盲，父亲正常，其四个儿子有（ ）个可能是色盲。

A.1 B.2 C.3 D.4

361. β珠蛋白位于（ ）号染色体上。

A. 6 B. 11 C. 14 D. 16

362. 镰状细胞贫血症的HbS与正常的HbA相比（ ）。

A. β链的第6位密码子发生了无义突变

B. β链的第6位密码子发生了移码突变

C. β链的第6位密码子发生了单碱基替换

D. α链的第6位密码子发生了单碱基替换

363. 单分体出现在减数分裂的哪个时期：（ ）

A、后期Ⅰ B、末期Ⅰ C、中期Ⅱ D、后期Ⅱ

364. 基因连锁互换定律的细胞学基础：（ ）

A、同源染色体分离 B、非同源染色体自由组合

C、染色单体分离 D、非姊妹染色单体交换

365. 同源染色体发生分离是在：（ ）

A、前期Ⅰ B、中期Ⅰ C、后期Ⅰ D、后期Ⅱ

366. 每一种生物，基因连锁群的数目应为：（ ）

A、染色体条数 B、同源染色体对数 C、常染色体数 D、性染色体数

367. 核型为47，XXXY的个体，其X、Y染色质的数目为（ ）。

A. X染色质 3 Y染色质 1 B. X染色质 2 Y染色质 1

C. X染色质 0 Y染色质 3 D. X染色质 2 Y染色质 0

368. 经检查，某患者的核型为46，XY，r(9)(p21q24)，说明其为（ ）患者。

A. 环状染色体 B.染色体丢失

C. 染色体倒位 D.染色体部分丢失

369. Xp缺失如包括整个短臂，则患者既有（ ）的体征，又有性腺发育不全。

A．Klinefelter综合征 B．Down综合征

C．Turner综合征 D．Edward综合征

370. 对多基因遗传病，后代发病风险的估计与下列哪个因素无关（ ）。

A. 群体发病率 B. 患者年龄

C. 家庭患病人数 D. 病情严重程度

371. 在先证者所患遗传病较严重且难于治疗，再发风险高，但患儿父母又迫切希望有一个健康的孩子的情况下，可选择的措施是（ ）。

A．人工授精 B．不再生育 C．借卵怀胎 D．产前诊断

372. 常因形成半合子而引起疾病的遗传病有（ ）。

A.AR B.AD C.XR D.XD

373. 属于不完全显性的遗传病为（ ）。

A.软骨发育不全 B.多指症

C.Hnutington舞蹈病 D.短指症

374. 下面哪种疾病属于分子病（ ）。

A. 肾结石 B. 糖原合成酶缺乏症

C. Turner综合征 D. 地中海贫血

375. 标准型α地中海贫血患者的基因型是（ ）。

A.αα/-α B.α-/-α C.- -/-α D.- -/- -

376. 下列哪项不是中孕期母血清学产前筛查的主要检查项目？（ ）

A 唐氏综合征 B 18-三体综合征

C 开放性神经管缺陷 D 13-三体综合征

377. 在减数分裂过程中，染色单体的分离发生在：（ ）

A、中期Ⅱ B、后期Ⅱ C、末期Ⅱ D、后期Ⅰ

378. 下面是关于基因型和表现型的叙述，其中错误的是（ ）

A．表现型相同，基因型不一定相同

B．基因型相同，表现型一般相同

C．在相同环境中，基因型相同，表现型一定相同

D．在相同环境中，表现型相同，基因型一定相同

379. 染色体结构畸变属于（ ）。

A.移码突变 B.动态突变 C.片段突变 D.转换

380. 下列何种疾病不是由染色体数目异常引起的：（ ）

A、21---三体型先天愚型 B、猫叫综合症

C、18三体综合征 D、先天性卵巢发育不全

381. 若某人核型为46，XX，dup（3）（q12q21）则表明在其体内的染色体发生了（ ）。

A.缺失 B.倒位 C.重复 D.插入

382. D组或G组染色体与21号染色体通过着丝粒融合而形成的易位称为（ ）。

A.单方易位 B.复杂易位 C.串联易位 D.罗伯逊易位

383. 染色体上某一区段及其带有的基因一起丢失，从而引起变异的现象属于以下哪种染色体结构变异？（ ）

A.重复 B.易位 C.倒位 D.缺失

384. 46，XY，t（2；5）（q21；q31）表示（ ）。

A.一女性体内发生了染色体的插入 B.一男性体内发生了染色体的易位

C.一男性带有等臂染色体 D.一女性个体带有易位型的畸变染色体

385. 某一个体核型为46,XX/47,XX,+21/45,XX,－21，可能是由于（ ）原因造成的。

A．减数分裂中第一次分裂时染色体不分离

B．减数分裂中第二次分裂时染色体不分离

C．受精卵第一次卵裂时染色体不分离

D．受精卵第二次卵裂之后染色体不分离

386. 一对夫妇婚后生出了严重的常染色体遗传病患儿，或丈夫患严重的常染色体遗传病，或丈夫为染色体易位的携带者，而且已生出了遗传病患儿，再次生育时再发风险高，又无产前诊断方法。这时可采取对策是（ ）。

A．借卵怀胎 B．过继或认领 C．不再生育 D．人工授精

387. 当一种多基因遗传病的群体发病率有性别差异时，群体发病率低的性别易患性阈值（ ）。

A. 极高 B. 低 C. 高 D. 不变

388. 外耳道多毛症属于（ ）。

A.Y连锁遗传 B.X连锁隐性遗传

C. X连锁显性遗传 D.常染色体显性遗传

389. 杂合子携带者与正常人结婚后，其F1代发病率为1/2，F1的患者再与正常人结婚其子代的发病率也为1/2，则该疾病可能属于（ ）。

A. 常染色体完全显性遗传病 B. 常染色体不规则显性遗传病

C. 常染色体共显性遗传病 D. 常染色体隐性遗传病

390. 胎儿血红蛋白主要是（ ）。

A. HbA2 B. HbA C. HbF D. Hb Gower1

391. β地中海贫血最主要的发病机制是（ ）。

A. 点突变 B. 基因缺失

C. mRNA前体加工障碍 D. 融合基因

392. 同源染色体发生联会是在：（ ）

A、前期Ⅰ B、中期Ⅰ C、后期Ⅰ D、前期Ⅱ

393. 位于同一对同源染色体上的许多非等位基因相互构成：（ ）

A、染色体 B、连锁群 C、基因群 D、基因库

394. 染色体结构畸变的基础是：（ ）

A、染色体单体交换 B、染色体不分离

C、染色体断裂 D、染色体丢失

395. 双雄受精产生的受精卵的核型不可能是（ ）。

A．69,XXX　 B．69,XXY 　C．69,XYY　 D．69,YYY

396. 随着医疗技术的提高，某些遗传病患者经治疗后能正常生活并生育后代、若干年后，这些疾病将如何变化：（ ）

A、无变化 B、发病率降低 C、发病率升高 D、突变率升高

397. 在世代间间断传代并且男性发病率高于女性的遗传病为（ ）。

A.AR B.AD C.XR D.XD

398. 多基因遗传病患者亲属的发病风险随着亲缘系数降低而骤降，下列患者的亲属中发病率最低的是（ ）。

A.儿女 B.孙子、孙女 C.侄儿、侄女 D.表兄妹

399. 人类的红绿色盲就是一个X-连锁隐性遗传的典型病例，如果女性色盲基因携带者与正常男性婚配，则儿子将有（ ）的机率发病，女儿则有（ ）的机率为携带者，（ ）的机率为正常。

A.1/2；1/2；1/2 B. 1/4；1/2；1/2

C.1/2；1/4；1/2 D. 1/2；1/2；1/4

400. 一条染色体发生两次断裂后断片颠倒180°后重接，结果造成（ ）。

A.缺失 B.倒位 C.易位 D.插入

401. 遗传病特指_______。

A. 先天性疾病

B. 家族性疾病

C. 遗传物质改变引起的疾病

D. 不可医治的疾病

402. 高血压是_______。

A. 单基因病

B. 多基因病

C. 染色体病

D. 线粒体病

403. 双亲的血型分别为A型和B型，子女中可能出现的血型是_______。

A. A型、O型

B. B型、O型

C. AB型、O型

D. A型、B型、AB型、O型

404. 遗传病中，当父亲是某病患者时，无论母亲是否有病，他们子女中的女孩全部患此病，这种遗传病最可能是_______。

A. 常染色体显性遗传病

B. 常染色体隐性遗传病

C. X连锁显性遗传病

D. X连锁隐性遗传病

405. 从致病基因传递的角度考虑，X连锁隐性遗传病典型的传递方式为_______。

A. 男性→男性→男性

B. 男性→女性→男性

C. 女性→女性→女性

D. 女性→男性→女性

406. 下列哪一条不符合常染色体显性遗传的特征_______。

A. 男女发病机会均等

B. 系谱中呈连续传递现象

C. 患者都是纯合体（AA），杂合体（Aa）是携带者

D. 双亲无病时，子女一般不会发病

407. 患者正常同胞有2/3为携带者的遗传病为_______。

A. 常染色体显性遗传

B. 常染色体隐性遗传

C. X连锁显性遗传

D. X连锁隐性遗传

408. 根据国际体制的规定，正常女性核型的描述方式是_______。

A. 46XX

B. 46，XX

C. 46XY

D. 46.XX

409. 人类染色体的分组主要依据是_______。

A. 染色体的大小

B. 染色体的类型

C. 性染色体的类型

D. 染色体的长度和着丝粒的位置

410. 下面关于同源染色体概念的叙述中，不正确的是_______。

A. 一条染色体经复制而形成的两条染色体

B. 一条来自父方、一条来自母方的两条染色体

C. 在减数分裂过程中能联会的两条染色体

D. 形状和大小一般都相同的两条染色体

411. 在胚胎发育期最先合成的血红蛋白是_______。

A. δ链和ε链

B. δ链和γ链

C. ε链和α链

D. β链和δ链

412. 关于血红蛋白的叙述，以下不正确的是_______。

A. 它是一种呼吸载体

B. 它是一种色素蛋白，由血红素和珠蛋白组成

C. 构成血红蛋白的氨基酸有极性和非极性两类

D. 珠蛋白含有5条肽链

413. 不能表达珠蛋白的基因是_______。

A. α

B. β

C. δ

D. ψβ

414. 以下关于异常血红蛋白病的叙述，不正确的是_______。

A. 异常血红蛋白是指珠蛋白发生变异的血红蛋白

B. 它是由血红蛋白分子结构异常造成

C. 由基因的变化或缺失引起

D. 以上所述都不正确

415. 镰状细胞贫血症的HbS与正常的HbA相比_______。

A. β链的第6位密码子发生了无义突变

B. β链的第6位密码子发生了移码突变

C. β链的第6位密码子发生了单碱基替换

D. α链的第6位密码子发生了单碱基替换

416. _______于1953年提出DNA双螺旋结构，标志分子遗传学的开始。

A. Jacob和Momod

B. Watson和Crick

C. Khorana和Holley

D. Avery和McLeod

417. 种类最多的遗传病是_______。

A. 单基因病

B. 多基因病

C. 染色体病

D. 体细胞遗传病

418. 下列_______碱基不存在于DNA中。

A. 胸腺嘧啶

B. 胞嘧啶

C. 鸟嘌呤

D. 尿嘧啶

419. 脱氧核糖核酸分子中的碱基互补配对原则为_______。

A. A-U，G-C

B. A-G，T-C

C. A-T，C-G

D. A-U，T-C

420. 子女发病率为1/4的遗传病为_______。

A. 常染色体显性遗传

B. 常染色体隐性遗传

C. X连锁显性遗传

D. X连锁隐性遗传

421. 在患者的同胞中有1/2的可能性为患者的遗传病为_______。

A. 常染色体显性遗传

B. 常染色体隐性遗传

C. X连锁显性遗传

D. X连锁隐性遗传

422. 在核型中的每对染色体，其中一条来自父方的精子，一条来自母方的卵子，在形态结构上基本相同，称为_______。

A. 染色单体

B. 染色体

C. 姐妹染色单体

D. 同源染色体

423. 以下不属于地中海贫血的是_______。

A. 肽链基因缺失，不能合成mRNA

B. 肽链基因变异，引起mRNA减少

C. 肽链基因连接，导致mRNA减少

D. 密码子发生突变，造成无功能的mRNA

424. 以下哪种疾病是由于谷氨酸-缬氨酸的替代_______。

A. HbS

B. HbC

C. HbE

D. HbN

425. 环境因素诱导发病的单基因病为_______。

A. Huntington舞蹈病

B. 蚕豆病

C. 白化病

D. 血友病A

426. 发病率最高的遗传病是_______。

A. 单基因病

B. 多基因病

C. 染色体病

D. 体细胞遗传病

427. 基因表达时，遗传信息的基本流动方向是_______。

A. RNA→DNA→蛋白质

B. hnRNA→mRNA→蛋白质

C. DNA→mRNA→蛋白质

D. DNA→tRNA→蛋白质

428. 由于突变使编码密码子形成终止密码，此突变为_______。

A. 错义突变

B. 无义突变

C. 终止密码突变

D. 移码突变

429. 不改变氨基酸编码的基因突变为_______。

A. 同义突变

B. 错义突变

C. 无义突变

D. 终止密码突变

430. 属于转换的碱基替换为_______。

A. A和C

B. A和T

C. T和C

D. G和T

431. 属于颠换的碱基替换为_______。

A. G和T

B. A和G

C. T和C

D. C和U

432. 同类碱基之间发生替换的突变为_______。

A. 移码突变

B. 动态突变

C. 片段突变

D. 转换

433. 在一个随机杂交的群体中，多基因遗传的变异范围广泛，大多数个体接近于中间类型，极端变异的个体很少。这些变异产生是由（ ）。

A.多基因遗传基础和环境因素共同作用的结果

B. 遗传基础的作用大小决定的

C. 环境因素的作用大小决定的

D. 多对基因的分离和自由组合的作用的结果

434. 目前的遗传学观点认为，游离型21三体发生的最主要的病因是：（ ）。

A．父亲年高，精子减数分裂时21号染色体不分离

B．母亲年高，卵子减数分裂时，21号染色体不分离

C．母亲怀孕期间有病毒感染

D．母亲怀孕期间接触射线

435. 染色体上某一区段及其带有的基因一起丢失，从而引起变异的现象属于以下哪种染色体结构变异？（ ）

A. 染色体丢失 B.易位 C.倒位 D.缺失

436. 对于X连锁隐性遗传病而言，男性发病率等于（ ）。

A. 致病基因频率 B. 致病基因频率的平方

C. 致病基因频率的2倍 D. 致病基因频率的1/2

437. 一对糖原沉淀病Ⅰ型携带者夫妇结婚后，其子女中可能患病的几率是（ ）。

A. 1 B. 1/2 C. 0 D. 1/4

438. α地中海贫血最主要的发病机制是（ ）。

A. 点突变 B. 基因缺失 C. mRNA前体加工障碍 D. 融合基因

439. 由于基因融合引起的分子病为（ ）。

A. 镰状细胞贫血 B. β地中海贫血 C.血友病A D. Hb Lepore

440. 人类遗传性疾病中不包括哪些（ ）。

A． 单基因遗传病 B．多基因遗传病 C．先天性疾病 D．染色体病

441. 异常克隆是指至少有（ ）细胞含有相同的额外染色体或染色体结构异常，或至少有（ ）细胞均丢失同一号染色体。

A 2个，2个 B 2个，3个 C 3个，3个 D 3个，2个

442. 每个产前诊断病例至少有（ ）细胞的核型图像照相记录，电子版或者相片（ ）。

A 2个，保存5年 B 5个，永久保存 C 2个 ，永久保存 D 5个，保存5年

443. 人类中最常见的染色体畸变类型是（ ）

A 单体型 B 二体型 C 三体型 D 四体型

444. 下列关于细胞周期的叙述正确的是（ ）

A．细胞周期是指细胞从开始分裂到分裂结束

B．从上一次分裂开始到下一次分裂结束

C．从上一次分裂结束开始到下一次分裂结束为止

D．从上一次分裂开始到下一次分裂开始之前

445. 遵照自由组合定律传递的基因位于：（ ）

A、同源染色体 B、非同源染色体 C、常染色体 D、性染色体

446. 正常人类染色体核型中，C组的染色体为多少：（ ）

A、6对 B、7对 C、8对 D、7对和X染色体

447. 一个常染色体平衡易位携带者最常见的临床表现是：（ ）

A．自发流产 B．不育 C．智力低下 D．多发畸形

448. 在多基因病中由多基因基础决定的发生某种遗传病风险的高低称为（ ）。

A. 遗传度 B. 易感性 C. 易患性 D. 阈值

449. 13三体综合症的临床表现有（ ）。

A. 智力低下伴头皮缺损、多指、严重唇裂及腭裂

B. 智力低下伴肌张力亢进、特殊握拳姿势、摇椅足

C. 智力低下伴长脸、大耳朵、大下巴、大睾丸

D. 智力正常、身材矮小、肘外翻、乳腺发育差、乳间距宽

450. 按照ISCN的标准系统，1号染色体，短臂，3区，1带第3亚带应表示为（ ）。

A.1p31.3 B. 1q31.3 C. 1p3.13 D. 1q3.13

451. 染色体上增加了某个相同区段，从而引起变异的现象属于以下哪种染色体结构变异？（ ）

A.重复 B.易位 C.倒位 D.缺失

452. 绒毛取样的最佳时期是：（ ）

A. 7-9周 B. 8-16周 C. 16-20周 D. 20周

453. 重型α地中海贫血患者的基因型是（ ）。

A.αα/-α B.α-/-α C.- -/-α D.- -/- -

454. 母亲为红绿色盲，父亲正常，其四个儿子有（ ）个可能是色盲。

A.1 B.2 C.3 D.4

455. β珠蛋白位于（ ）号染色体上。

A. 6 B. 11 C. 14 D. 16

456. 镰状细胞贫血症的HbS与正常的HbA相比（ ）。

A. β链的第6位密码子发生了无义突变

B. β链的第6位密码子发生了移码突变

C. β链的第6位密码子发生了单碱基替换

D. α链的第6位密码子发生了单碱基替换

457. 开展产前诊断技术的《母婴保健技术服务执业许可证》每（ ）年校验一次?

A 一 B 二 C 三 D 五

458. 产前筛查报告需（ ）以上相关技术人员核对后方可签发。其中，审核人应具备（ ）以上检验或相关专业的技术职称/职务。

A 三个，中级 B两个，中级

C 一个，高级 D两个，高级

459. 实验室每年应参加（ ）卫生部指定机构的室间质评计划，并取得合格证书。连续（ ）不参加或者未取得室间质评合格证书的产前筛查视为质量控制不合格。

A 1-2次，2年 B 2-3次，2年 C 1-2次，3年 D 2-3次，3年

460. 医师在产前诊断时发现以下情形之一时,应向孕妇及家属说明情况,并提出终止妊娠的医学意见,除外（ ）

A、胎儿患有严重遗传疾病或有严重缺陷的

B、孕妇患有传染病的

C、因患严重疾病,继续妊娠严重危害孕妇健康的

D、因患严重疾病,继续妊娠可能危及孕妇生命安全的

461. 多基因遗传病的遗传度越高，则表示该种多基因病（ ）。

A.主要是遗传因素的作用

B. 主要是遗传因素的作用，环境因素作用较小

C.主要是环境因素的作用

D. 主要是环境因素的作用，遗传因素作用较小

462. 一种多基因遗传病的复发风险与（ ）。

A.该病的遗传率的大小有关，而与一般群体的发病率的大小无关

B.一般群体的发病率的大小有关，而与该病的遗传率的大小无关

C.该病的遗传率和一般群体的发病率的大小都有关

D.亲缘关系的远近无关

463. 在细胞有丝分裂时，染色单体发生分离是在：( )

A、前期 B、中期 C、后期 D、末期

464. 基因自由组合定律的细胞学基础：( )

A、同源染色体分离 B、非同源染色体自由组合

C、染色单体分离 D、非姊妹染色单体交换

465. 常染色质是指间期细胞核中( )。

A．螺旋化程度高，有转录活性的染色质

B．螺旋化程度低，有转录活性的染色质

C．螺旋化程度低，无转录活性的染色质

D．螺旋化程度高，无转录活性的染色质

E．螺旋化程度低，很少有转录活性的染色质

466. 有些多基因遗传病的群体发病率有性别差异，发病率低的性别( )。

A.患性域值高，则其子女的复发风险相对较高

B.患性域值低，则其子女的复发风险相对较低

C.患性域值高，则其子女的复发风险相对较低

D.患性域值低，则其子女的复发风险相对较高

467. 下列哪项是人类最早确定的，也是最常见的一种染色体病( )。

A.21三体型 B.18三体型

C.5P-综合征 D.先天性卵巢发育不全征

468. 人类中的X染色体在核型中应属于哪一组：( )

A、E组 B、D组 C、C组 D、B组

469. 一个个体中含有不同染色体数目的三个细胞系，这种情况称为( )。

A.三体型 B.非整倍体 C.嵌合体 D.三倍体

470. 染色体上某一区段连同它带有的基因顺序发生180°倒转，从而引起变异的现象属于以下哪种染色体结构变异？( )

A.重复 B.易位 C.倒位 D.缺失

471. 易位染色体携带者在临床上可能表现出( )。

A. 习惯性流产

B. 满月脸、猫叫样哭声

C表型男性、乳房发育、小阴茎、隐睾

D身材高大、性格暴躁、常有攻击性行为

472. Turner综合症的临床表现有( )。

A. 智力正常、身材矮小、肘外翻、乳腺发育差、乳间距宽

B. 智力低下伴头皮缺损、多指、严重唇裂及腭裂

C. 智力低下伴肌张力亢进、特殊握拳姿势、摇椅足

D. 智力低下伴长脸、大耳朵、大下巴、大睾丸

473. 家系调查的最主要目的是( )。

A．了解发病人数 B．了解疾病的遗传方式

C．了解医治效果 D．收集病例

474. 当一种疾病的传递方式为男性→男性→男性时，这种疾病最有可能是( )。

A. 从性遗传 B. 限性遗传

C. Y连锁遗传病 D. X连锁显性遗传病

475. 丈夫为红绿色盲，妻子正常且其家族中无患者，如再生育，子女患色盲的概率为( )。

A.1/2 B.1/4 C.2/3 D.0

476. 轻型α地中海贫血患者的基因型是（ ）。

A.αα/-α B.α-/-α C.- -/-α D.- -/- -

477. 正常人与重型β地中海贫血患者结婚，其子女患轻型β地中海贫血的可能性是( )。

A.0 B.1 C.1/4 D.1/2

478. 一对糖原沉淀病Ⅰ型携带者夫妇结婚后，其子女中可能患病的几率是（ ）。

A. 1 B. 1/2 C. 0 D. 1/4

479. α地中海贫血最主要的发病机制是（ ）。

A. 点突变 B. 基因缺失 C. mRNA前体加工障碍 D. 融合基因

480. 对于X连锁隐性遗传病而言，男性发病率等于（ ）。

A. 致病基因频率 B. 致病基因频率的平方

C. 致病基因频率的2倍 D. 致病基因频率的1/2

481. 一表型正常的男性，其父亲为白化病患者。该男性与一正常女性结婚，婚后如生育，其子女中（ ）。

A.有1/4可能为白化病患者 B. 有1/4可能为白化病携带者

C. 有1/2可能为白化病携带者 D. 有1/2可能为白化病患者

482. 异常血红蛋白病发病的机制主要是（ ）。

A. 点突变 B. 基因缺失

C. mRNA前体加工障碍 D. 融合基因

483. 人类α珠蛋白基因蔟定位在（ ）。

A.11p12 B.16p13 C.16q12 D.11q12

484. 对人类而言，下列哪种细胞中有23条染色体：（ ）

A、精原细胞 B、初级精母细胞 C、初级卵母细胞 D、卵细胞或精细胞

485. 当基因为杂合子时，能够表现出来的性状为：（ ）

A、相对性状 B、遗传性状 C、显性性状 D、隐性性状

486. 一对夫妇表型正常，妻子的弟弟为白化病（AR）患者。假设白化病基因在人群中为携带者的频率为1/60，这对夫妇生育白化病患儿的概率为（ ）

A.1/4 B.1/360 C.1/240 D.1/120

487. 成人血红蛋白以下列（ ）为主。

A. HbA2 B. HbA C. HbH D. Hb PortLand

488. 人类β珠蛋白基因蔟定位在（ ）。

A.16p12 B.16q12 C.11p15 D.11q12

489. 由多对基因和环境因素共同作用产生的疾病称为：（ ）

A、 遗传病 B、多基因病 C、单基因病 D、染色体病

490. 同源染色体发生分离是在：（ ）

A、前期Ⅰ B、中期Ⅰ C、后期Ⅰ D、后期Ⅱ

491. 每一种生物，基因连锁群的数目应为：（ ）

A、染色体条数 B、同源染色体对数 C、常染色体数 D、性染色体数

492. 在先证者所患遗传病较严重且难于治疗，再发风险高，但患儿父母又迫切希望有一个健康的孩子的情况下，可选择的措施是（ ）。

A．人工授精 B．不再生育 C．借卵怀胎 D．产前诊断

493. 常因形成半合子而引起疾病的遗传病有（ ）。

A.AR B.AD C.XR D.XD

494. 属于不完全显性的遗传病为（ ）。

A.软骨发育不全 B.多指症

C.Hnutington舞蹈病 D.短指症

495. 下面哪种疾病属于分子病（ ）。

A. 肾结石 B. 糖原合成酶缺乏症

C. Turner综合征 D. 地中海贫血

496. 静止型α地中海贫血患者的基因型是（ ）。

A.αα/-α B.α-/-α C.- -/-α D.- -/- -

497. 一个基因或一对基因发生突变引起的疾病，叫：（ ）

A、遗传病 B、多基因病 C、单基因病 D、染色体病

498. 对未取得产前诊断类母婴保健技术考核合格证书的个人，擅自从事产前诊断或超越许可范围的，由县级以上人民政府卫生行政部门（ ）；情节严重的，按照《中华人民共和国执业医师法》吊销其医师执业证书。构成犯罪的，依法追究刑事责任

A给予警告或者责令暂停一个月以上三个月以下执业活动

B给予警告或者责令暂停三个月以上六个月以下执业活动

C给予警告或者责令暂停六个月以上一年以下执业活动

D给予警告或者责令暂停一年以上两年以下执业活动

499. 基因表达时，遗传信息的基本流动方向是（ ）。

A.RNA→DNA→蛋白质 B.hnRNA→mRNA→蛋白质

C.DNA→mRNA→蛋白质 D.DNA→tRNA→蛋白质

500. 生殖细胞成熟过程中，染色体数目减半发生在哪个时期：（ ）

A、增殖期 B、生长期

C、第二次成熟分裂期 D、第一次成熟分裂期

以下是重新编号的题目列表，序号从501开始：

501. 嵌合体形成的原因是个别染色体不分离，这发生在：（ ）

A、减数分裂 B、第一次减数分裂

C、第二次减数分裂 D、胚胎卵裂

502. 两条非同源染色体同时发生断裂，断片交换位置后重接，结果造成（ ）。

A.缺失 B.倒位 C.易位 D.插入

503. 染色体结构畸变的基础是 ：（ ）

A.姐妹染色单体交换 B.染色体核内复制

C.染色体断裂及断裂之后的异常重排 D.染色体不分离

504. 一个男孩是甲型血友病（XR）的患者，其父母和祖父母均正常，其亲属中不可能患此病的人是（ ）。

A.外祖父或舅父 B.姨表兄弟 C.姑姑 D.同胞兄弟

505. 先天性幽门狭窄是一种多基因遗传病，男性发病率为0.5%，女性发病率为0.1%，下列哪种情况发病率最高（ ）。

A.女性患者的儿子 B.男性患者的儿子

C.女性患者的女儿 D.男性患者的女儿

506. 经检测发现，某个体的细胞核中有2个X小体，表明该个体一个体细胞中有（ ）条X染色体

A. 1 B. 2 C. 3 D. 4

507. 下列核型中，属于亚二倍体是：（ ）

A、45 ,XO B、46 ,XX C、47,XXY D、47,XY, +21

508. 一条染色体发生两次断裂后断片颠倒180°后重接，结果造成（ ）。

A.缺失 B.倒位 C.易位 D.插入

509. 若患者体内既含男性性腺，又含女性性腺，则为 （ ）。

A.男性 B.两性畸形 C.女性 D.假两性畸形

510. 下列那种遗传病可通过染色体检查而确诊（ ）。

A.苯丙酮尿症 B.白化病

C.血友病 D. Klinefelter综合征

511. 21三体型先天愚型的最主要的病因是：（ ）。

A. 母亲怀孕期间接触辐射

B. 母亲是47，XXX患者

C. 母亲年高，卵子减数分裂时，染色体不分离

D. 父亲年高，精子减数分裂时，染色体不分离

512. 对重型β地中海贫血和某些遗传性免疫缺陷患者治疗的有效方法是施行（ ）。

A.骨髓移植术 B.肝细胞移植术

C.红细胞移植术 D.白细胞移植术

513. 单分体出现在减数分裂的哪个时期：（ ）

A、后期Ⅰ B、末期Ⅰ C、中期Ⅱ D、后期Ⅱ

514. 基因连锁互换定律的细胞学基础：（ ）

A、同源染色体分离 B、非同源染色体自由组合

C、染色单体分离 D、非姊妹染色单体交换

515. 根据着丝粒位置和染色体大小，将人类22对常染色体由大到小依次命名为1至22号，并将人类染色体分为7组，其中属于D组的染色体是（ ）。

A．19\~22号染色体 B. 6\~12号染色体

C. 13\~15号染色体 D. 16\~18号染色体

516. 近端着丝点染色体之间的相互易位合称为：（ ）

A、单方易位 B、双方易位 C、罗伯逊易位 D、复杂易位

517. 某一个体其体细胞中染色体的数目比二倍体多了3条，称为（ ）。

A.亚二倍体 B. 超二倍体 C.多倍体 D.嵌合体

518. 脆性x染色体综合症患者的核型为（ ）

A.46,frax(q27)y B. 47,frax(q27)y

C.46,fray(q27)x D.46,fray(q27)y

519. 1号染色体q21带和5号染色体q31带发生断裂，断裂点以远节段相互交换片段后重接，其详式为：（ ）。

A. 46，XX，t（1；5）（1pter→1p21：：5q31→5pter；5pter→5q31：：1p21→1pter）

B. 46，XX，t（1；5）（1pter→1q21：：5q31→5qter；5qter→5p31：：1p21→1qter）

C. 46，XX，t（1；5）（1pter→1q21：：5p31→5qter；5q31→5qter：：1p21→1qter）

D. 46，XX，t（1；5）（1pter→1q21：：5q31→5qter；5pter→5q31：：1q21→1pter）

520. 5pˉ综合征的临床表现有（ ）。

A. 习惯性流产

B. 满月脸、猫叫样哭声

C、表型男性、乳房发育、小阴茎、隐睾

D、身材高大、性格暴躁、常有攻击性行为

521. 遗传病诊断时所采集的完整的系谱应该至少包括（ ）以上的家庭成员。

A.一代 B.二代 C.三代 D.四代

522. 由于近亲婚配可以导致何种遗传病的发病风险增高最明显（ ）。

A. AD B. AR C. XD D. XR

523. 短指和白化病分别为AD和AR，并且基因不在同一条染色体上。现有一个家庭，父亲为短指，母亲正常，而儿子为白化病。该家庭再生育，其子女为短指的概率为（ ）。

A.1/2 B.1/4 C.3/4 D.1/8

524. 重型β地中海贫血的可能基因型是（ ）。

A. βA/βA B.δβ+/βA C.β0/β+ D.δβA/δβ0

525. 静止型α地中海贫血患者之间婚配，生出轻型α地中海贫血患者的可能性是（ ）。

A.0 B.1/8 C.1/4 D.1/2

526. 我国地中海贫血基因突变类型及频率与下列哪个人群最为接近（）

A. 东南亚人群

B. 地中海人群

C. 非洲人群

D. 中亚人群

527. 下面哪种情况不属于中间型β-地中海贫血的发生机制（）

A. 显性β突变的杂合子

B. β突变的杂合子合并α基因三联体或四联体

C. β突变的杂合子合并α突变的杂合子

D. β-地中海贫血突变合并HPFH

528. 下列几种重要的人类单基因遗传病中，主要采用杂合子筛查策略的是：（）

A. 地中海贫血

B. 囊性纤维化

C. 苯丙酮尿症

D. G-6-PD缺乏症

529. 下列哪种基因型可能表现为中间型地贫，也可能是重型地贫。

A. β⁰/ β⁺

B. β⁰/β⁰

C. β⁰/βᴺ

D. β⁺/β⁺

530. 在遗传咨询过程中，应遵从医学伦理学原则不包括：（）

A. 有利

B. 无害

C. 公正

D. 平等

531. 海南黎族中最常见的β-地中海贫血突变是（）

A. CD17（AAG>TAG）

B. CD41-42(-CTTT)

C. CD26(GAG>AAG)

D. IVSII-654(C>T)

532. 下面哪个点突变存在于α1基因上 （）

A. Hb CS

B. Hb QS

C. Hb Q-Thailand

D. Hb WS

533. 下面哪个突变属于显性β-地中海贫血突变（）

A. IVSII-654(C>T)

B. CD53（-T)

C. CD19 (AAC>AGC)

D. CD95（+A）

534. 超声诊断Hb Bart's水肿胎的首要特征是：（）

A. 胎儿大脑中动脉血流峰值速度

B. 心/胸比率增大

C. 胸腔积液

D. 肝脾肿大、腹围大于孕周

535. 下列个体，理论上临床表型最重的是：（）

A. - -/-α

B. - -/α^QS^α

C. - -/α^CS^α

D. - -/α^WS^α

536. 胎儿地中海贫血产前诊断技术最佳取样孕周时间是 。

A. 绒毛 9-11周、羊水 16-20周、脐血 24-28周

B. 绒毛 9-14周、羊水 16-22周、脐血 24-28周

C. 绒毛 9-13周、羊水 16-20周、脐血 22-28周

D. 绒毛 9-11周、羊水 16-22周、脐血 24-28周

537. β-珠蛋白基因簇的组成顺序是

A. 5'-ε- ^G^γ-^A^γ-Ψβ-δ-β-3'

B. 5'-ε- ^G^γ-^A^γ-Ψβ-β-δ -3'

C. 5'-ε- ^A^γ-^G^γ-Ψβ-δ-β-3'

D. 5'-ε- ^G^γ-^A^γ-δ- Ψβ-β-3'

538. 广西地中海贫血筛查血常规阳性标准是

A. MCV>83fl MCH<35pg

B. MCV<82fl MCH<26pg

C. MCV>82fl MCH>26pg

D. MCV>82fl MCH<35pg

539. 血红蛋白分析HbA2的正常值范围、HbF的正常值范围

A. 2.3-3.3%、<1.0%

B. 2.5-3.5%、<2.0%

C. 2.0-3.0%、<1.0%

D. 2.2-3.3%、<3.0%

540. 关于PCR产物电泳分析的陈述，下列哪个正确

A. 于负极点样，向正极泳动

B. 电泳后离点样点越远的为长片段，离点样点越近的为短片段

C. 产物片段越长，琼脂糖凝胶的浓度越大

D. 于正极点样，向负极泳动

541. 抽提的基因组DNA，用分光光度计检测所获得的OD260/280值合适范围为：

A. 1.0－1.4

B. 1.4－1.6

C. 1.7－2.0

D. 1.9－2.1

542. PCR检测实验的人员流动方向为：

A. 样本准备区→试剂准备区→扩增区→产物分析区

B. 试剂准备区→样本准备区→扩增区→产物分析区

C. 扩增区→样本准备区→试剂准备区→产物分析区

D. 试剂准备区→扩增区→样本准备区→产物分析区

543. 基因组DNA抽提方法不包括

A. 磁珠法

B. 酚氯法

C. 柱吸附法

D. TRIzol提取法

544. 位于ε-珠蛋白基因上游6－23kb区域内的红系特异性DNaseI高敏位点是

A．HS-40 B.LCR C.HS-20 D.3'HS1

545. 一对夫妇表型正常，妻子的弟弟为白化病（AR）患者。假设白化病基因在人群中为携带者的频率为1/60，这对夫妇生育白化病患儿的概率为（ ）

A.1/4 B.1/360 C.1/240 D.1/120

546. 人类α珠蛋白基因蔟定位在（ ）。

A.11p12 B.16p13 C.16q12 D.11q12

547. 人类β珠蛋白基因蔟定位在（ ）。

A.16p12 B.16q12 C.11p15 D.11q12

548. 异常血红蛋白病发病的机制主要是（ ）。

A. 点突变 B. 基因缺失

C. mRNA前体加工障碍 D. 融合基因

549. 由于基因融合引起的分子病为（ ）。

A. 镰状细胞贫血 B. β地中海贫血 C.血友病A D. Hb Lepore

550. 重型β地中海贫血的可能基因型是（ ）。

A. βA/βA B.δβ+/βA C.β0/β0 D.δβA/δβ0

551. 成人主要血红蛋白的分子组成是（ ）。

A. α2ε2 B. α2γ2 C. α2β2 D. α2ζ2

552. 若患者体内既含男性性腺，又含女性性腺，则为 （ ）。

A.男性 B.两性畸形 C.女性 D.假两性畸形

553. 下列那种遗传病可通过染色体检查而确诊（ ）。

A.苯丙酮尿症 B.白化病

C.血友病 D. Klinefelter综合征

554. 21三体型先天愚型的最主要的病因是：（ ）。

A. 母亲怀孕期间接触辐射

B. 母亲是47，XXX患者

C. 母亲年高，卵子减数分裂时，染色体不分离

D. 父亲年高，精子减数分裂时，染色体不分离

555. 对重型β地中海贫血和某些遗传性免疫缺陷患者治疗的有效方法是施行（ ）。

A.骨髓移植术 B.肝细胞移植术

C.红细胞移植术 D.白细胞移植术

556. 一个男孩是甲型血友病（XR）的患者，其父母和祖父母均正常，其亲属中不可能患此病的人是（ ）。

A.外祖父或舅父 B.姨表兄弟 C.姑姑 D.同胞兄弟

557. 先天性幽门狭窄是一种多基因遗传病，男性发病率为0.5%，女性发病率为0.1%，下列哪种情况发病率最高（ ）。

A.女性患者的儿子 B.男性患者的儿子

C.女性患者的女儿 D.男性患者的女儿

558. 经检测发现，某个体的细胞核中有2个X小体，表明该个体一个体细胞中有（ ）条X染色体

A. 1 B. 2 C. 3 D. 4

559. 下列核型中，属于亚二倍体是：（ ）

A、45 ,XO B、46 ,XX C、47,XXY D、47,XY, +21

560. 对人类而言，下列哪种细胞中有23条染色体：（ ）

A、精原细胞 B、初级精母细胞 C、初级卵母细胞 D、卵细胞或精细胞

561. 当基因为杂合子时，能够表现出来的性状为：（ ）

A、相对性状 B、遗传性状 C、显性性状 D、隐性性状

562. 一个基因或一对基因发生突变引起的疾病，叫：（ ）

A、遗传病 B、多基因病 C、单基因病 D、染色体病

563. 人类α珠蛋白基因蔟定位在（ ）。

A.11p12 B.16p13 C.16q12 D.11q12

564. 《产前诊断技术管理办法》自（ ）开始实施？

A2003年5月1日 B2002年5月1日

C2005年5月1日 D2004年5月1日

565. 对未取得产前诊断类母婴保健技术考核合格证书的个人，擅自从事产前诊断或超越许可范围的，由县级以上人民政府卫生行政部门（ ）；情节严重的，按照《中华人民共和国执业医师法》吊销其医师执业证书。构成犯罪的，依法追究刑事责任

A给予警告或者责令暂停一个月以上三个月以下执业活动

B给予警告或者责令暂停三个月以上六个月以下执业活动

C给予警告或者责令暂停六个月以上一年以下执业活动

D给予警告或者责令暂停一年以上两年以下执业活动

566. 基因表达时，遗传信息的基本流动方向是（ ）。

A.RNA→DNA→蛋白质 B.hnRNA→mRNA→蛋白质

C.DNA→mRNA→蛋白质 D.DNA→tRNA→蛋白质

567. 生殖细胞成熟过程中，染色体数目减半发生在哪个时期：（ ）

A、增殖期 B、生长期

C、第二次成熟分裂期 D、第一次成熟分裂期

568. 当基因为杂合子时，能够表现出性状来的基因是：（ ）

A、显性基因 B、等伴基因 C、隐性基因 D、致病基因

569. 嵌合体形成的原因是个别染色体不分离，这发生在：（ ）

A、减数分裂 B、第一次减数分裂

C、第二次减数分裂 D、胚胎卵裂

570. 两条非同源染色体同时发生断裂，断片交换位置后重接，结果造成（ ）。

A.缺失 B.倒位 C.易位 D.插入

571. 染色体结构畸变的基础是 ：（ ）

A.姐妹染色单体交换 B.染色体核内复制

C.染色体断裂及断裂之后的异常重排 D.染色体不分离

572. 家系调查的最主要目的是( )。

A．了解发病人数 B．了解疾病的遗传方式

C．了解医治效果 D．收集病例

573. 当一种疾病的传递方式为男性→男性→男性时，这种疾病最有可能是( )。

A. 从性遗传 B. 限性遗传

C. Y连锁遗传病 D. X连锁显性遗传病

574. 丈夫为红绿色盲，妻子正常且其家族中无患者，如再生育，子女患色盲的概率为( )。

A.1/2 B.1/4 C.2/3 D.0

575. 中间型α地中海贫血患者的基因型是（ ）。

A.αα/-α B.α-/-α C.- -/-α D.- -/- -

576. 正常人与重型β地中海贫血患者结婚，其子女患轻型β地中海贫血的可能性是( )。

A.0 B.1 C.1/4 D.1/2

577. 异常克隆是指至少有（ ）细胞含有相同的额外染色体或染色体结构异常，或至少有（ ）细胞均丢失同一号染色体。

A 2个，2个 B 2个，3个 C 3个，3个 D 3个，2个

578. 每个产前诊断病例至少有（ ）细胞的核型图像照相记录，电子版或者相片（ ）。

A 2个，保存5年 B 5个，永久保存 C 2个 ，永久保存 D 5个，保存5年

579. 人类染色体不包括（ ）

A: 中央着丝粒染色体 B:亚中着丝粒染色体

C:近端着丝粒染色体 D: 端着丝粒染色体

580. 临床上最常见的染色体数目异常是（ ）

A: 单体 B:三体 C: 三倍体 D: 四倍体

581. 染色体数目整倍性改变以（ ）倍体为主

A 单 B二 C三 D 四

582. 在一个随机杂交的群体中，多基因遗传的变异范围广泛，大多数个体接近于中间类型，极端变异的个体很少。这些变异产生是由（ ）。

A.多基因遗传基础和环境因素共同作用的结果

B. 遗传基础的作用大小决定的

C. 环境因素的作用大小决定的

D. 多对基因的分离和自由组合的作用的结果

583. 目前的遗传学观点认为，游离型21三体发生的最主要的病因是：（ ）。

A．父亲年高，精子减数分裂时21号染色体不分离

B．母亲年高，卵子减数分裂时，21号染色体不分离

C．母亲怀孕期间有病毒感染

D．母亲怀孕期间接触射线

584. 染色体上某一区段及其带有的基因一起丢失，从而引起变异的现象属于以下哪种染色体结构变异？（ ）

A. 染色体丢失 B.易位 C.倒位 D.缺失

585. 一对糖原沉淀病Ⅰ型携带者夫妇结婚后，其子女中可能患病的几率是（ ）。

A. 1 B. 1/2 C. 0 D. 1/4

586. α地中海贫血最主要的发病机制是（ ）。

A. 点突变 B. 基因缺失 C. mRNA前体加工障碍 D. 融合基因

587. 由于基因融合引起的分子病为（ ）。

A. 镰状细胞贫血 B. β地中海贫血 C.血友病A D. Hb Lepore

588. 人类遗传性疾病中不包括哪些（ ）。

A． 单基因遗传病 B．多基因遗传病 C．先天性疾病 D．染色体病

589. 人类染色体的化学成分主要的是：（ ）。

A. RNA和非组蛋白

B. RNA和组蛋白

C. DNA和组蛋白

D. DNA和非组蛋白

590. 染色质和染色体是（ ）。

A. 不同物质在细胞周期不同时期的存在形式

B. 同一种物质在细胞周期不同时期的两种存在形式

C. 同一种物质在细胞周期同一时期的两种形态表现

D. 同一种物质在细胞周期同一时期的两种叫法

591. 等位基因位于（ ）。

A. 常染色体

B. 性染色体

C. 非同源染色体

D. 同源染色体

592. 一对夫妇表型正常，妻子的弟弟为白化病（AR）患者。假设白化病基因在人群中为携带者的频率为1/60，这对夫妇生育白化病患儿的概率为（ ）

A.1/4 B.1/360 C.1/240 D.1/120

593. 成人血红蛋白以下列（ ）为主。

A. HbA2 B. HbA C. HbH D. Hb PortLand

594. 人类β珠蛋白基因蔟定位在（ ）。

A.16p12 B.16q12 C.11p15 D.11q12

595. 由多对基因和环境因素共同作用产生的疾病称为：（ ）

A、 遗传病 B、多基因病 C、单基因病 D、染色体病

596. 异常血红蛋白病发病的机制主要是（ ）。

A. 点突变 B. 基因缺失

C. mRNA前体加工障碍 D. 融合基因

597. 地中海贫血患者的贫血类型多为：

A. 大细胞性贫血

B. 正细胞正色素性贫血

C. 小细胞低色素性贫血

D. 单纯小细胞性贫血

598. 非输血依赖性地中海贫血（NTDT）发生铁过载的主要原因是

A. 红细胞异常增生

B. 长期重复输血

C. 红细胞无效生成

D. 铁调素生成增多

599. 关于地中海贫血说法错误的是

A. 分为TDT和NTDT

B. 通过"输血依赖性"区分地中海贫血及其严重程度

C. NTDT不需要终身输血维持生命

D. TDT不需要终身输血维持生命

600. 输血依赖性地中海贫血（TDT）发生铁过载的主要原因是

A. 体内铁生成增多

B. 重复输血

C. 红细胞无效生成

D. 铁调素生成减少
