# Supplementary material for: Performance and limitations of four large language models in genetic counseling for thalassemia
Source: Front Digit Health. 2026 Aug 11;8:1902480. doi: 10.3389/fdgth.2026.1902480 (PMC13464380; doi:10.3389/fdgth.2026.1902480)
Supplement: Supplementary file 3 [file Datasheet3.docx]

1. 如果夫妇双方没有地贫表现，那就不需要进行地贫筛查。

2. 夫妇双方只有一方为轻型地贫患者，建议常规做产前诊断。

3. 孕检时，女方未检出地贫，一般不需要男方来做地贫检查。

4. 所有地中海贫血患者都需要输血治疗。

5. 地中海贫血患者应多吃含铁丰富的食物。

6. 重型地中海贫血患者如果不治疗，通常难以存活到成年。

7. 夫妻双方都是地中海贫血基因携带者，胎儿一定患病。

8. 重型β地中海贫血一般在出生后 3-6 个月开始出现贫血等症状。

9. 输血治疗可以根治地中海贫血。

10. 新生儿筛查可以早期发现地中海贫血。

11. 地中海贫血是一种全球性的疾病，但主要影响地中海地区的人群。

12. 地中海贫血患者的血红蛋白合成异常，导致红细胞寿命缩短。

13. 地中海贫血患者不需要定期输血治疗。

14. 地中海贫血患者可以通过药物治疗来根治疾病。

15. 地中海贫血患者的基因治疗目前尚处于实验阶段。

16. 地中海贫血是一种先天性血液疾病。

17. 只有重型地中海贫血患者才会有贫血症状。

18. 地中海贫血患者的红细胞寿命通常比正常人短。

19. 铁剂是治疗地中海贫血的常规药物。

20. 地中海贫血患者的肝脾肿大由红细胞破坏过多引起。

21. 地中海贫血患者只能通过输血治疗。

22. 血红蛋白电泳可以确诊地中海贫血的类型。

23. 轻型地中海贫血患者一般不需要特殊治疗。

24. 中间型地中海贫血病情相稳定，不需要定期随访。

25. 血红蛋白异常一定是地贫。

26. 地中海贫血只会影响血红蛋白，不会影响其他身体器官。

27. 婚前检查可以有效降低地中海贫血患儿的出生率。

28. 轻型地中海贫血患者的血红蛋白电泳结果一定正常。

29. 地中海贫血患者不需要预防接种。

30. 造血干细胞移植治疗地中海贫血一定能成功。

31. 地中海贫血患者的贫血程度与基因缺陷类型有关。

32. 所有地中海贫血患者都需要特殊治疗。

33. 地中海贫血患者一定有家族遗传史。

34. 血常规正常可以排除地中海贫血。

35. 输血是治疗地中海贫血的唯一方法。

36. 轻型地中海贫血患者不影响正常生活。

37. 基因检测可以准确诊断所有类型的地中海贫血。

38. 夫妻一方是地中海贫血患者，孩子一定会患病。

39. 地中海贫血在人群中的发病率是固定不变的。

40. 所有地中海贫血都需要进行基因诊断。

41. 轻型地中海贫血生活无任何影响。

42. 输血是治疗重型地中海贫血的唯一方法。

43. 基因治疗有望根治地中海贫血。

44. 地中海贫血患者血常规中红细胞形态无异常。

45. 中间型地中海贫血通常比轻型地中海贫血病情严重。

46. 地中海贫血的诊断不需要结合临床症状。

47. 目前地中海贫血的治疗主要是缓解症状。

48. 孕妇进行地中海贫血筛查没有意义。

49. 地中海贫血是一种传染性疾病。

50. 所有地中海贫血患者都有明显的临床症状。

51. 地中海贫血患者不能进行运动。

52. 地贫基因检测只能检测一种基因突变类型。

53. 孕妇进行地中海贫血筛查对胎儿没有影响。

54. 地中海贫血患者输血治疗可以无限制进行。

55. 中国南方地区人群都需要进行地中海贫血筛查。

56. 地中海贫血患者不需要补充叶酸。

57. 地中海贫血防控可以有效降低重型地贫患儿的出生率。

58. 地中海贫血只发生在特定的种族人群中。

59. 所有的地中海贫血都是由基因大片段缺失引起的。

60. 地贫患者的贫血程度与地贫类型密切相关。

61. 产前诊断可以有效预防重型地中海贫血患儿出生。

62. 地中海贫血患者输血治疗后就不需要其他治疗了。

63. 单独使用血红蛋白组分或血常规分析漏诊率低。

64. α-地中海贫血使用血红蛋白电泳漏诊主要是轻型和静止型。

65. 如发现标本有溶血、污染等可能影响结果准确性的现象，应在报告单上注明。

66. EQA的目的为了建立实验室间的比较——实验室间检验结果的可比性。

67. 实验室检测室间质量评价标本的次数应与常规检测患者样品的次数不一致。

68. 实验室可以将室间质量评价标本送至另一实验室进行检测。

69. 选择性减胎术是指在多胎妊娠中有选择性的终止发育不良、畸形或者过多的胎儿继续发育，以减少孕妇及胎儿并发症，确保健康胎儿正常存活和发育的技术。

70. 产前筛查、产前诊断技术的应用，应当以医疗保健为目的，符合国家有关法律规定和伦理原则，由经资格认定的医务人员在经许可的医疗保健机构中进行，并遵循“知情同意，自愿选择”的原则。

71.地中海贫血是一种全球性分布的遗传病，在热带和亚热带地区高发，包括地中海地区、东南亚、中国南方等。

72.常规基因检测可以诊断绝大多数已知突变类型的地中海贫血，但对于一些罕见或复杂的结构变异可能存在漏检风险。

73.近端着丝粒染色体具有非常短的短臂，短臂末端通常带有随体和随体柄。

74.绝大多数Down综合征男性患者因性腺发育不良而无生育能力；大部分女性患者有月经，部分具有生育能力，但其后代有高概率罹患同病。

75.β地中海贫血最主要的发病机制是导致β珠蛋白链合成减少或无合成的各种点突变，其具体途径多样，包括转录、RNA加工（剪接）、翻译及翻译后水平缺陷。

76.单基因病（已发现超7000种）是人类遗传病中种类最多的一类；而多基因病是人群中患病率最高、最常见的一类。

77.夫妇双方只有一方为轻型地贫患者，无须常规做产前诊断。

78.在孕前或孕期检查中，只要有一方是地贫基因携带者，另一方就必须进行地贫筛查和基因检测。

79.中间型地中海贫血的病情并不总是稳定，且必须进行定期随访。

80.地中海贫血在人群中的发病率并非固定不变，它是一个受多种因素动态影响的变量，主要呈现显著的地域、种族和时间差异。

81. 近端着丝粒染色体没有短臂。

82. 卫生部《胎儿染色体核型分析技术规范》要求，羊水细胞培养成功率不得低于90%。

83. 标记染色体用mar表示，mar2表示是2号染色体衍生而来的标记染色体。

84. Down综合征患者都具有通贯掌。

85. 猫叫综合征患者缺失片段为5p15，多数病例是父母生殖细胞中新发生的染色体结构畸变所引起的。

86. 由某些单基因的缺陷和环境因素可造成两性畸形，而染色体核型分析结果是正常的。

87. 若Y染色体小于21号染色体，则可描述为Yqh+。

88. 如果一套单倍体染色体可显示500条带，这种染色体称为高分辨率显带染色体。

89. 易位型先天愚型一般常见于年龄较大的父母所生的子女。

90. 用NaOH处理标本后，再用Giemsa染剂，可使着丝粒部分深染。

91. 一套单倍染色体可显示550条或更多的带纹，这种染色体称为高分辨显带染色体。

92. 在符合标准的标本、培养、制片、显带情况下，核型分析的准确率不得低于95%。

93. 标记染色体（mar）是指不能被常规的显带方法分辨或明确识别的发生结构畸变的染色体。

94. 46,XY,del(2)(p31)表示2号染色体短臂3区1带断裂造成了该处以远的末端缺失。

95. 21/21平衡易位携带者其婚后所生育活婴将100%为Down综合征患儿。

96. 性染色体的畸变可导致两性畸形的发生，但并非两性畸形都是由于性染色体异常引起。

97. 若核型分析时计数100个核型，发现45,X,[50],46,XX[20]，47,XXX[30]则核型写法为45,X,[50]/46,XX[20]/47,XXX[30]。

98. 若Y染色体的长度为第2号染色体长度的1/3以下，则描述为Yqh-。

99. 13-三体综合征发病率约为 1/5000～1/25000，女性患儿略多于男性，发病率与母亲年龄增大有关。

100. 正常情况下，α2基因比α1基因的功能更强，所以α2基因的突变比α1基因突变表型的影响更大。

101. 目前发现的中国人α-地贫缺失突变中，有几种大片段突变除涉及二个α珠蛋白基因缺失外，还同时丢失了胚胎期表达的ζ基因，如(- -MED/)和(- -FIL/)。

102. 某些东南亚缺失型新生儿脐血中ζ-珠蛋白肽链的含量高于正常新生儿。

103. CS与QS突变引起的血液学表型变化较–3.7、–4.2、WS突变要轻一些。

104. 分子诊断的技术策略，对于α-地贫，首先检测常见的大片段缺失，其次检测少见的点突变。

105. 中间型地贫一般为无症状个体，一般需做基因分析才能检查出来。

106. Hb Bart’s水肿胎的心/胸比率增大定义为在孕12~17周≥0.5。

107. N显带，用硝酸银染色，可使染色体的随体及核仁形成区呈现出特异性的黑色银染物。

108. 染色质通过多级螺旋包装形成染色体，经过四级包装，理论上染色体中DNA长度被压缩了约8 000～10 000倍。

109. 精子染色体数目为二倍体。

110. 当母亲年龄增大时，所生子女的体细胞中某一序号染色体有三条的情况要多于一般人群。

111. 核型为45,XX,der(21;21)(q10;q10)平衡易位携带者，其婚后所生育的活婴都是Down综合征患儿。

112.X三体综合征（47,XXX）在新生女婴中发病率约为1/1000。绝大多数患者智力正常，但有特定学习障碍的风险；随着X染色体数目增加（如48,XXXX，49,XXXXX），智力低下和发育迟缓的风险与严重程度会显著增高。113. 染113.染色体制备过程中使用的低渗液主要是0.075ml/L氯化钠溶液。

114. 46,XY,upd(15)mat，表示男性核型，两条15号染色体均来自父方。

115. 18三体综合征患儿95%有先天性心脏病，是婴儿死亡的主要原因。

116. 最常用的染色体显带技术是Q显带。

117. 染色体和染色质是一种由DNA、组蛋白、非组蛋白及RNA等组成的核蛋白复合物。

118. 卫生部《胎儿染色体核型分析技术规范》要求，绒毛染色体核型分析异常，必要时做羊水或脐血复核。

119. 亚二倍是在二倍体的基础上减少一组染色体。

120. Down综合征男性患者常有隐睾，无生育能力，女性患者也通常无月经，没有生育能力。

121. 一般而言，克氏症患者核型中X染色体数量越多，表现症状越严重。

122. 46,XY,upd（15）pat表示男性核型，两条15号染色体均来自母方。

123. 大多5p-染色体综合征患者是由父母生殖细胞中新发的染色体结构畸变所引起的。

124. 中华人民共和国《母婴保健法》规定，对于依法接受终止妊娠或者结扎手术的，应当给予免费服务。

125. 开展产前诊断技术的医疗保健机构不得擅自进行胎儿的性别鉴定。怀疑胎儿可能为伴性遗传病，需要进行性别鉴定的，由省、自治区、直辖市人民政府卫生行政部门指定的医疗保健机构按照有关规定进行鉴定。

126. 若患者体内既含男性性腺，又含女性性腺就称为假两性畸形。

127. 1959年Lejune发现的第一例染色体病为唐氏综合症。

128. 细胞核内能被酸性染料染色的物质，称为染色质。

129. 染色体多态性在人群中广泛存在。

130. 罗伯逊易位平衡携带者的体细胞染色体数目为45条，其遗传物质基本平衡，表型正常。

131. 平衡易位的患者容易发生习惯性流产。

132. 45，XX，der（14；21）（q11；q11）表示一名女性少了一条染色体，无法存活。

133. Klinefelter综合征的临床表现有身材高大、性格暴躁、常有攻击性行为。

134. 根据中华人民共和国《母婴保健法》规定，男女双方精神病检查不属于孕产期保健内容。

135. 未取得产前诊断类母婴保健技术考核合格证书的个人，擅自从事产前诊断或超越许可范围的，由县级以上人民政府卫生行政部门给予警告或者责令暂停六个月以上一年以下执业活动；情节严重的，按照《中华人民共和国执业医师法》吊销其医师执业证书。构成犯罪的，依法追究刑事责任。

136. “猫叫”综合征与人类第4号染色体的部分缺失有关。

137. 平衡易位的患者容易发生智力低下。

138. 最常用的染色体显带技术是C显带。

139. 47，XXY综合症患者表型一般正常，无尿道下裂、隐睾、睾丸发育不全症状。

140. 46,XX,6qh+属于染色体多态性。

141. Lyon假说认为，一旦某一特异的细胞内X染色体失活，那么由此细胞而增殖的所有子代细胞也总是这一个X染色体失活。

142. 目前最常用的染色体显带技术是R显带。

143. 中国卫生部《胎儿染色体核型分析技术规范》要求，脐血细胞培养成功率不得低于95%。

144. 单体型是指在二倍体基础上减为一条染色体。

145. 染色体断裂后，断片颠倒180°后重新接到断端造成的染色体异常叫重复。

146. 13-三体、18-三体综合症患儿临床特征都有相似的特殊握拳姿势和摇椅样畸形足，但与21-三体不同，其发生于母亲年龄无关。

147. XYY综合症患者表型一般正常，偶尔可见尿道下裂、隐睾、睾丸发育不全并有生精进程障碍和生育力下降。

148. 若Y染色体的长度为第22号染色体长度的½以下，则描述为Yqh+。

149. 如果一套单倍体染色体可显示550条带，这种染色体称为高分辨显带染色体。

150. 染色体多态现象是一种较稳定的结构变异，按孟德尔方式遗传，可以帮助判断子女与其父母的真实关系。

151.第1、9、16号和Y染色体的着丝粒或邻近区域在G显带下通常呈深带，是识别这些染色体的重要标志。而在C显带下，所有染色体的着丝粒区域均呈深染，其中第1、9、16号染色体的次缢痕区和Y染色体长臂的深染块更为显著。

152.13-三体和18-三体综合征患儿具有相似的特殊握拳姿势和摇椅样畸形足等临床特征。与21-三体综合征一样，其发生风险均与母亲年龄增大呈正相关，但总体发病率低于21-三体。

153.46,XY,upd（15）pat表示男性核型，两条15号染色体均来自父方。

154. 46,XY,upd(13)mat，表示男性核型，两条13号染色体均来自母方。

155.46,XX,9qh+属于染色体多态性。

156.精子染色体数目为单倍体。

157.CS与QS突变引起的血液学表型变化较–3.7、–4.2、WS突变要重一些。

158.WS 突变携带者多为静止型，血常规常无明显异常，仅基因检测可发现。

159.-3.7、-4.2 突变属于缺失型α⁺地贫，仅造成 α 珠蛋白基因的部分缺失，α 链合成减少的程度相对温和。

160.CS、QS 突变均为非缺失型α⁺地贫，是 α2 基因的错义突变。α2 基因的表达量显著高于 α1 基因，因此这类突变会导致 α 链合成减少的幅度更大，进而引发更明显的血液学异常。

161. 胎儿血红蛋白以HbA为主。

162. - -/α-是标准型α地中海贫血的基因型。

163. 绒毛取样的最佳时期是5-7周。

164. 在多基因遗传病中，遗传因素确实是主要的影响因素，但并非唯一因素。其核心特征是由多个微效基因的共同作用，并通常与环境因素相互作用，导致疾病发生。

165. 双雌受精或双雄受精的结果是产生了嵌合体。

166. 异常血红蛋白病发病的机制主要是染色体缺失。

167. 由于X染色体随机失活，正常女性的体细胞内将只有一条X染色体具有活性，对于核型为45, X的个体来说，由于其X染色体不失活，将不表现出疾病表型。

168. 单基因病是人类遗传病中种类最多的一类，而多基因病则是人群中最常见、患病人数最多的遗传病类型。

169. 染色体不分离发生在第一次卵裂时，会形成具有两个细胞系的嵌合体；若发生在后续卵裂，则可能形成两种或三种细胞系的嵌合体。

170. 临床上诊断PKU患儿的首选方法是生化检查。

171. 当遗传病发展到各种症状已经出现，机体器官已经受到损害，这时治疗的作用就仅限于基因治疗。

172. β地中海贫血最主要的发病机制是mRNA前体加工障碍。

173. 染色体数目整倍性改变的个体多在婴幼儿期死亡。

174. 咨询医师提出可供咨询者选择的若干方案，并要陈述各种方案的优缺点，并代替咨询者做出决定。

175. 等臂染色体一般是由于着丝粒分裂异常造成的。

176. 染色体上某一区段连同它带有的基因顺序发生180°倒转，从而引起变异的现象属于倒位。

177. 导致临床常见嵌合体的染色体不分离，大多发生在受精卵第一次卵裂之后的某次有丝分裂。绝大多数可存活的嵌合体中都包含正常细胞系。不分离发生得越晚，异常细胞系比例越低，分布越局限，患者临床症状通常也越轻微

178. 罗伯逊易位携带者形成正常配子的几率为0。

179. 产前诊断主要从细胞培养、染色体检查、分子诊断三个方面进行。

180. HbF的分子结构是α2γ2。

181. 染色体病是令人棘手的一类遗传病，目前无法根治，但改善症状很容易。

182. 在胚胎发育过程中，染色体不分离发生得越晚，正常二倍体细胞系的比例越小。

183. 染色体不分离多发生在减数第二次分裂时同源染色体的不分离。

184. 染色体病表型的轻重程度主要取决于染色体上所累及基因的多少。

185. Rh溶血病很少发生于第二胎，因为进入母体的胎儿细胞数量少，产生的抗体也少，不至于引起胎儿或新生儿溶血。

186. β地中海贫血属于珠蛋白生成障碍性贫血的疾病。

187. 致病基因已知、并且定位基本确定，其结构序列清楚的疾病，可以考虑采取基因连锁检测方法进行基因诊断。

188. 患者身体特征、健康情况没有患者的知情同意，不得透露给第三方，以确保患者的隐私权。

189. 线粒体遗传病是由线粒体突变导致的遗传病，是伴随父亲线粒体向下传递的。

190. 由于ε-珠蛋白基因在发育过程中持续的时间比ζ-珠蛋白基因长，部分新生儿的脐血中可能检测到微量的Hb Portland。

191. 定义不明确的遗传病也适用于遗传筛查。

192. 静止型α-地贫和α0-地中海贫血杂合子通婚后则有可能生育较严重的Hb H病患儿。

193. 珠蛋白基因簇以外的QTL位点，包括BCL11A基因和HBS1LCMYB间隔区的多态性，可以通过增加Hb A2的表达量，参与修饰β-地贫的表型。

194. 静止型α-地贫为无症状个体，一般需做基因分析才能检查出来。

195. 分析中质量控制包括标本接收、登记、前处理、分析、室内质控、结果审核。

196. SOP是作业人员的工作准则，将作业人员的工作予以说明与规范，以达作业的一致性与标准性。

197. 取羊水标本最佳时间为9-12周。

198. 21-三体型先天愚型的最主要的病因是母亲怀孕期间接触辐射。

199. 特纳综合征的染色体核型为45,X。

200. 46,XY,1qh+属于染色体多态性。

201. 由于ζ-珠蛋白基因的表达可持续至围产期，而ε-珠蛋白基因表达较早停止，因此在重型α-地中海贫血胎儿或新生儿的血液中可检测到Hb Portland；而在正常足月新生儿的脐血中，使用常规方法通常无法检出。

202. Klinefelter综合征的染色体核型为47,XYY。

203. 外周血染色体主要来源于淋巴细胞。

204. 染色体G显带中着丝粒位置通常显浅带。

205. 基因自由组合定律的细胞学基础是非同源染色体自由组合。

206. 染色体制备中常用甲醇和冰醋酸来配制固定液。

207. 造血干细胞移植是治愈重型地中海贫血的有效方法。

208. 一个常染色体平衡易位携带者最常见的临床表现是智力低下。

209. 轻度α地贫靠血常规能检查出来。

210. 三倍型是指在二倍体基础上增加一条染色体。

211. 遗传病是遗传物质改变所导致的疾病。

212. DNA的复制是连续进行的。

213. 标准的遗传密码在几乎所有生物（病毒、原核生物、真核生物的核基因组）中都是通用的，这构成了生命同源性和生物技术的基石；但真核生物的线粒体等少数细胞器拥有略有不同的遗传密码变体。

214. 真核细胞中染色体主要是由DNA和RNA组成。

215. 染色体结构畸变的基础是断裂及断裂后的变位重接。

216. 一夫妇同为静止型α地中海贫血患者，他们生育一个轻型α地中海贫血患者的可能性是1/2。

217. 寡核苷酸探针杂交法最适合于诊断下列哪种类型的遗传病：点突变型单基因遗传病。

218. 唇裂的发生完全由遗传因素决定发病。

219. 根据中华人民共和国《母婴保健法》规定，医师根据《母婴保健法》提出医学意见，当事人必须无条件执行。

220. 中华人民共和国《母婴保健法》规定孕产期保健内容包括母婴保健指导、胎儿保健、孕产妇保健及儿童预防接种。

221. 5p-综合征，又称为猫叫综合征，患儿具特有的猫叫样哭声，为5号染色体长臂缺失引起。

222. 断裂基因中的内含子和外显子的关系是固定不变的。

223. 引起镰状细胞贫血的β珠蛋白基因突变的方式是错义突变。

224. 成人血红蛋白以HbA2为主。

225. 血友病B缺乏的凝血因子是Ⅷ。

226. 产前筛查结果为低风险的，说明此结果可完全排除患遗传病的可能性。

227. 当基因结构变化未知时，可考虑进行基因连锁检测方法进行基因诊断。

228. 目前进行的基因治疗属于体细胞基因治疗。

229. 严禁使用技术手段对胎儿进行性别鉴定，但医学上确有需要的除外。

230. 由于突变使编码密码子形成终止密码，此突变为无义突变。

231.血友病A缺乏的凝血因子是Ⅷ。

232.血友病B是由于编码凝血因子Ⅸ的基因发生突变，导致该因子数量缺乏或功能缺陷而引起的遗传性出血性疾病。

233.成人血红蛋白以HbA为主，HbA₂是含量少但具有重要诊断意义的成分。

234.一夫妇同为静止型α地中海贫血患者，他们生育一个轻型α地中海贫血患者的可能性是1/4。

235.染色体病是一类复杂的遗传病，目前尚无法在染色体水平实现根治。其临床表现多样且常累及多系统，改善症状需要早期、持续且多学科参与的综合干预与管理，过程通常并不容易。

236.在胚胎发育过程中，染色体不分离发生得越晚，正常二倍体细胞系的比例越大。

237.部分β-地中海贫血和杜氏肌营养不良症是由无义突变导致蛋白质合成提前终止而引起的。

238.羊膜腔穿刺术进行产前诊断的最佳操作时间为妊娠16-20周，最晚不宜超过22⁺⁶周。

239.常染色体平衡易位携带者本人通常表型正常。其主要风险是可能导致后代染色体不平衡，从而引起流产或生育出智力低下、多发畸形的患儿。

240.染色体不分离可发生在减数第一次分裂，导致同源染色体不分离。
